# Antioxidant Activities of Phenolic Metabolites from *Flemingia philippinensis* Merr. et Rolfe and Their Application to DNA Damage Protection

**DOI:** 10.3390/molecules23040816

**Published:** 2018-04-02

**Authors:** Jeong Yoon Kim, Yan Wang, Yeong Hun Song, Zia Uddin, Zuo Peng Li, Yeong Jun Ban, Ki Hun Park

**Affiliations:** 1 Division of Applied Life Science (BK21 plus), IALS, Gyeongsang National University, Jinju 52828, Korea; foryou6633@gmail.com (J.Y.K.); yh0126@hanmail.net (Y.H.S.); ziauddinrph@gmail.com (Z.U.); dameng@126.com (Z.P.L.); banyoung972@naver.com (Y.J.B.); 2 College of Food and Biological Engineering, Qiqihar University, Qiqihar 161006, China; pitwang05@163.com

**Keywords:** *Flemingia philippinensis*, phenolic metabolites, electron spin resonance spectroscopy, radical scavenging activity, DNA damage protective effects

## Abstract

*F. philippinensi*s Merr. et Rolfe has been cultivated on a large scale and is widely consumed by local inhabitants as an important nutraceutical, especially against rheumatism which has a deep connection with antioxidants. In this study, a total of 18 different phenolic metabolite compounds in *F. philippinensi*s were isolated and identified, and evaluated for their antioxidant and DNA damage protection potential. The antioxidant activity of the 18 identified compounds was screened using DPPH, ORAC, hydroxyl and superoxide radical scavenging assays. The antioxidant potential of the compounds was found to differ by functionality and skeleton. However, most compounds showed a good antioxidant potential. In particular, seven of the identified compounds, namely, compounds **2**, **3**, **6**, **10**, **11**, **15** and **16**, showed significant protective effects on pBR322 plasmid DNA against the mutagenic and toxic effects of Fenton’s reaction. The most active compound, compound **2**, displayed a dose-dependent DNA damage protection potential in the range of 7.5~60.0 μM. The DNA damage protective effect of the identified compounds was significantly correlated with the hydroxyl radical scavenging activity. Compounds that exhibited effective (IC_50_ = 5.4~12.5 μg/mL) hydroxyl radical scavenging activity were found to be the ones with higher DNA damage protection potential.

## 1. Introduction

Reactive oxygen species (ROS) are reactive species with a lone pair electron that is able to mainly attack double bonds in biomolecules of living organisms [1]. They include various oxygen centered free radical derived from superoxide (O_2_^•−^) and non-radical derivatives, like hydrogen peroxide (H_2_O_2_) [2,3]. They are formed by not only a normal cellular metabolism, but also external environmental factors, such as stress, overeating and UV radiation [4,5]. A low concentration of ROS is rather essential for several physiological processes and can be easily removed by endogenous antioxidants, such as superoxide dismutase and catalase [6]. But excess ROS can induce mutation, cell death, aging and major human degenerative diseases [7,8]. Antioxidants are defined as having the ability to quench free radicals to form stable species with paired electrons, to maintain redox balance before ROS species can damage important biomolecules [9]. Antioxidants play important roles in the protection of the body against oxidative cell damage that can lead to Alzheimer’s disease, cancer, heart disease and chronic inflammation [10,11,12].

Phenolic metabolites in plants are the main sources of antioxidant substances, because they have electron donating potential due to their resonance structure. Antioxidant potential can be estimated by several kinds of radical sources, which each have their own advantages in allowing us to understand a certain characteristic of antioxidant [13]. For example, the DPPH reaction attributes more weight to single electron transfer (SET) and can even accommodate hydrophobic compounds. The oxygen radical absorbance capacity (ORAC) assay uses peroxyl radicals, which are a better model of antioxidant reactions in the human body. These are quenched by the hydrogen atom transfer (HAT) mechanism [14]. In particular, the hydroxyl radical is the most reactive and tends to react with nucleotides and subsequently, with whole DNA molecules [15]. The hydroxyl radical may lead to the single-stranded cleavage of supercoiled plasmid DNA, resulting in a relaxed open-circular form [16]. This change can be screened by gel electrophoresis. The potential of an antioxidant to protect against plasmid DNA damage may lead to the prevention of carcinogenesis, mutagenesis and a number of genetic disorders [17,18]. However, although there are a few reports on the antioxidant potential of the whole extract of *Flemingia* species, no study has measured the antioxidant effects of individual compounds [19,20].

*Flemingia philippinensis* Merr. et Rolfe is a polyphenol rich plant that is particularly rich in isoflavone derivatives because this plant belongs to the legume family [21]. This plant is cultivated on a large scale and is widely consumed by local inhabitants as an important nutraceutical [22]. Its bioactive metabolites possess anti-inflammatory, antiestrogenic and immunosuppressive activities including enzyme inhibitions against neuraminidase, tyrosinase and protein tyrosine phosphatase 1B (PTP1B) [23,24,25,26]. Importantly, it has been used to improve rheumatism and bone mineral density, which have deep correlations with the effects of antioxidants [27].

Based on the above, this study focused on the isolation and identification of about 18 different phenolic metabolites from *F. philippinensis*. The isolated compounds were further examined for their antioxidant potential using four different assay systems employing electron spin resonance spectroscopy (ESR) and fluorescence. Also, the DNA damage protection potential of all the isolated compounds was examined to find out the potential candidates that render DNA damage protection.

## 2. Results and Discussion

### 2.1. Total Content of Phenolics and Flavonoids

*F. philippinensis* Merr. et Rolfe has been used traditionally for rheumatism and inflammation which occurs by oxidative cell damage [28]. This plant may have the antioxidant potential to delay oxidative stress. However, there have been no logical reports explaining the traditional function of this plant based on its antioxidant potential. In this study, the total phenol and flavonoid concentrations, including the DPPH scavenging activity, representing the antioxidant potential in 0–80% ethanol extracts of *F. philippinensis*, were initially quantified. The high potency ethanol extract (80% EtOH) recorded about 4947 mg GAE/100 g total phenols, 3058.1 mg QE/100 g total flavonoids and 78% inhibition of DPPH at 100 μg/mL (Table 1). This result encouraged us to further isolate and identify the polyphenolic compounds of *F. philippinensis* that are responsible for their antioxidant potential. 

**Figure 1:**
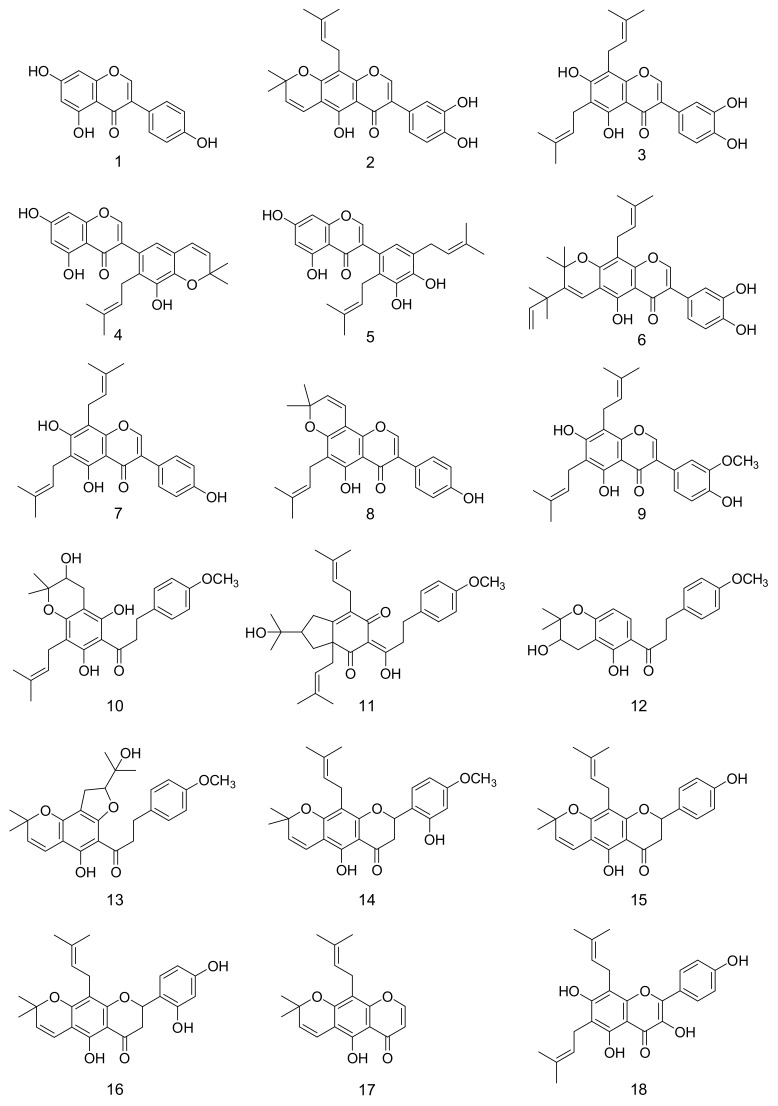
Chemical structures of phenolic metabolites (**1**–**18**) from *F*. *philippinensis.*

The 80% EtOH extract was further fractionated and purified by repeated column chromatography with silica gel, C18 gel and sephadex LH-20. We isolated about eighteen polyphenolic compounds (**1**–**18**), as shown in Figure 1. Through spectroscopic analysis, the isolated compounds were identified [19,20,21,22,23,24,26] as genistein (**1**), auriculasin (**2**), 6,8-diprenylorobol (**3**), 5, 7, 3′-trihydroxy-2′-(3-methylbut-2-enyl)-4′,5′-(3,3-dimethylpyrano)iso-flavone (**4**), 5,7,3′,4′-tetra-hydroxy-2′,5′-di(3-methylbut-2-enyl)isoflavone (**5**), flemiphilippinin A (**6**), 8-γ,γ-dimethyl-allylwighteone (**7**), osajin (**8**), flemingsin (**9**), fleminchalcone A (**10**), flemiphilippinone A (**11**), fleminchalcone B (**12**), fleminchalcone C (**13**), khonklonginol H (**14**), lupinifolin (**15**), flemichin D (**16**), eriosematin (**17**) and 6,8-diprenyl-kaempferol (**18**). Their individual spectroscopic data were summarized and are presented in the Supplementary Material.

### 2.2. DPPH Radical Scavenging Activity by ESR

**Figure 2:**
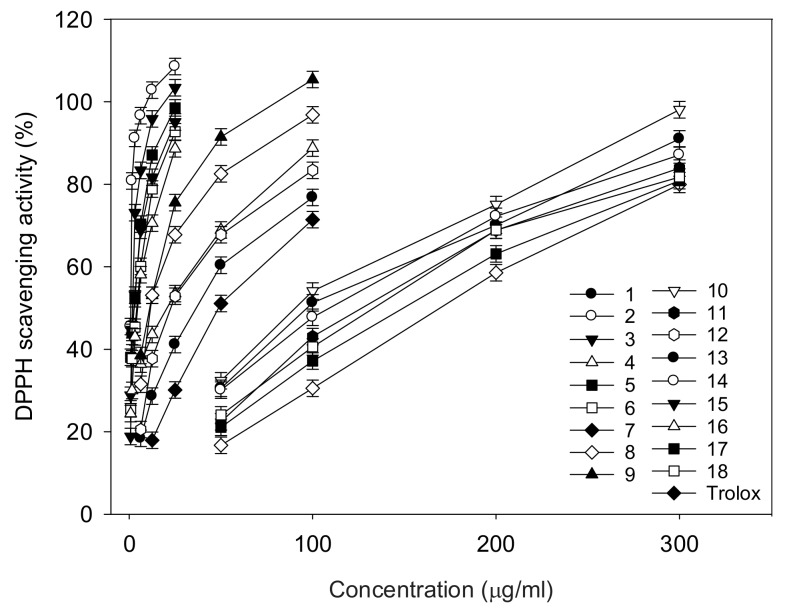
DPPH radical scavenging activity of compounds **1**–**18** and Trolox.

This study used four different in vitro antioxidant assays: DPPH, ORAC, hydroxyl radical scavenging activity and superoxide anion radical scavenging activity. DPPH is an N-centered radical, while the remaining are O-centered ones. Radicals are most commonly quenched by two mechanisms—transfer of either a hydrogen atom or an electron to convert the radical [14]. Hence, different antioxidant assays have their own conceptual and technical limitations in identifying the best sources of antioxidants. An analysis of the test material using different antioxidant assays would render a reliable result and that is why we conducted four different in vitro antioxidant assays in this study. All of the identified polyphenolic compounds of *F. philippinensis* had potent antioxidant actions against DPPH radicals (Table 2 and Figure 2), mediated by the single electron transfer mechanism. The antioxidant potential detected by ESR considerably varied between 0.9 and 164.5 μg/mL of IC_50_, according to the chemistry of the skeleton, including the number and position of the hydroxyl radical. The isoflavones (compounds **2**, **3**, **5** and **6**) with catechol motif in B-ring were found to be the most active antioxidants, with 0.9, 2.0, 2.8 and 4.5 μg/mL of IC_50_, respectively. Flavanone **16** with resocinol motif also showed a high potency with 4.7 μg/mL of IC_50_. The prenyl group in the A-ring of isoflavone did not influence the antioxidant potential: genistein (IC_50_ = 37.3 μg/mL) vs. prenylated derivatives of compound **7** (IC_50_ = 50.6 μg/mL). The C3-OCH_3_ functionality in the B-ring was able to improve the antioxidant activity, because compound **9** (IC_50_ = 10.4 μg/mL) was five times more effective than compound **7**. In the flavanone skeleton, C6-OH in the B-ring contributed more to the antioxidant effect than C4-OH: compound **15** (22.8 μg/mL) vs. **16** (4.7 μg/mL). Three chalcones (compounds **10**, **12** and **13**), having no hydroxyl group on the B-ring, showed low DPPH scavenging effects, with IC_50_ of 58.2–96.8 μg/mL.

### 2.3. Peroxyl Radical Scavenging Activity by ORAC

The ORAC assay offers several unique aspects over the DPPH assay. It uses the peroxyl radical which is a better model of antioxidant reactions with reactive oxygen species (ROS) in foodstuffs. The ORAC activity follows the hydrogen atom transfer mechanism and is based on the oxidation of fluorescein by the peroxyl radical [14]. As presented in Table 2, all identified compounds (**1**–**18**) displayed potent antioxidant activity, according to the ORAC assay, in the range 0.3–32.3 μmol TE/g. Most compounds, like **2**, **3**, **5** and **6**, that showed significant DPPH scavenging activity also displayed substantial ORAC potentials of about 11.2, 10.5, 15.6 and 2.5 μmol TE/g, respectively. However, the tendencies of antioxidant potentials were very different in regard to DPPH quenching effects. The important DPPH quenchers, such as compounds **6** and **16**, with 4.5 and 4.7 μg/mL, respectively, showed relatively low activity, producing values of 2.5 and 4.6 μmol TE/g with the ORAC system, respectively. Compound **4** (IC_50_ = 21.8 μg/mL) was twenty times less effective than compound **2** (IC_50_ = 0.9 μg/mL) in the DPPH assay, but compound **4** (32.3 μmol TE/g) was only three times more effective than compound **2** (11.2 μmol TE/g) in ORAC assay. This could be due to the contrast between the complete atom transfer mechanism involved in the ORAC system and the electron transfer mechanism in the DPPH system.

### 2.4. Superoxide Anion Radical Scavenging Activity by ESR

**Figure 3:**
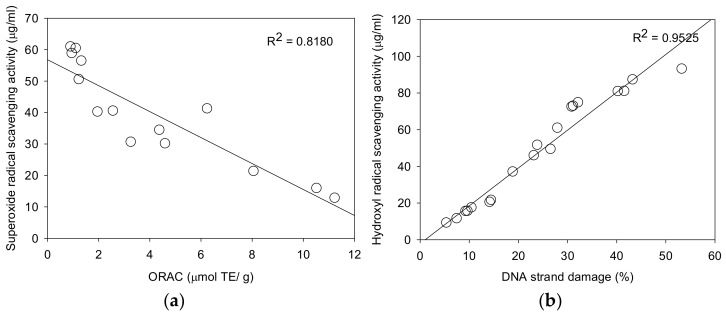
(**a**) Correlation between ORAC values and superoxide radical scavenging activity; (**b**) correlation between hydroxyl radical scavenging activity and DNA strand damage.

Superoxide anion radical (O_2_^•−^) is known as an important precursor of more active oxidative species and develops into hydroxyl radicals and alkyl radicals that attack biological molecules [3]. These radicals were easily detected with spin trap DMPO by using ESR. Superoxide anion radicals were generated from ultraviolet irradiation of photochemicals (riboflavin) in EDTA solution [29]. All identified compounds (**1**–**18**) displayed significant superoxide anion radical scavenging activity in a dose-dependent manner. Compounds **2**, **3**, **4** and **9** were found to be the most active antioxidants, with IC_50_ of 12.8, 15.9, 21.5 and 21.3 μg/mL. The superoxide radical scavenging activity of the identified compounds (**1**–**18**) showed a significant correlation (*R*^2^ = 0.8180) with superoxide radical scavenging capacity/ORAC (Figure 3a).

### 2.5. Hydroxyl Radical Scavenging Activity by ESR

**Figure 4:**
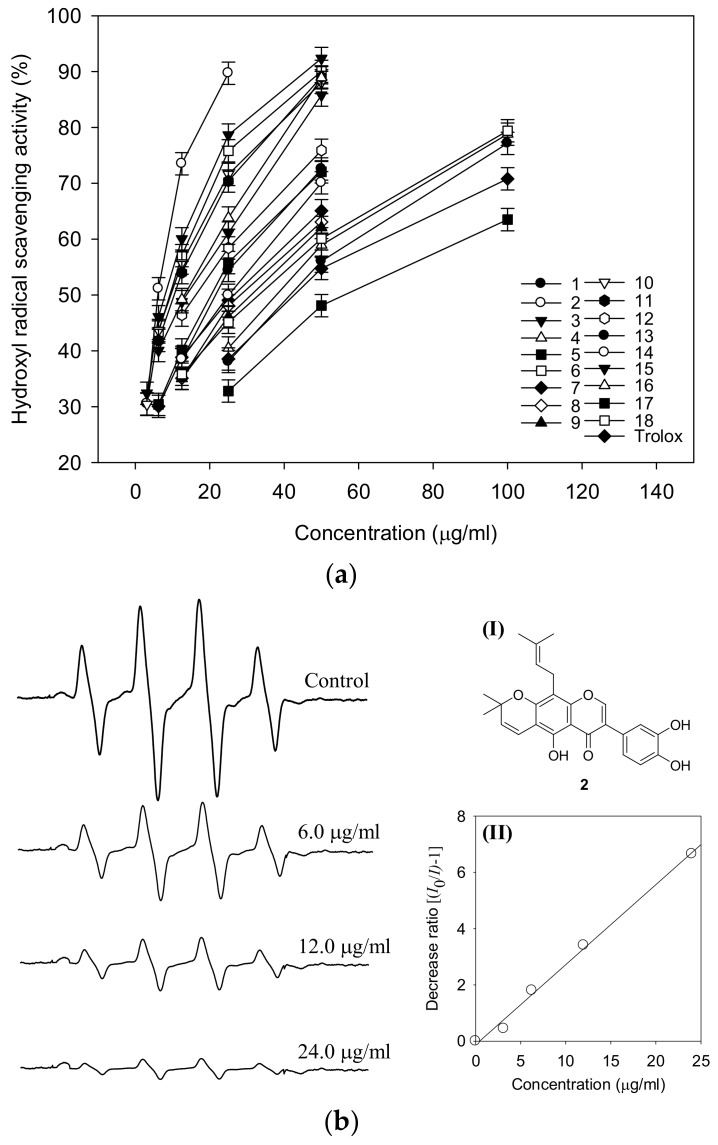
(**a**) Hydroxyl radical scavenging activity of the identified compounds **1**–**18** and trolox; (**b**) ESR spectra of hydroxyl radical according to dose of compound **2** (0, 6.0, 12.0 and 24.0 μg/mL); inset (I): chemical structure of **2** showing the most active hydroxyl radical scavenging effect; inset (II): plot of ESR signal ratio versus concentration of **2**.

The hydroxyl radical assay was conducted for the isolated compounds (**1**–**18**). The hydroxyl radical is a major reactive oxygen species capable of modifying most of the molecules in the living cell. These radicals cause strand damage in DNA and lipid peroxidation of unsaturated fatty acids [15]. Hydroxyl radicals were generated from Fenton’s reaction (Fe^2+^/H_2_O_2_ system) and trapped by DMPO to form a spin adduct which was detected by an ESR spectrometer. The DMPO-OH adduct showed the typical 1:2:2:1 ESR signal (Figure 4). All compounds showed significant hydroxyl radical scavenging effects with ranging from 5.4 to 53 μg/mL. The most potent antioxidants were found to be compounds **2**, **3**, **6**, **10**, **11**, **15** and **16**, with 5.4, 7.5, 9.3, 9.7, 10.5, 14.5 and 14.2 μg/mL, respectively (Figure 4a). ESR signals were reduced by compound **2** in a dose-dependent manner. The radical was completely quenched by 24.0 μg/mL of compound **2** (Figure 4b). The scavenging abilities of the selected compounds **2**, **3**, **4** and **5** were also tested at a different concentration (Table 3), in which compounds **2** and **3**, being the most potent ones with IC_50_ values of 5.4 and 7.5 μg/mL, were compared with compounds **4** and **5,** which showed lower potencies—IC_50_ of 40.3 and 23.2 μg/mL. All tested compounds showed a dose-dependent tendency and better activity than trolox.

### 2.6. Protective Effects of pBR322 Plasmid DNA Damage

**Figure 5:**
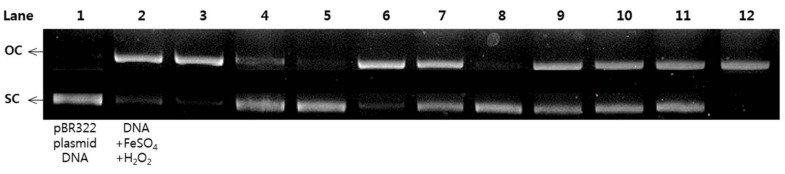
Protective effect of metabolites (**1**–**9**) against pBR322 DNA cleavage induced by hydroxyl radical oxidative injury. Lane 1: pBR322 plasmid DNA control; lane 2: pBR322 DNA + 1 mM FeSO_4_ + 30% H_2_O_2_; lane 3: compound **1**, 60 μM; lane 4: compound **2**, 60 μM; lane 5: compound **3**, 60 μM; lane 6: compound **4**, 60 μM; lane 7: compound **5**, 60 μM; compound **6**, 60 μM; lane 9: compound **7**, 60 μM; lane 10: compound **8**, 60 μM; lane 11: compound **9**, 60 μM; lane 12: Trolox (positive control), 60 μM.

**Figure 6:**
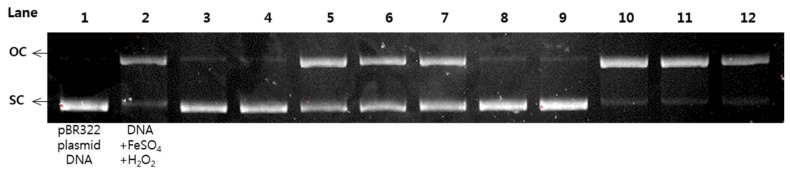
Protective effects of metabolites (**10**–**18**) against pBR322 DNA cleavage induced by hydroxyl radical oxidative injury. Lane 1: pBR322 plasmid DNA control; lane 2: pBR322 DNA + 1 mM FeSO_4_ + 30% H_2_O_2_; lane 3: compound **10**, 60 μM; lane 4: compound **11**, 60 μM; lane 5: compound **12**, 60 μM; lane 6: compound **13**, 60 μM; lane 7: compound **14**, 60 μM; lane 8: compound **15**, 60 μM; lane 9: compound **16**, 60 μM; lane 10: compound **17**, 60 μM; lane 11: compound **18**, 60 μM; lane 12: Trolox (positive control), 60 μM.

**Figure 7:**
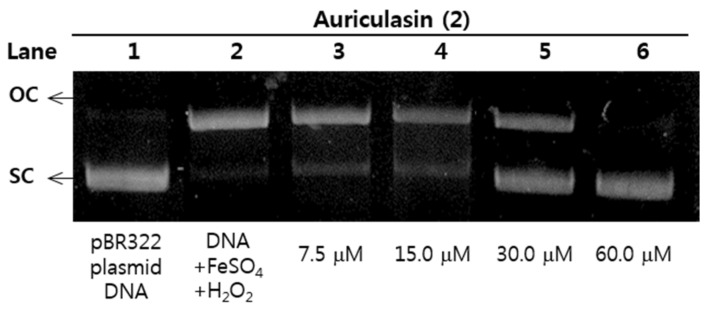
Dose-dependent protective effect of auriculasin (**2**) against pBR322 DNA cleavage induced by hydroxyl radical oxidative injury. Lane 1: pBR322 plasmid DNA control; lane 2: pBR322 DNA + 1 mM FeSO_4_ + 30% H_2_O_2_; lane 3: compound **2**, 7.5 μM; lane 4: compound **2**, 15 μM; lane 5: compound **2**, 30 μM; lane 6: compound **2**, 60 μM.

Reactive oxygen species (ROS) have been known to cause DNA damage in many biological macromolecules which may ultimately lead to carcinogenesis [17]. The damage of plasmid DNA causes the conversion of supercoiled circular DNA (scDNA) to an open circular form (ocDNA) and further, a linear double stranded DNA molecule (lnDNA) [16]. All the identified phenolic compounds (**1**–**18**) in Figure 1 were tested for their protective effects on pBR322 plasmid DNA damage caused by hydroxyl radicals generated from Fenton’s reaction (Fe^2+^ and H_2_O_2_). Figure 5 and Figure 6 shows the protective potentials of all the identified compounds (**1**–**18**) on pBR322 plasmid DNA at a concentration of 60 μM. DNA derived from pBR322 plasmid showed three bands in agarose gels. The faster moving band was the native form of scDNA, while the slower moving bands correspond to ocDNA and lnDNA, which are formed due to the cleavage of the supercoiled circular DNA by the hydroxyl radicals generated from Fenton’s reaction. Figure 5 and Figure 6 showed the inhibition effects of eighteen antioxidants (**1**–**18**) against DNA scission. Inhibitory efficacies varied between 6.9 and 90.9% according to chemical structure (Supplementary Materials). Compounds **2**, **3**, **6**, **10**, **11**, **15** and **16** showed significant protective effects on DNA damage with over 80% efficacy. The most effective antioxidant was found to be auriculasin **2** with 90.9% DNA damage protection, which was substantially higher than trolox (19.1%). It was followed by 6,8-diprenylorobol (**3**) (88.5%), flemiphilippinin A (**6**) (84.5%) and fleminchalcone A (**10**) (84.4%). The most active compound–auriculasin (**2**)—protected the plasmid DNA from damage in a dose-dependent manner, with 10.9% at 60 μM, 27.3% at 30 μM, 52.4%% at 15 μM and 74.5% at 7.5 μM (Figure 7). The protective potential of the identified compounds against the plasmid DNA damage was found to have a significant correlation (*R*^2^ = 0.9525) with the hydroxyl radical scavenging capacity of the compounds (Figure 3b).

## 3. Materials and Methods

### 3.1. Reagents and Materials

2,2-Diphenyl-1-picrylhydrazyl (DPPH), 6-hydroxyl-2,5,7,8-tetramethylchroman-2-carboxylic acid (Trolox), 5,5-dimethyl-1-pyrroline *N*-oxide (DMPO), riboflavin, hydrogen peroxide (30%, H_2_O_2_), dimethyl sulfoxide (DMSO), 2,2′-azobis(2-methylpropionamidine) dihydrochloride (AAPH), fluorescein, Folin–-Ciocalteu’s phenol reagent, quercetin, gallic acid, chloroform-*d*, acetone-*d*_6_ and methanol-*d* were purchased from Sigma Aldrich (St. Louis, MO, USA). pBR322 plasmid DNA was purchased by Thermo Fisher Scientific (Waltham, MA, USA). Silica gel (230–400 mesh), NP F254 and RP-18 F254 TLC plates were obtained from Merck (Darmstadt, Germany). Spherical C18 100 Å reversed phase silica gel (particle size: 20–40 μm) was obtained from SILICYCLE (Ville de Québec, QC, Canada). Sodium hydroxide, ethylenediamine tetraacetic acid, disodium dehydrate (EDTA), iron (II) sulfate heptahydrate (FeSO_4_), sodium nitrite (NaNO_2_), sodium carbonate anhydrous (Na_2_CO_3_), aluminium chloride (AlCl_3_), methanol, acetone, ethylacetate, chloroform and n-hexane were purchased from Duksan Co. (Gyenggi, Korea).

### 3.2. Equipment

1D and 2D NMR spectra (^1^H, ^13^C, DEPT-90, DEPT-135, COSY, HMQC and HMBC) were recorded on a Bruker (AM 500 MHz) spectrometer. Electron ionization (EI) and EI-high resolution (HR) mass spectra were obtained on a JEOL JMS-700 instrument (JEOL Ltd., Akishima, Japan). The MPLC analysis was performed using a LC-Forte/R 100 (YMC Co., Ltd., Kyoto, Japan) system equipped with a low-pressure gradient pump, column compartment and three-channel UV detector. Enzymatic assays were carried out on a SpectraMaxM3Multi-Mode Microplate Reader (Molecular Devices, San Jose, CA, USA). DPPH, hydroxyl and superoxide radical were measured using a JEOL JES-TE300 ESR spectrometer (JEOL Ltd., Akishima, Japan).

### 3.3. Extraction and Isolation of F. philippinensis

The dried root barks of *F. philippinensis* Merr. et Rolfe (0.3 kg) were extracted with EtOH (10 L × 2) to give a crude extract (35 g), which was fractionated by silica gel column eluting with hexane, ethylacetate and methanol. Evaporation of ethylacetate fraction gave 12 g of polyphenol rich fraction. This ethylacetate extract (2 g × 6) was fractionated by MPLC over reversed phase silica gel (250 g, 40 μm) eluting with H_2_O/MeOH gradient (0–100%, 25 mL/min) to afford 25 fractions; this procedure was repeated several times before final purification. Sub-fractions 6–8 (135 mg) were further purified by repeated MPLC with ODS-C18 column (100 g, 25 μm) to give compounds **1** (5 mg), **3** (14 mg), **17** (5 mg) and **18** (6 mg). Repeated MPLC of sub-fraction 10 (260 mg) yielded compounds **2** (17 mg), **5** (7 mg), **7** (13 mg) and **9** (6 mg). Fractions 12–19 (4.7 g, 1.2 g × 4) were purified over ODS-C18 column (200 g, 25 μm) eluting with H_2_O/MeOH gradient (0–100%, 15 mL/min) to give 10 sub-fractions. Sub-fractions 3–5 (240 mg) were further purified by repeated MPLC with ODS-C18 column (200 g, 25 μm) to give compounds **4** (17 mg), **6** (23 mg), **16** (19 mg) and a mixture of **10** and **12**, which were separated by Sephadex LH-20 eluting with 90% MeOH to afford **10** (13 mg) and **12** (9 mg). Repeated MPLC of sub-fractions 6–9 (670 mg) on ODS-C18 column (100 g, 25 μm) gave compounds **8** (7 mg), **14** (10 mg) and **15** (12 mg), and a mixture of **11** and **13**, which were purified by Sephadex LH-20 eluting with 90% MeOH to afford compounds **11** (11 mg) and **13** (16 mg). All isolated compounds were identified on the basis of spectroscopic data and comparison of previous studies [23,24,25,26]. The spectroscopic data are presented in the Supplementary Materials.

### 3.4. Total Phenolic Content (TPC)

The total phenolic content was measured with the Folin–Ciocalteu assay with slight modifications [30]. First, a standard curve was calibrated using diluted gallic acid with DMSO (0–500 μg/mL). Then, 0, 20, 50 and 80% ethanol extracts of root bark of *F. philippinensis* (40 μL) were mixed with 40 μL of Folin–Ciocalteu’s phenol reagent and 360 μL of distilled water (DW) in the 1.5 mL eppendorf tube. The mixture was incubated for 5 min at room temperature. After incubation, 400 μL of sodium carbonate (7% *w*/*v*) solution and 160 μL of DW were added to the reaction mixture and then incubated for 90 min at room temperature. The mixture in the eppendorf tube was centrifuged at 13,000 rpm for 5 min, and 200 μL of supernatant was put into the 96-well plate, and the absorbance at 750 nm was recorded using Spectramax M3 against the blank of DMSO. The total phenolic content was indicated as mg gallic acid equivalents (GAE)/100 g sample.

### 3.5. Total Flavonoid Content (TFC)

The total flavonoid content was measured by the method shown in reference [31] with some modifications. First, a standard curve was calibrated using diluted quercetin with DMSO (0–500 μg/mL). Then, 0, 20, 50 and 80% ethanol extracts of root bark of *F. philippinensis* (100 μL) were mixed with 400 μL of DW and 30 μL of sodium nitrite (5% *w*/*v*) solution in the 1.5 mL eppendorf tube. The mixture was incubated for 5 min at room temperature, and then 30 μL of aluminium chloride (10% *w*/*v*) solution was added. The mixture was incubated for 5 min at room temperature. After that, 200 μL of 1 M sodium hydroxide and 240 μL of DW were added to the reaction mixture and were vortexed for 1 min. A quantity of 200 μL of the mixture in the eppendorf tube was put into the 96-well plate, and the absorbance at 415 nm was recorded using Spectramax M3. The total flavonoid content was indicated as mg quercetin equivalents (QE)/100 g sample.

### 3.6. Electron Spin Resonance Spectroscopy (ESR)

DPPH, hydroxyl and superoxide radicals were detected using a JEOL JES-TE300 ESR spectrometer equipped with X-Band microwave unit at 100 kHz of modulation frequency and TE102 cavity mode. The magnetic field and frequency were calibrated using a Jeol ES-FC5 precision gauss meter and HP 5350B frequency counter, respectively. The temperature was kept 20 °C using a cooling water circulator. Spectral acquisition, manipulations and simulations were carried out with the software, ES-IPRITS-TE. All compounds (**1**–**18**) were diluted at different concentrations (0, 50, 100, 200, 400, 800 and 1600 mg/mL) using DMSO and were measured in quartz flat tubes. This ESR condition was used for DPPH, hydroxyl and superoxide radical measurement. 

#### 3.6.1. DPPH Radical Scavenging Assay by ESR

DPPH radical scavenging activity was described by the method used in reference [32] with some modifications. Ten μL of each sample was mixed with 170 μL of 0.15 mM DPPH solution in ethanol. The measurement conditions were as follows: magnetic field, 339.5 mT; microwave frequency, 9.42 GHz; power, 5 mW; sweep time, 2 min; modulation, 100 kHz; amplitude, 1 × 100; time constant, 0.1 s. The DPPH radical scavenging rate of compounds was calculated using the following equation, where *I*_0_ and *I* are the ESR intensities in the absence and presence of a compound, respectively. The activity was expressed as the IC_50_ value, that is, 50% of radical scavenging activity. Trolox was used as a positive control.

Radical scavenging activity (%) = [(*I*_0_ − *I*)/*I*_0_] × 100 (1)

#### 3.6.2. Hydroxyl Radical Scavenging Assay by ESR

Hydroxyl radical scavenging activity was described by the method shown in reference [33] with some modifications. Hydroxyl radical was generated by the Fenton reaction. Briefly, 20 μL of sample was mixed with 50 μL of 0.3 M DMPO in 10 mM phosphate buffer (pH 7.4), 50 μL of 30% H_2_O_2_ and 30 μL of 10 mM fresh FeSO_4._ The measurement conditions were as follows: magnetic field, 339.5 mT; microwave frequency, 9.42 GHz; power, 1 mW; sweep time, 2 min; modulation, 100 kHz; amplitude, 1 × 100; time constant, 0.1 s. The hydroxyl radical scavenging activities of compounds were calculated using the same equation as that used in the DPPH method. The activity was expressed as the IC_50_ value, that is, 50% of radical scavenging activity. 

#### 3.6.3. Superoxide Radical Scavenging Assay by ESR

Superoxide radical scavenging activity was evaluated following a previously described protocol [34]. Superoxide radicals were generated by UV irradiation of the riboflavin/EDTA system in the absence and presence of compounds. Briefly, 20 μL of sample was mixed with 30 μL of 0.3 mM riboflavin in 10 mM phosphate buffer (pH 7.4), 50 μL of EDTA in 10 mM phosphate buffer (pH 7.4) and 100 μL of 0.3 M DMPO in 10 mM phosphate buffer (pH 7.4). Finally, the mixture was measured immediately after irradiating under a UV lamp with 365 nm wavelengths for 60 s. The measurement conditions were as follows: magnetic field, 339.5 mT; microwave frequency, 9.42 GHz; power, 1 mW; sweep time, 2 min; modulation, 100 kHz; amplitude, 5 × 100; time constant, 0.1 s. The superoxide radical scavenging activity of compounds were calculated using the same equation as that used in the DPPH method. The activity was expressed as the IC_50_ value, that is, 50% of radical scavenging activity. 

### 3.7. ORAC Scavenging Activity Assay

The ORAC assay was used as described in a previous study [35] with slight modifications. The ORAC assay was performed on a 96-well microtiter clear bottom black plate (SPL Life Sciences, Gyenggi, Korea). First, a standard curve was calibrated using diluted trolox with DMSO (0–200 μg/mL). Then, 25 μL of Trolox or a test sample (**1**–**18**) were mixed with 150 μL of 100 mM fluorescein in each well. The mixture was incubated at 37 °C for 30 min. After incubation, 25 μL of 50 mM AAPH, free radical initiator, was added to the wells where each reaction mixture was present. The reaction mixture was recorded using Spectramax M3 at 37 °C every 2 min for 60 min. The excitation and emission wavelength conditions were 480 and 520 nm, respectively. The calculation of the ORAC value was determined from the net AUC the area under the curve (AUC) using the following equation, where RFU*x* and RFU_0_ are the relative fluorescence values of a time point and zero, respectively. The ORAC values were presented as Trolox equivalents.

AUC = 1 + RFU_1_/RFU_0_ + RFU_2_/RFU_0_ + … + RFU_59_/RFU_0_ + RFU_60_/RFU_0_ (2)

Net AUC = AUC sample − AUC negative control(3)

### 3.8. DNA Damage Protective Effect Assay

The hydroxyl radical-induced DNA damaged assay was conducted according to a modified method of reference [36]. Briefly, 1 μL of pBR322 plasmid DNA (0.35 μg/μL) in 9 μL of 50 mM phosphate buffer (pH 7.4), 2 μL of 1 mM FeSO_4_, 5 μL of samples (**1**–**18**) and 3 μL of 30% H_2_O_2_ was incubated at 37 °C for 30 min in dark conditions. After incubation, 5 μL of the reaction mixture and 1 μL of 6× DNA loading buffer (Enzynomics, Daejeon, Korea) were mixed and loaded onto a 0.8% agarose gel containing 1× RedSafe, DNA staining solution, (Intron biotechnology, Gyenggi, Korea) in TAE buffer (40 mM Tris-acetate and 1 mM EDTA) and electrophoresed for 30 min at 85 V. After electrophoresis, the DNA in the gel was visualized and photographed using the Gel Doc XR+ system equipped with a universal hood, UV transilluminator and camera. Analysis of DNA band intensity and imaging in agarose gel were carried out Image Lab software. Trolox (60 μM) was used as a positive control. Inhibition of DNA damage (%) was calculated using Equations (4) [37]:

DNA damage (%) = ocDNA band intensity/pBR322 DNA band intensity × 100(4)

### 3.9. Statistical Analysis

All experiments were conducted in triplicate. The results were implemented to variance analysis using Sigma Plot (version 10.0, Systat Software, Inc., San Jose, CA, USA). Differences were considered significant at *p* < 0.05.

## 4. Conclusions

About eighteen phenolic compounds (**1**–**18**) were isolated and identified from *F. philippinensis* Merr. et Rolfe, which has been used against oxidative stress-related diseases, including rheumatism. Antioxidant analyses of all the isolated compounds (**1**–**18**) were carried out using four different kinds of radical sources. The radical scavenging properties of these compounds were studied which provided substantial data about the antioxidant potential of the target plant. This study, for the first time, confirmed that seven candidate antioxidants, namely, compounds **2**, **3**, **6**, **10**, **11**, **15** and **16** of *F. philippinensis*, can exhibit significant protection against plasmid DNA damage. The DNA damage protection effects of the identified compounds had a high correlation with hydroxyl radical scavenging activity. Moreover, further in vivo studies are necessary to clearly establish the beneficial effects of these metabolites in an animal model.

## Figures and Tables

**Table 1:** Total phenols, flavonoids and DPPH scavenging activity in *F. philippinensis* extracts.

Extractant	Total Phenolics	Total Flavonoids	DPPH
	(GAE mg/100 g)	(QE mg/100 g)	(% at 100 μg/mL)
0% EtOH	3300 ± 104.5	285 ± 13.5	37.2 ± 1.3
20% EtOH	3411 ± 185.0	892 ± 22.7	49.7 ± 0.8
50% EtOH	4285 ± 98.7	2862 ± 56.4	53.6 ± 0.4
80% EtOH	4947 ± 100.3	3058 ± 10.5	78.0 ± 1.2

**Table 2:** Radical scavenging activities of phenolic compounds **1**–**18**.

Compounds	DPPH ^1^	ORAC ^2^	O_2_^•− 1^	^•^OH ^1^
	(μg/mL)	(μmol TE/g)	(μg/mL)	(μg/mL)
1	37.3 ± 1.5	6.2 ± 0.5	41.2 ± 1.8	43.3 ± 0.1
2	0.9 ± 0.05	11.2 ± 0.6	12.8 ± 1.1	5.4 ± 0.03
3	2.0 ± 0.03	10.5 ± 0.3	15.9 ± 0.7	7.5 ± 0.4
4	21.8 ± 0.5	32.3 ± 1.2	21.5 ± 0.8	40.3 ± 1.2
5	2.8 ± 0.1	15.6 ± 0.7	32.4 ± 0.3	23.2 ± 0.9
6	4.5 ± 0.7	2.5 ± 0.3	40.5 ± 0.2	9.3 ± 0.05
7	50.6 ± 2.4	1.3 ± 0.1	56.4 ± 0.4	28.0 ± 1.4
8	164.5 ± 2.8	2.0 ± 0.4	40.2 ± 0.3	30.9 ± 0.1
9	10.4 ± 0.4	8.1 ± 0.9	21.3 ± 0.6	31.2 ± 0.9
10	85.3 ± 1.6	1.2 ± 0.5	50.5 ± 0.7	9.7 ± 0.3
11	125.4 ± 2.8	0.9 ± 0.03	60.9 ± 1.8	10.5 ± 0.4
12	58.2 ± 0.6	0.3 ± 0.02	92.3 ± 4.4	18.9 ± 1.0
13	96.8 ± 2.0	1.0 ± 0.1	58.8 ± 2.0	23.9 ± 0.8
14	110.3 ± 3.8	1.1 ± 0.2	60.4 ± 1.3	26.6 ± 0.4
15	22.8 ± 0.2	3.3 ± 0.7	30.6 ± 0.5	11.4 ± 0.3
16	4.7 ± 0.9	4.6 ± 0.5	30.1 ± 0.8	12.5 ± 0.7
17	148.2 ± 2.2	0.5 ± 0.03	92.8 ± 2.4	53.3 ± 0.2
18	18.4 ± 3.5	4.4 ± 0.8	34.4 ± 1.6	32.2 ± 0.1
Trolox	3.7 ± 0.06	-	8.8 ± 0.3	21.8 ± 0.7

^1^ The concentrations are the IC_50_ values; ^2^ the concentrations are the ORAC values.

**Table 3:** Hydroxyl radical scavenging activities of the selected compounds **2**–**5** and trolox.

Compounds	^•^OH Scavenging Activity (%)		
	6.0 mg/mL	12.0 mg/mL	24.0 mg/mL
2	73.50	89.71	98.74
3	60.05	78.65	92.35
4	28.40	40.50	58.94
5	40.15	55.88	72.05
Trolox	22.70	38.54	54.75

## Supplementary Materials

The following are available online, Figures S1–S36: Spectroscopic data (^1^H-, ^13^C-NMR, EIMS and HREIMS) of all isolated compounds (**1**–**18**), Figures S37–S42: ESR spectra of DPPH, superoxide and hydroxyl radicals by **1**–**18**, Figures S43–S45: Results of electrophoresis by pBR322 plasmid DNA band intensity using Image Lab software, Tables S1–S2: pBR322 plasmid DNA damage protective effect of **1**–**18**.
