# Supplementary material for: Antioxidant Activities of Phenolic Metabolites from Flemingia philippinensis Merr. et Rolfe and Their Application to DNA Damage Protection
Source: Molecules. 2018 Apr 2;23(4):816. doi: 10.3390/molecules23040816 (PMC6017228; doi:10.3390/molecules23040816)
Supplement: Supplementary file 1 [file molecules-23-00816-s001.pdf]

Supplementary materials

Antioxidant activities of phenolic metabolites from *Flemingia philippinensis* and application to DNA damage protection

Jeong Yoon Kim [1], Yan Wang [2], Yeong Hun Song [1], Zia Uddin [1],

Zuo Peng Li [1], Yeong Jun Ban[1] and Ki Hun Park [1,*]

[1] *Division of Applied Life Science (BK21 plus), IALS, Gyeongsang National University, Jinju 52828, Republic of Korea*

[2] *College of Food and Biological Engineering, Qiqihar University, Quqihar 161006, China*

List of supporting information

Figure S1. ^{1}H-NMR and ^{13}C-NMR spectrums of compound **1**

Figure S2. EIMS and HREIMS data of compound **1**

Figure S3. ^{1}H-NMR and ^{13}C-NMR spectrums of compound **2**

Figure S4. EIMS and HREIMS data of compound **2**

Figure S5. ^{1}H-NMR and ^{13}C-NMR spectrums of compound **3**

Figure S6. EIMS and HREIMS data of compound **3**

Figure S7. ^{1}H-NMR and ^{13}C-NMR spectrums of compound **4**

Figure S8. EIMS and HREIMS data of compound **4**

Figure S9. ^{1}H-NMR and ^{13}C-NMR spectrums of compound **5**

Figure S10. EIMS and HREIMS data of compound **5**

Figure S11. ^{1}H-NMR and ^{13}C-NMR spectrums of compound **6**

Figure S12. EIMS and HREIMS data of compound **6**

Figure S13. ^{1}H-NMR and ^{13}C-NMR spectrums of compound **7**

Figure S14. EIMS and HREIMS data of compound **7**

Figure S15. ^{1}H-NMR and ^{13}C-NMR spectrums of compound **8**

Figure S16. EIMS and HREIMS data of compound **8**

Figure S17. ^{1}H-NMR and ^{13}C-NMR spectrums of compound **9**

Figure S18. EIMS and HREIMS data of compound **9**

Figure S19. ^{1}H-NMR and ^{13}C-NMR spectrums of compound **10**

Figure S20. EIMS and HREIMS data of compound **10**

Figure S21. ^{1}H-NMR and ^{13}C-NMR spectrums of compound **11**

Figure S22. EIMS and HREIMS data of compound **11**

Figure S23. ^{1}H-NMR and ^{13}C-NMR spectrums of compound **12**

Figure S24. EIMS and HREIMS data of compound **12**

Figure S25. ^{1}H-NMR and ^{13}C-NMR spectrums of compound **13**

Figure S26. EIMS and HREIMS data of compound **13**

Figure S27. ^{1}H-NMR and ^{13}C-NMR spectrums of compound **14**

Figure S28. EIMS and HREIMS data of compound **14**

Figure S29. ^{1}H-NMR and ^{13}C-NMR spectrums of compound **15**

Figure S30. EIMS and HREIMS data of compound **15**

Figure S31. ^{1}H-NMR and ^{13}C-NMR spectrums of compound **16**

Figure S32. EIMS and HREIMS data of compound **16**

Figure S33. ^{1}H-NMR and ^{13}C-NMR spectrums of compound **17**

Figure S34. EIMS and HREIMS data of compound **17**

Figure S35. ^{1}H-NMR and ^{13}C-NMR spectrums of compound **18**

Figure S36. EIMS and HREIMS data of compound **18**

Figure S37. ESR spectra of DPPH radical scavenging effect of compounds**1-9**

Figure S38. ESR spectra of DPPH radical scavenging effect of compounds**10-18**

Figure S39. ESR spectra of hydroxyl radical scavenging effect of compounds**1-9**

Figure S40. ESR spectra of hydroxyl radical scavenging effect of compounds**10-18**

Figure S41. ESR spectra of superoxide radical scavenging effect of compounds**1-9**

Figure S42. ESR spectra of superoxide radical scavenging effect of compounds**10-18**

Figure S43-45. Results of electrophoresis by pBR322 plasmid DNA band intensity

Table S1. pBR322 plasmid DNA damage protective effect of **1-18**.

Table S2. Protective effect of pBR322 plasmid DNA by dose dependent of compound **2**

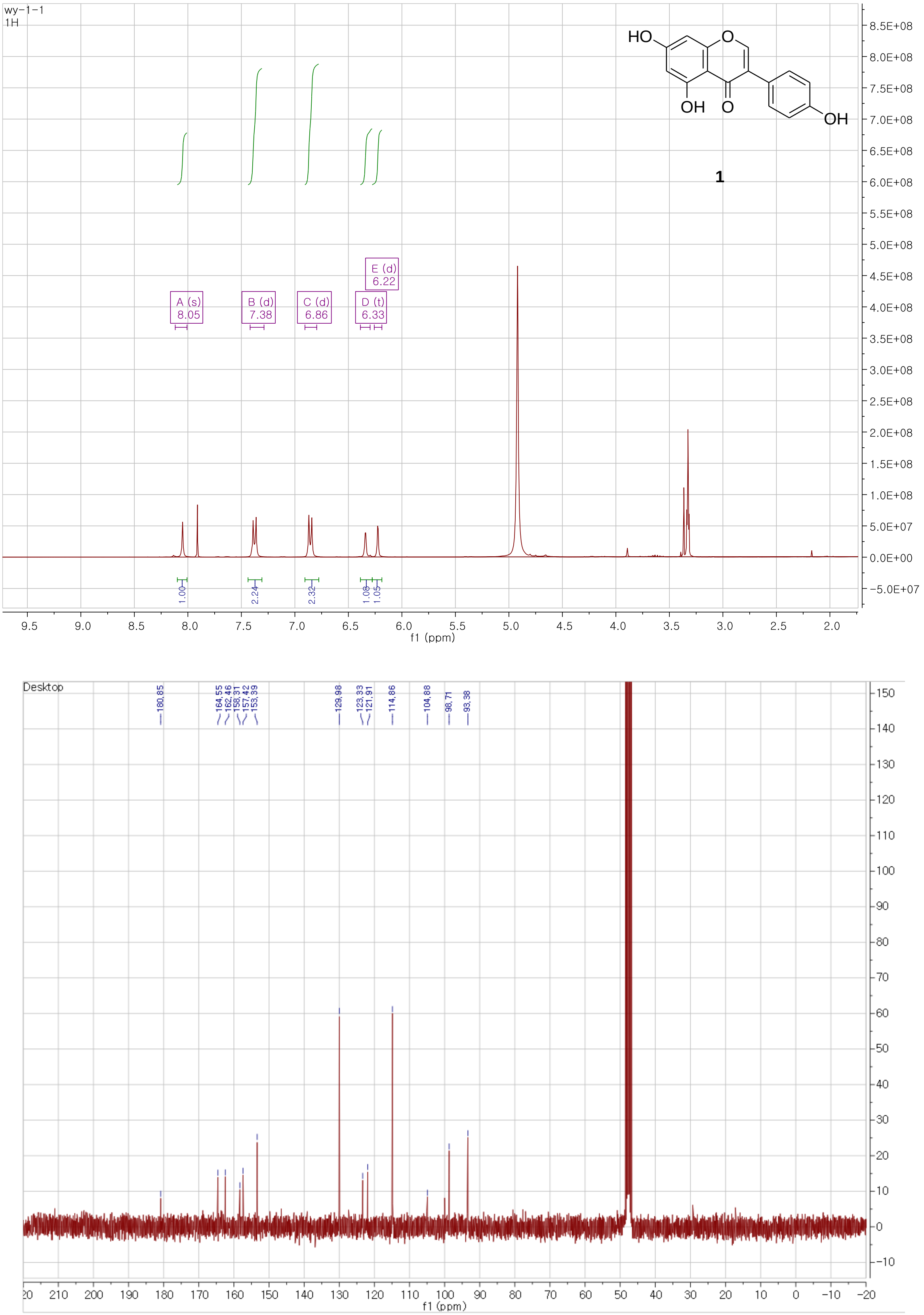

Figure 1. ^{1}H-NMR (500 MHz) and ^{13}C-NMR (125 MHz) spectrums of compound **1**(CD_3OD)

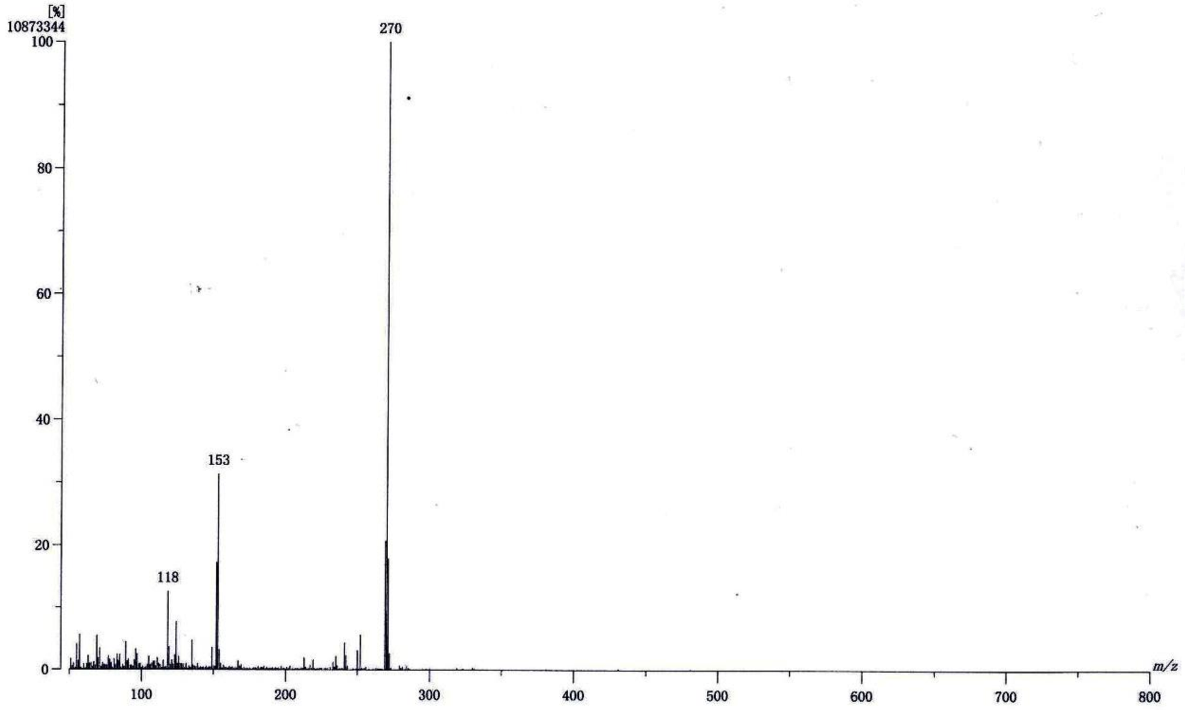

Instrument : MStation
Sample : –
Note : –
Inlet : Direct Ion Mode : EI+
RT : 1.87 min Scan# : 29
Elements : C 24/1, H 49/1, O 10/1
Mass Tolerance : 3mmu
Unsaturation (U.S.) : –0.5 – 30.0

	Observed m/z	Int%	Err[ppm / mmu]	U.S.	Composition
1	270.0531	84.93	+1.0 / +0.3	11.0	C15 H10 O5

Figure 2. EIMS and HREIMS data of compound **1**

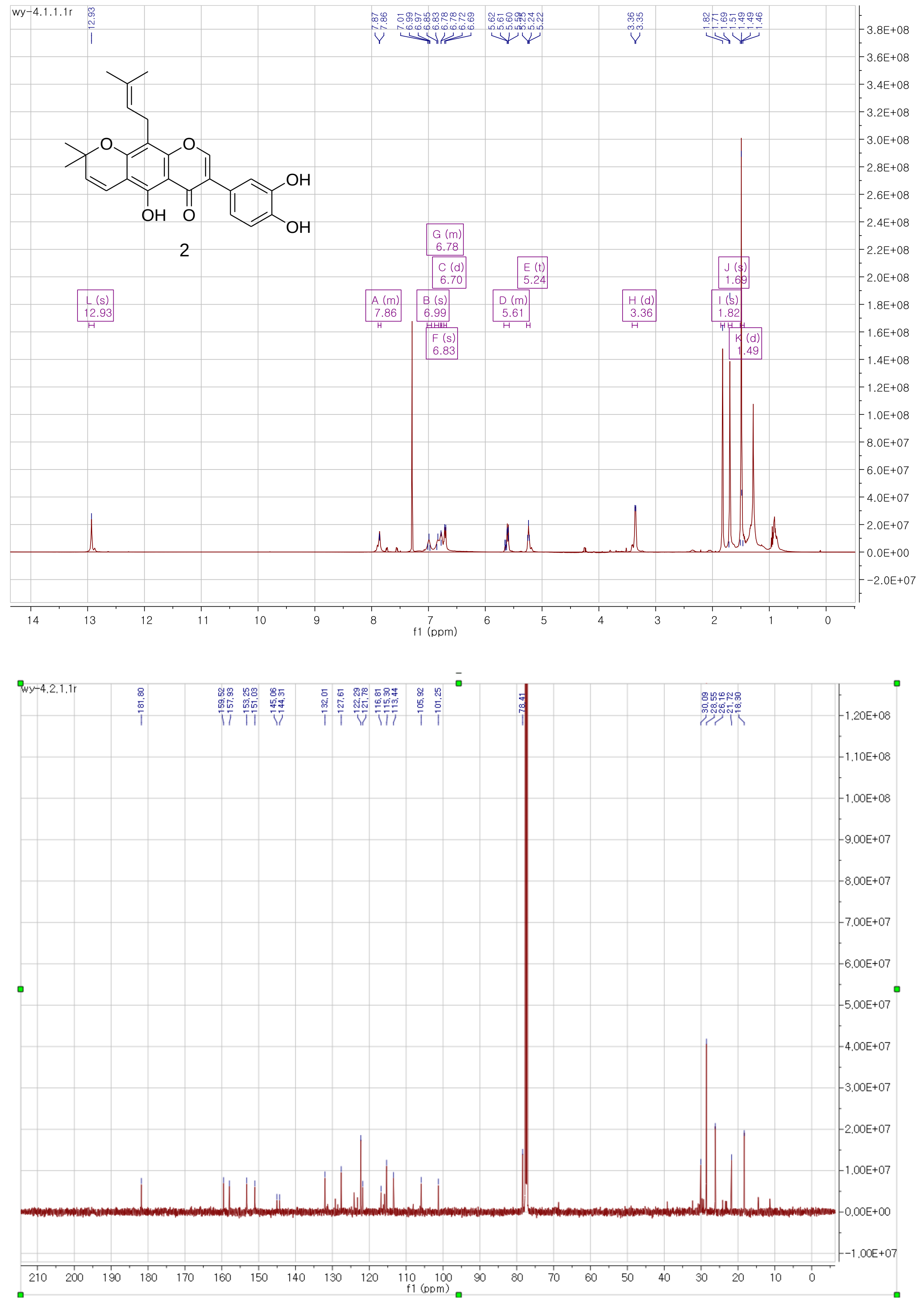

Figure 3. ^{1}H-NMR (500 MHz) and ^{13}C-NMR (125 MHz) spectrums of compound **2** ($CDCl_3$)

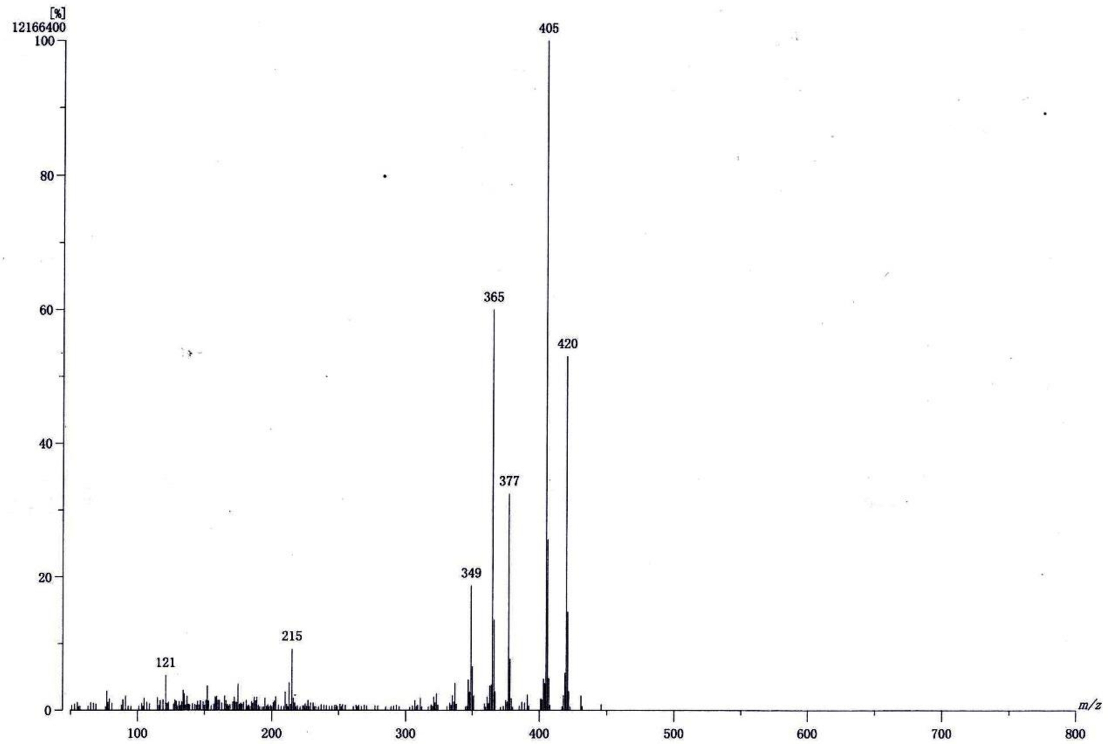

Instrument : MStation
Sample : –
Note : –
Inlet : Direct   Ion Mode : EI+
RT : 2.04 min   Scan# : 62
Elements : C 100/1, H 100/1, O 10/1
Mass Tolerance   : 1000ppm, 3mmu if m/z > 3
Unsaturation (U.S.) : –0.5 – 20.0

	Observed m/z	Int%	Err[ppm / mmu]	U.S.	Composition
1	420.1572	52.63	-0.2 / -0.1	14.0	C25 H24 O6

Figure 4. EIMS and HREIMS data of compound **2**

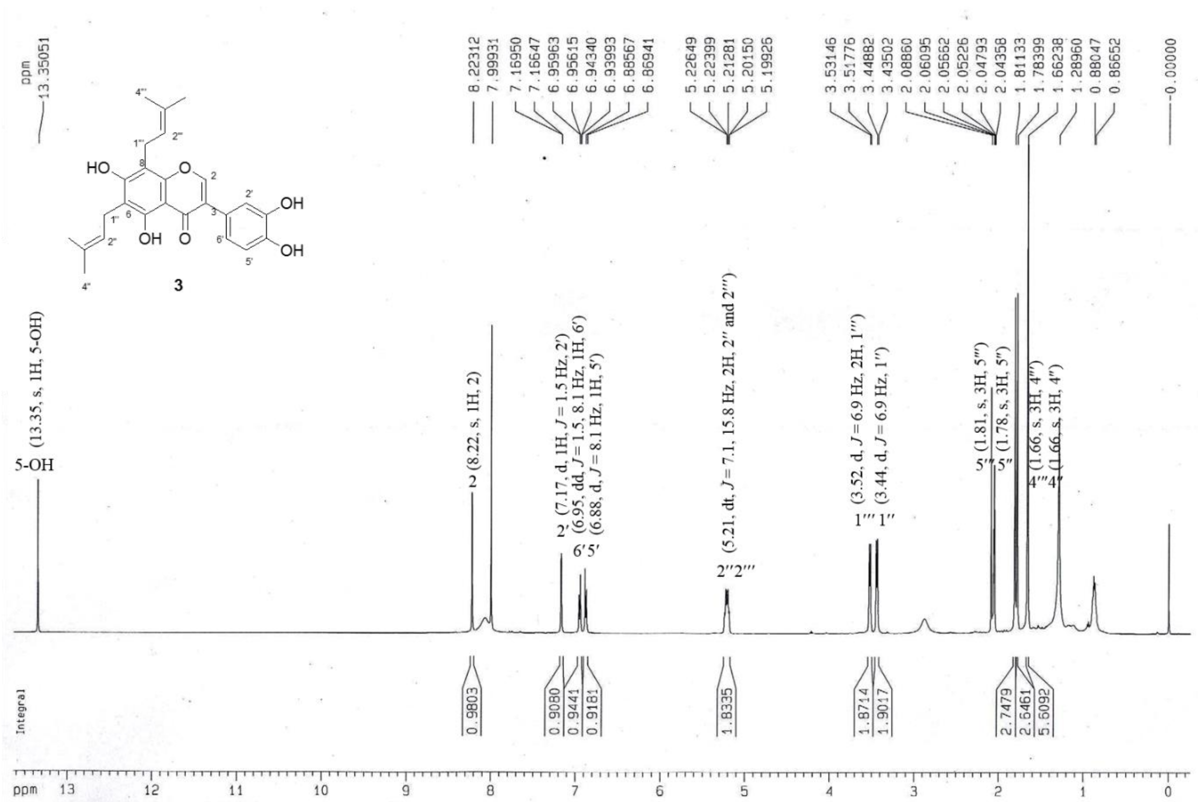

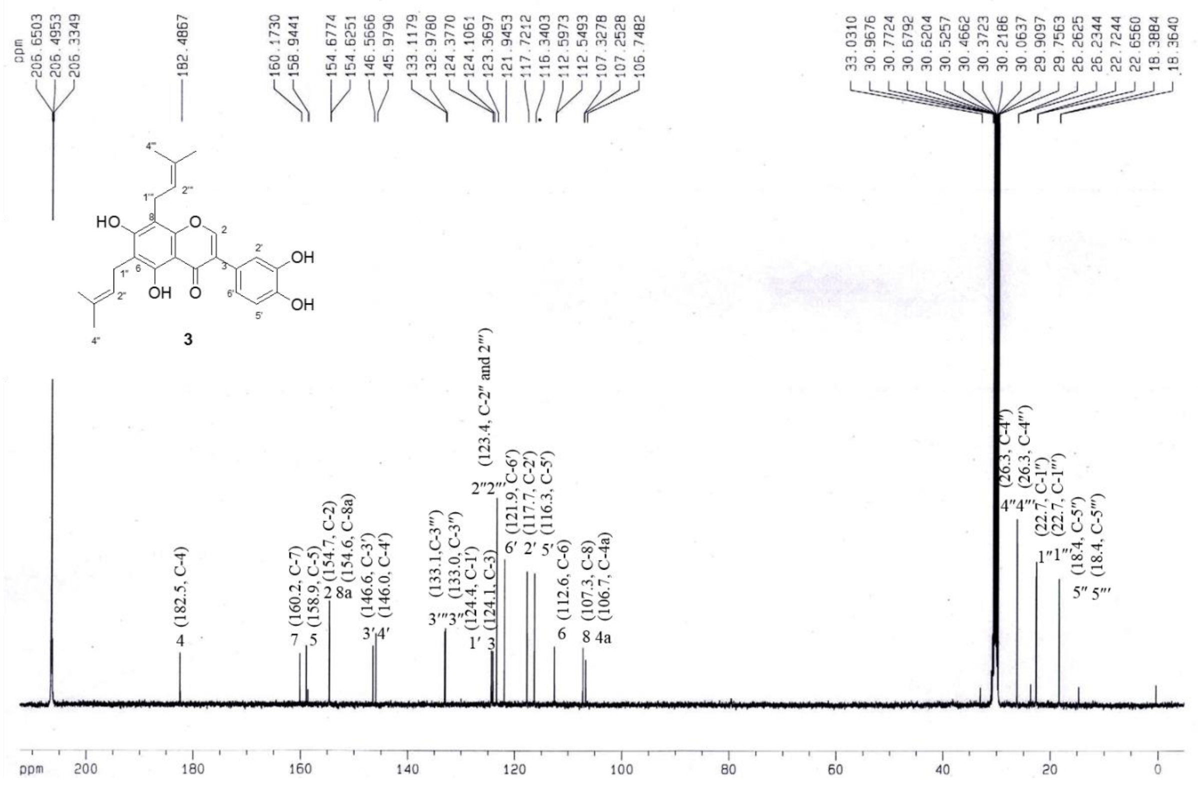

Figure 5. ^{1}H (500 MHz) and ^{13}C-NMR (125 MHz) spectrums of compound **3** (Acetone-d_6)

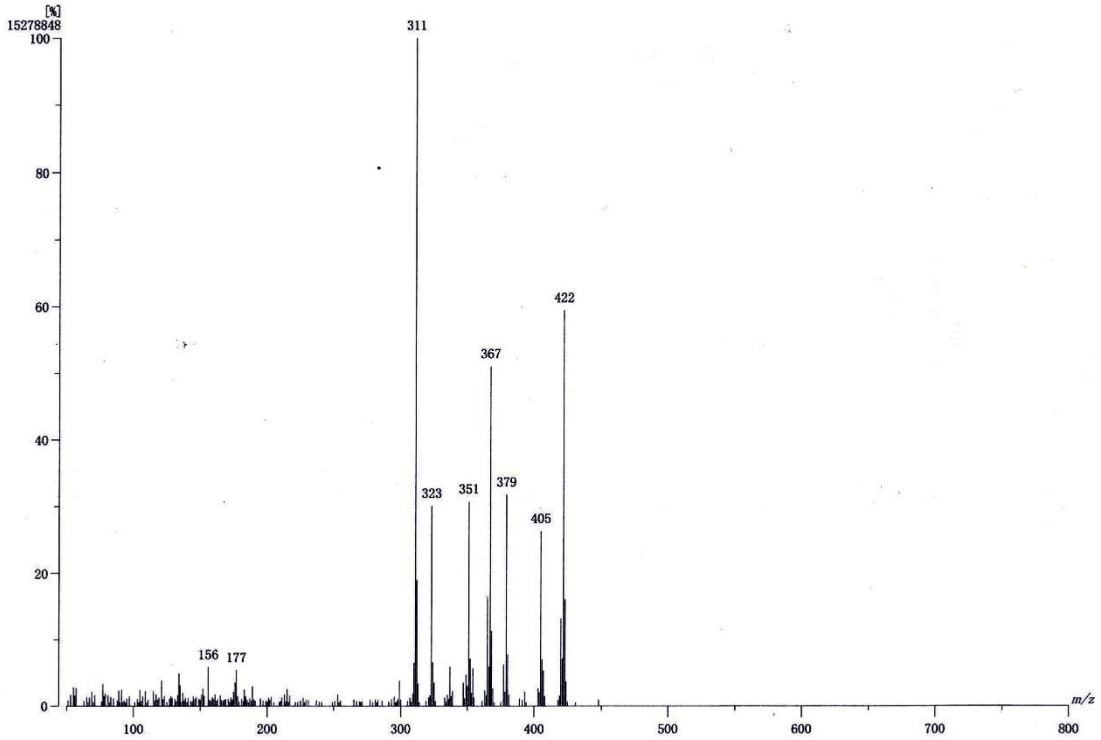

Instrument : MStation
Sample : –
Note : –
Inlet : Direct Ion Mode : EI+
RT : 2.37 min Scan# : 72
Elements : C 100/1, H 100/1, O 10/1
Mass Tolerance : 1000ppm, 3mmu if m/z > 3
Unsaturation (U.S.) : –0.5 – 20.0

	Observed m/z	Int%	Err[ppm / mmu]	U.S.	Composition
1	422.1728	100.00	-0.3 / -0.1	13.0	C25 H26 O6

Figure 6. EIMS and HREIMS data of compound **3**

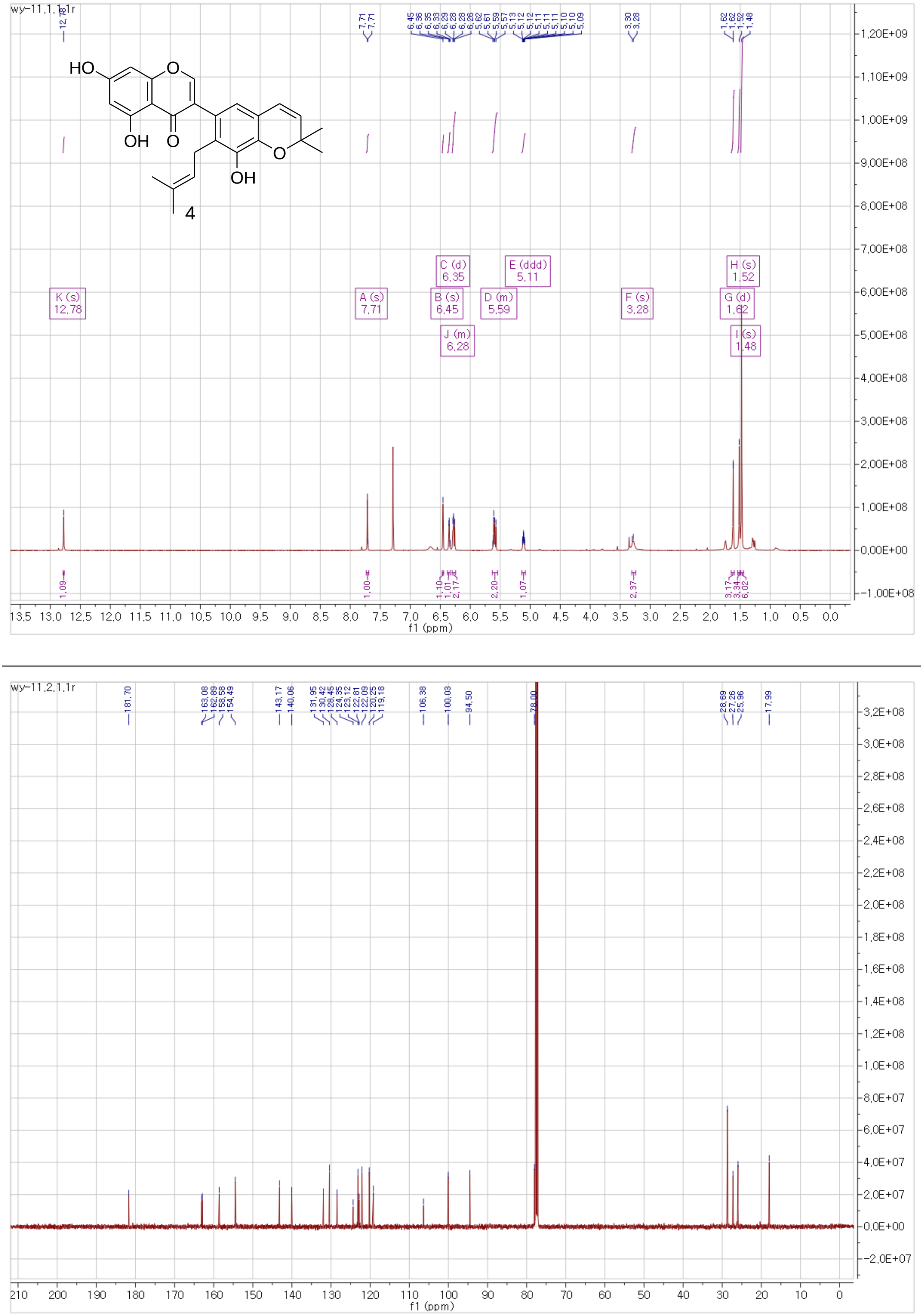

Figure 7. ^{1}H-NMR (500 MHz) and ^{13}C-NMR (125 MHz) spectrums of compound **4** ($CDCl_3$)

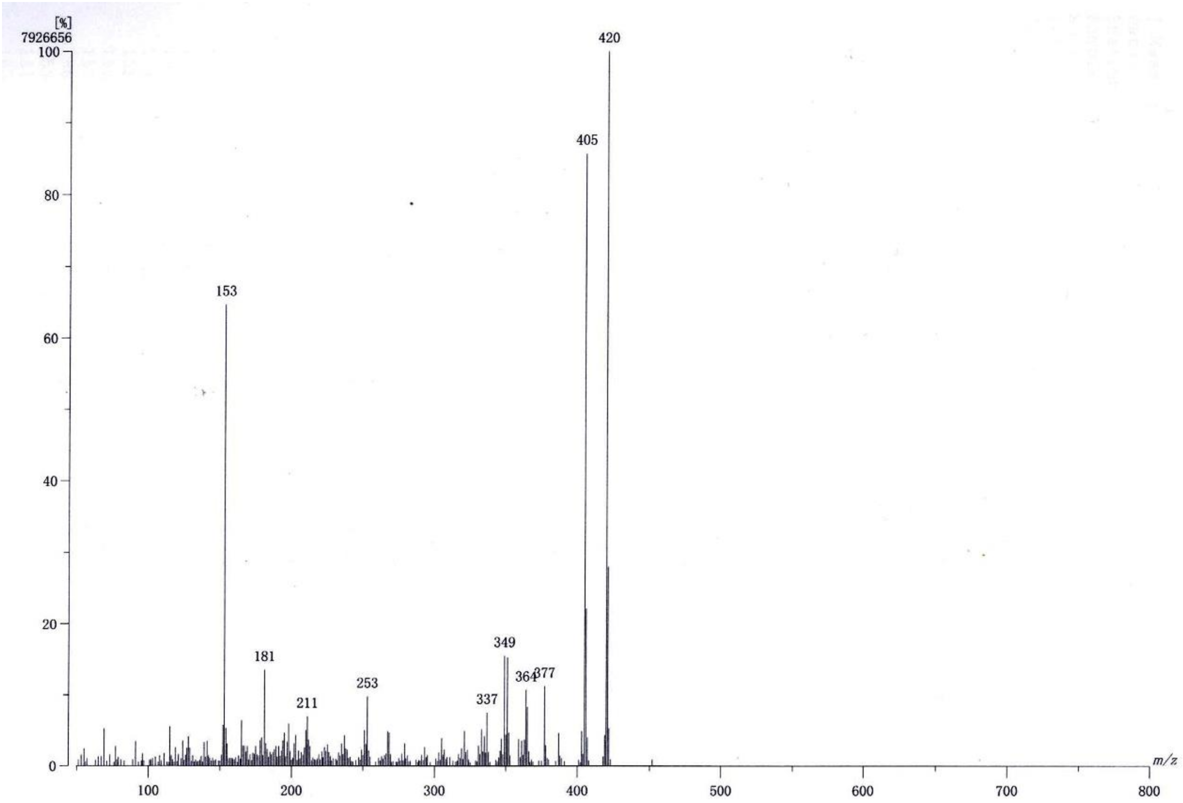

Instrument : MStation
Sample : –
Note : –
Inlet : Direct Ion Mode : EI+
RT : 3.14 min Scan# : 95
Elements : C 100/1, H 100/1, O 10/1
Mass Tolerance : 1000ppm, 3mmu if m/z > 3
Unsaturation (U.S.) : –0.5 – 20.0

	Observed m/z	Int%	Err[ppm / mmu]	U.S.	Composition
1	420.1573	38.36	+0.0 / +0.0	14.0	C25 H24 O6

Figure 8. EIMS and HREIMS data of compound **4**

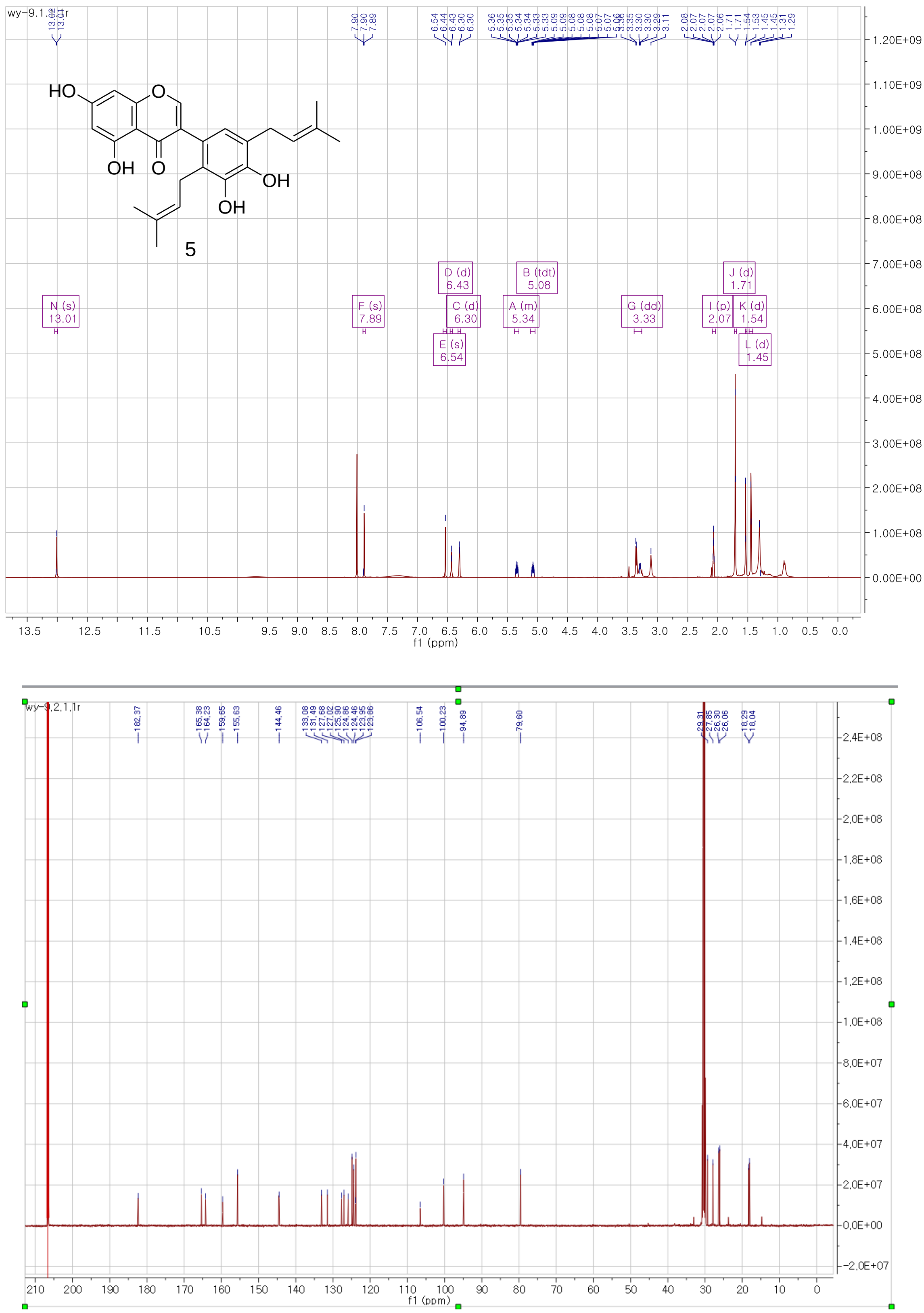

Figure 9. ^{1}H-NMR (500 MHz) and ^{13}C-NMR (125 MHz) spectrums of compound **5** (Acetone-*d*)

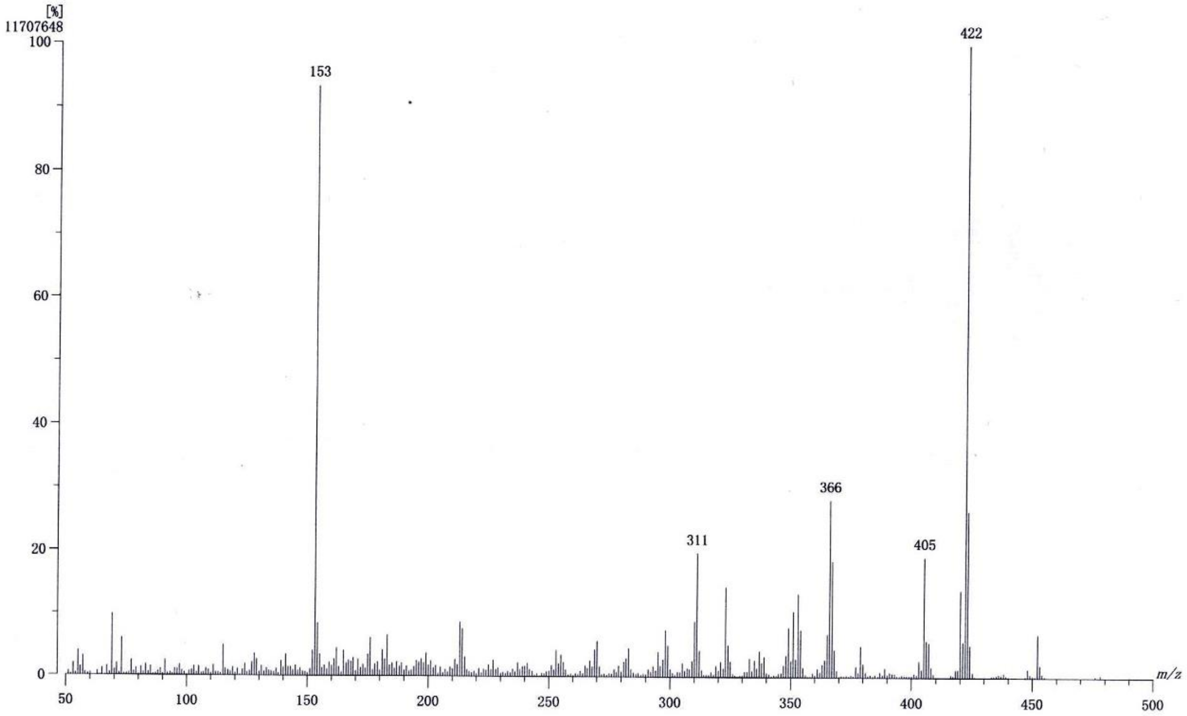

Sample: -
Note : -
Inlet : Direct   Ion Mode : EI+
RT : 2.65 min   Scan#: 54
Elements : C 100/1, H 100/1, O 10/1
Mass Tolerance : 3mmu
Unsaturation (U.S.) : 0.0 - 20.0

Observed m/z	Int%	Err[ppm / mmu]	U.S.	Composition
422.1727	100.0	-0.6 / -0.3	13.0	C 25 H 26 O 6

Figure 10. EIMS and HREIMS data of compound **5**

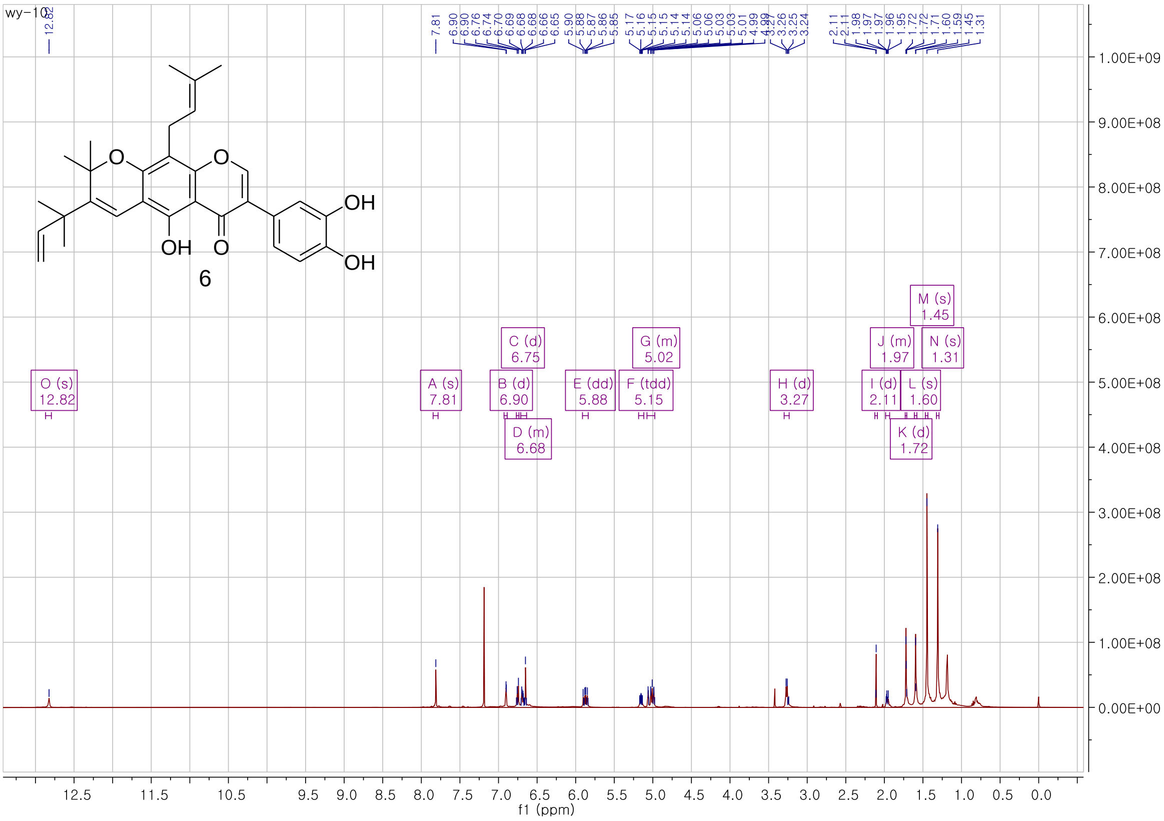

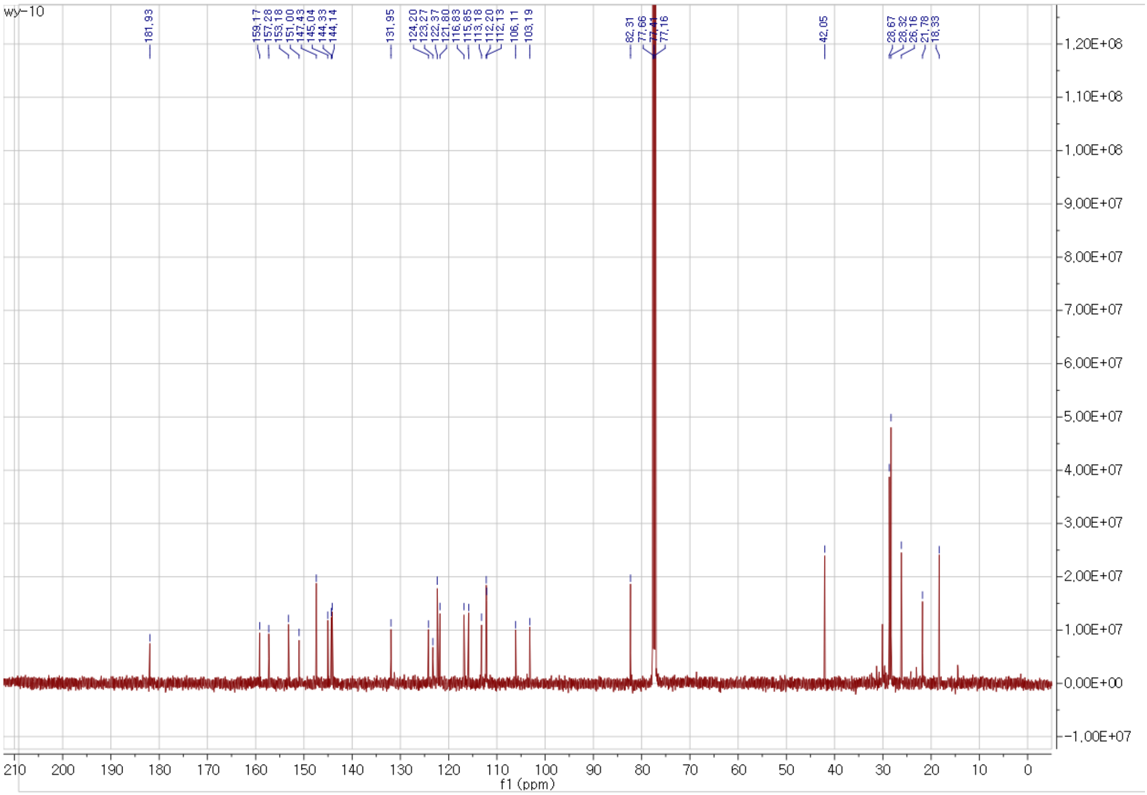

Figure 11. ^{1}H-NMR (500 MHz) and ^{13}C-NMR (125 MHz) spectrums of compound **6** ($CDCl_3$)

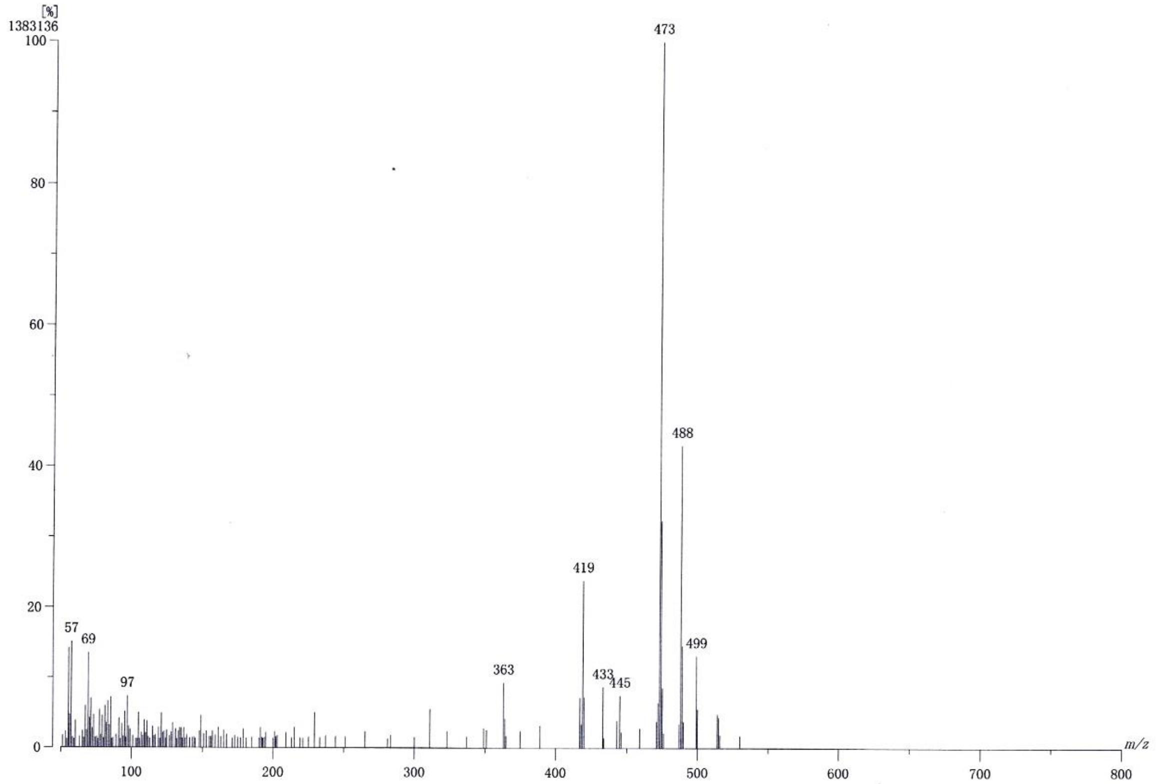

Instrument : MStation
Sample : –
Note : –
Inlet : Direct Ion Mode : EI+
RT : 1.14 min Scan# : 35
Elements : C 100/1, H 100/1, O 10/1
Mass Tolerance : 1000ppm, 3mmu if m/z > 3
Unsaturation (U.S.) : –0.5 – 20.0

	Observed m/z	Int%	Err[ppm / mmu]	U.S.	Composition
1	488.2199	70.20	+0.0 / +0.0	15.0	C30 H32 O6

Figure 12. EIMS and HREIMS data of compound **6**

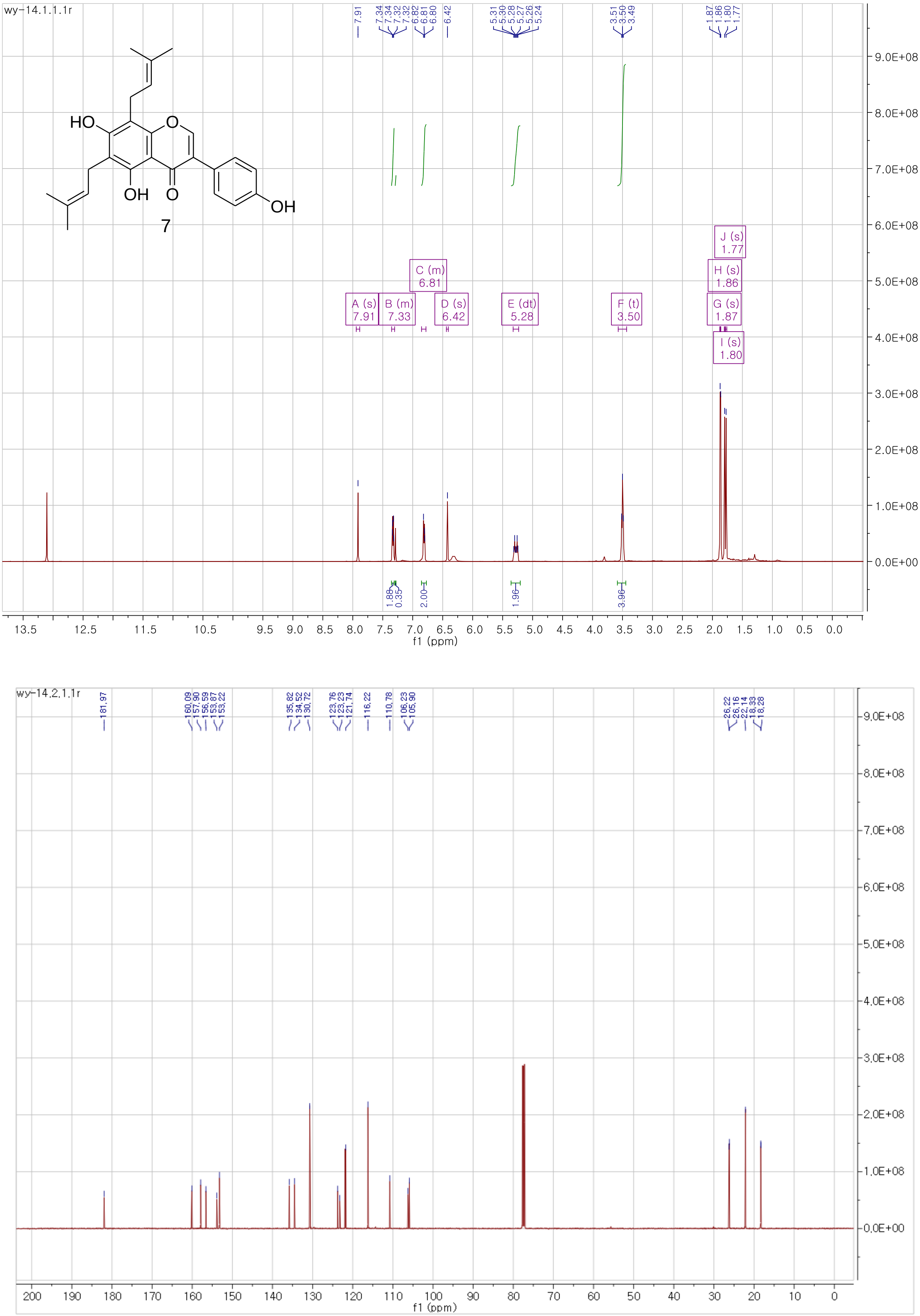

Figure 13. ^{1}H-NMR (500 MHz) and ^{13}C-NMR (125 MHz) spectrums of compound **7** ($CDCl_3$)

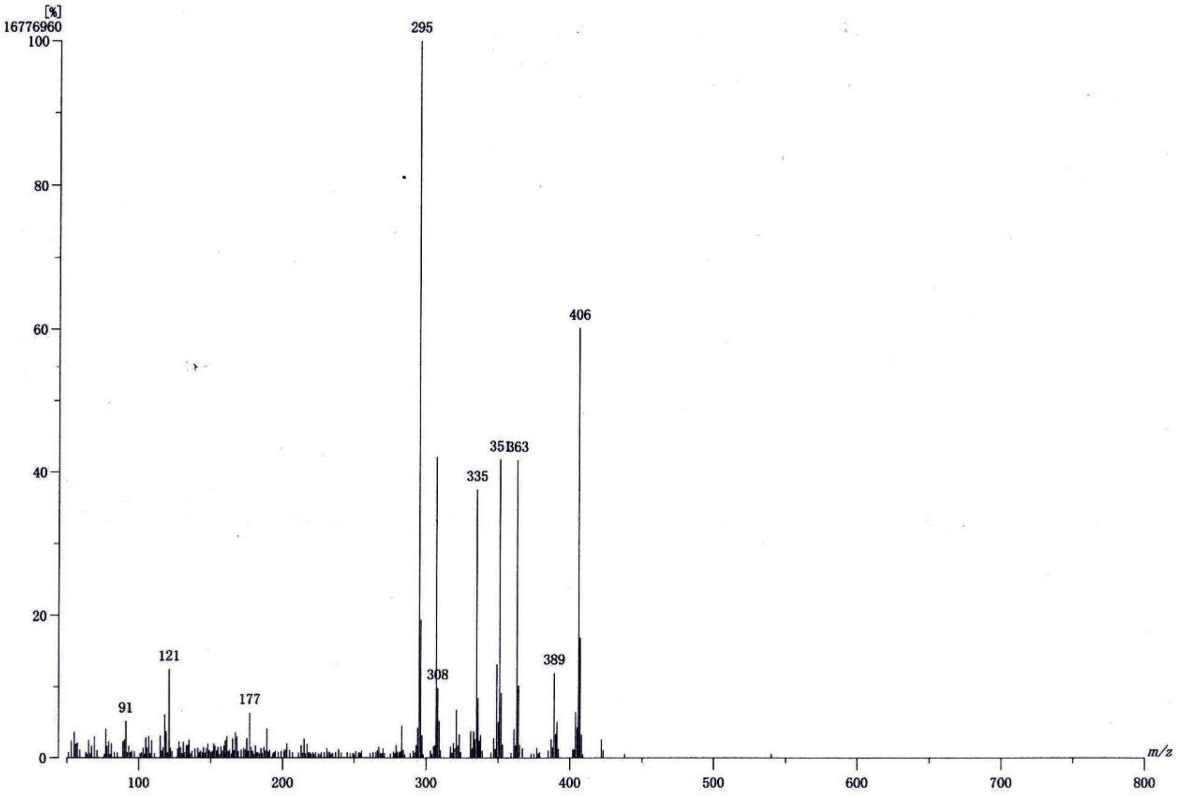

Instrument : MStation
Sample : –
Note : –
Inlet : Direct   Ion Mode : EI+
RT : 0.80 min   Scan# : 25
Elements : C 100/1, H 100/1, O 10/1
Mass Tolerance   : 1000ppm, 3mmu if m/z > 3
Unsaturation (U.S.) : –0.5 – 20.0

	Observed m/z	Int%	Err[ppm / mmu]	U.S.	Composition
1	406.1780	35.09	-0.1 / -0.0	13.0	C25 H26 O5

Figure 14. EIMS and HREIMS data of compound **7**

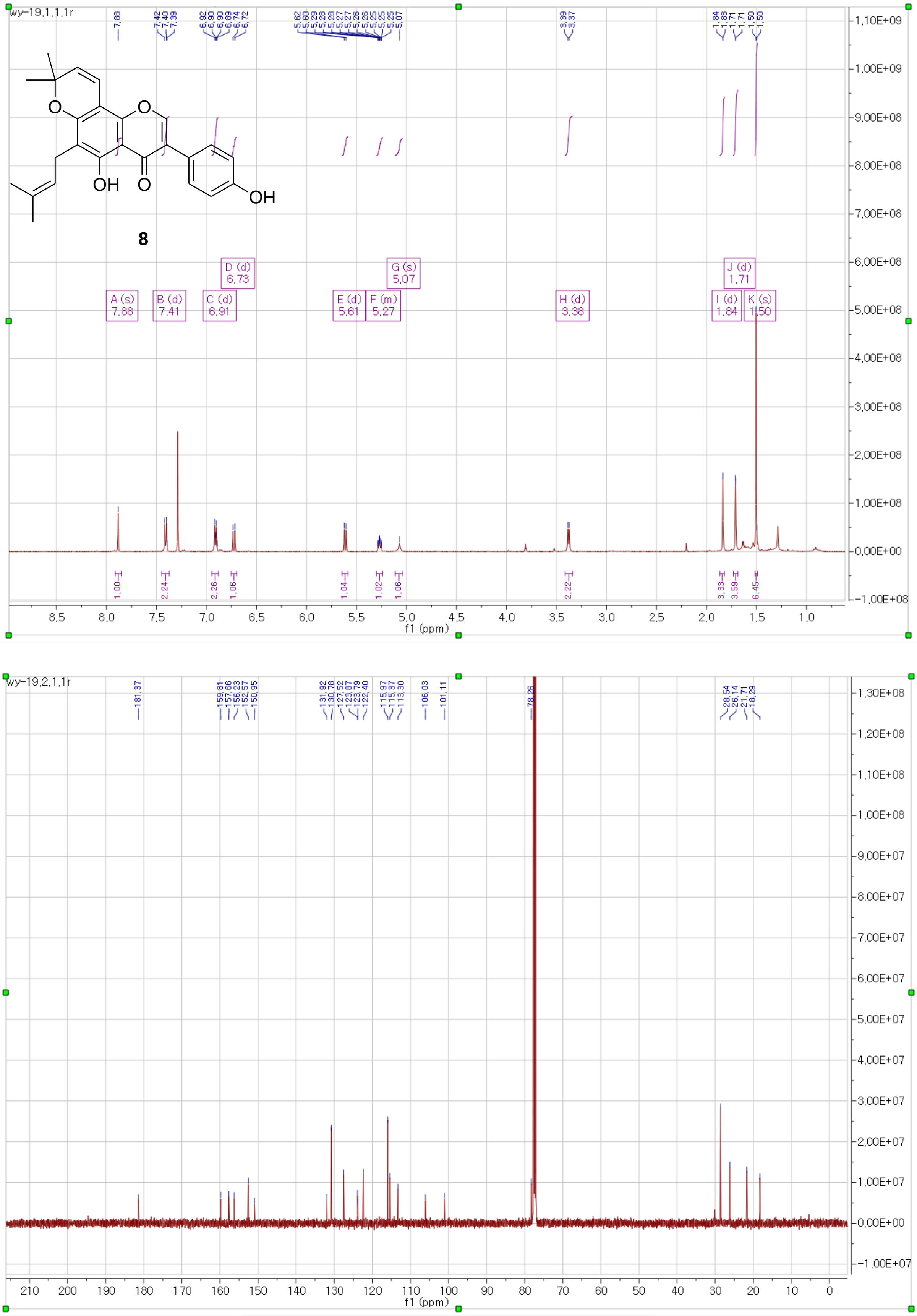

Figure 15. ^{1}H-NMR (500 MHz) and ^{13}C-NMR (125 MHz) spectrums of compound **8** ($CDCl_3$)

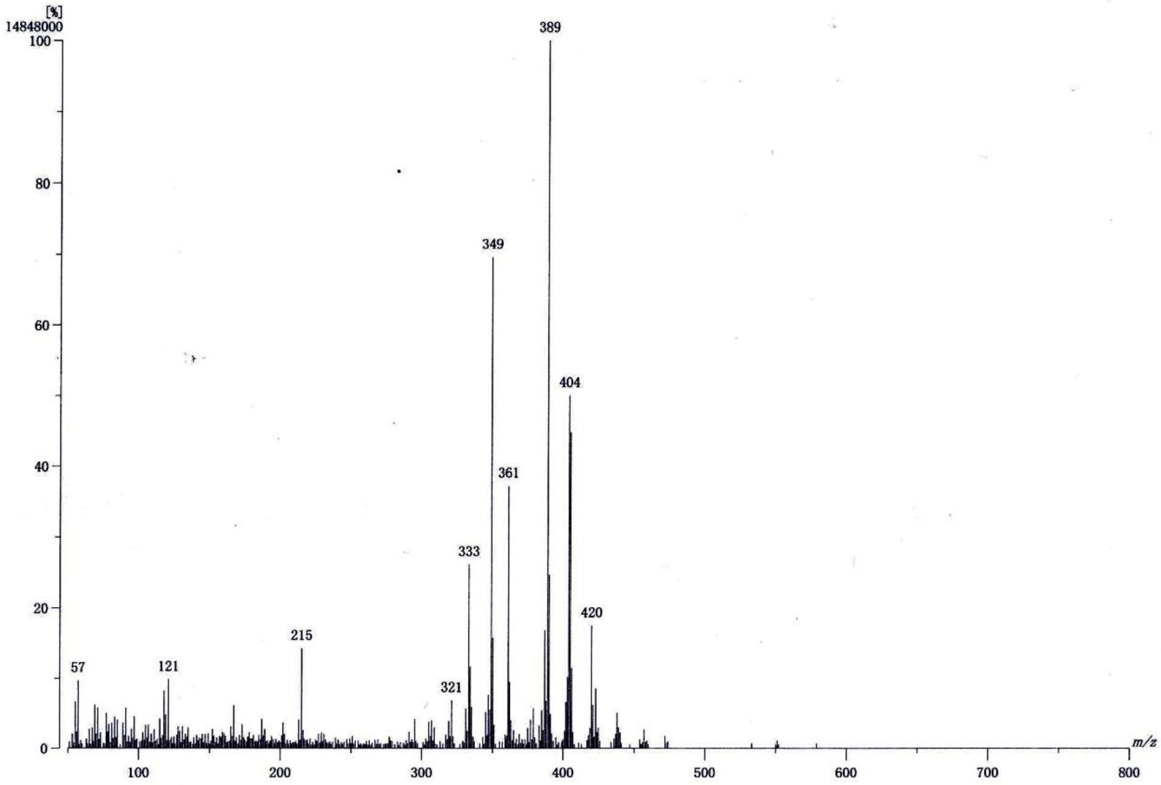

Instrument : MStation
Sample : –
Note : –
Inlet : Direct   Ion Mode : EI+
RT : 3.30 min   Scan# : 100
Elements : C 100/1, H 100/1, O 10/1
Mass Tolerance   : 1000ppm, 3mmu if m/z > 3
Unsaturation (U.S.) : –0.5 – 20.0

	Observed m/z	Int%	Err[ppm / mmu]	U.S.	Composition
1	404.1621	53.53	-0.7 / -0.3	14.0	C25 H24 O5

Figure 16. EIMS and HREIMS data of compound **8**

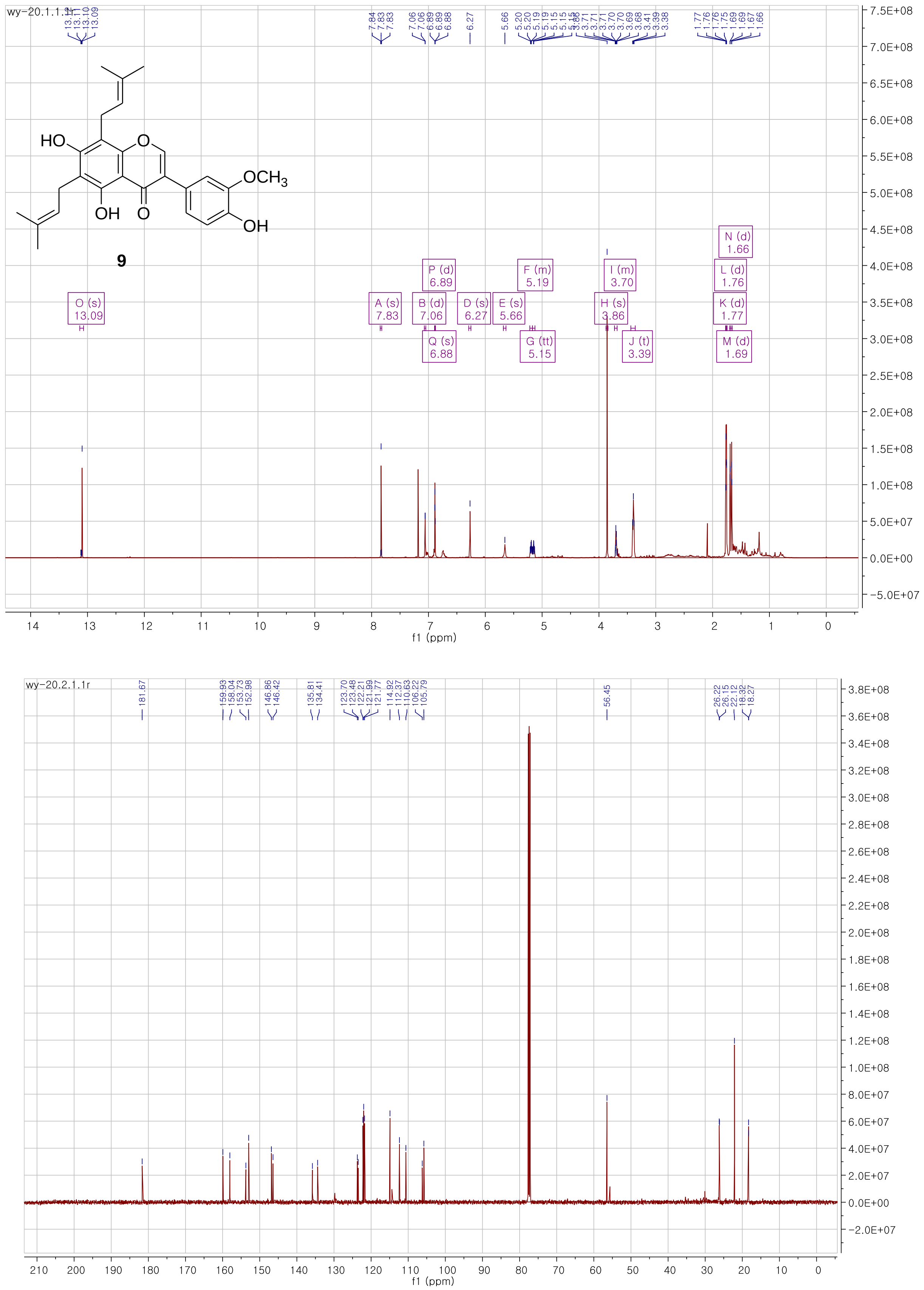

Figure 17. ^{1}H-NMR (500 MHz) and ^{13}C-NMR (125 MHz) spectrums of compound **9** ($CDCl_3$)

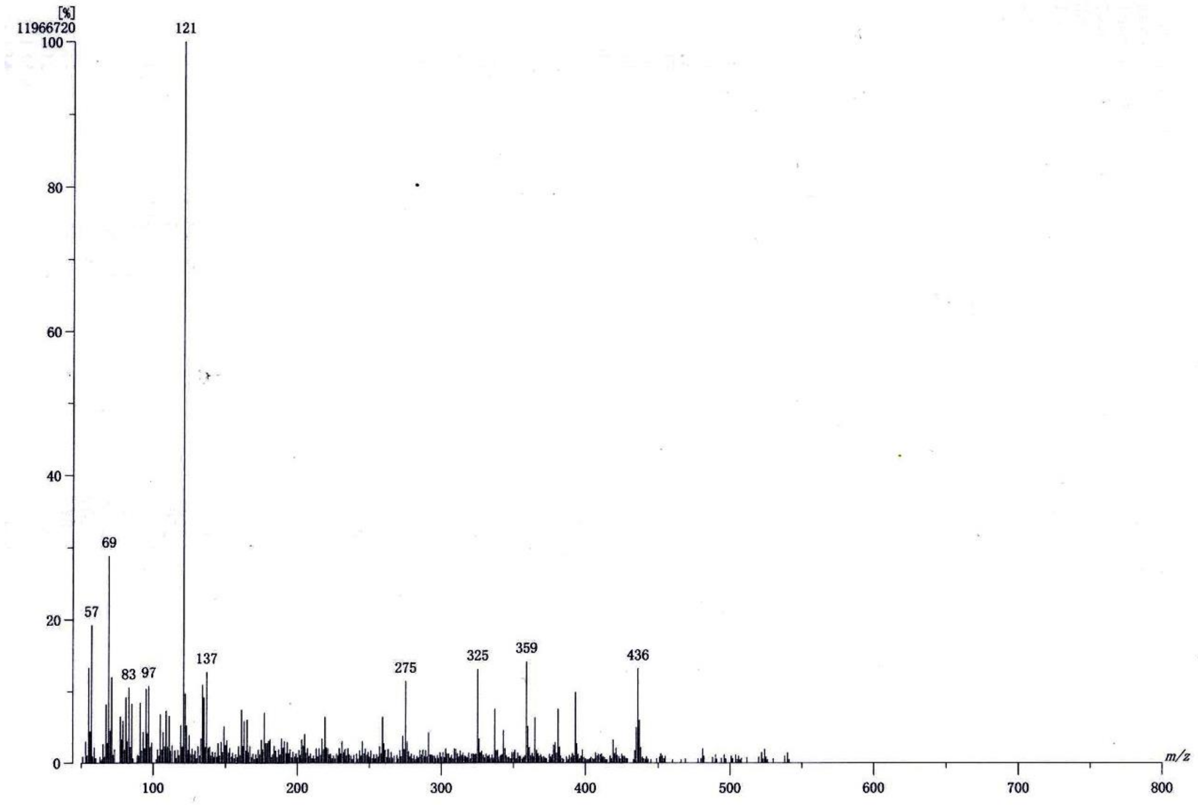

Instrument : MStation
Sample : –
Note : –
Inlet : Direct Ion Mode : EI+
RT : 0.64 min Scan# : 20
Elements : C 100/1, H 100/1, O 10/1
Mass Tolerance : 1000ppm, 3mmu if m/z > 3
Unsaturation (U.S.) : –0.5 – 20.0

	Observed m/z	Int%	Err[ppm / mmu]	U.S.	Composition
1	436.1888	54.16	+0.5 / +0.2	13.0	C26 H28 O6

Figure 18. EIMS and HREIMS data of compound **9**

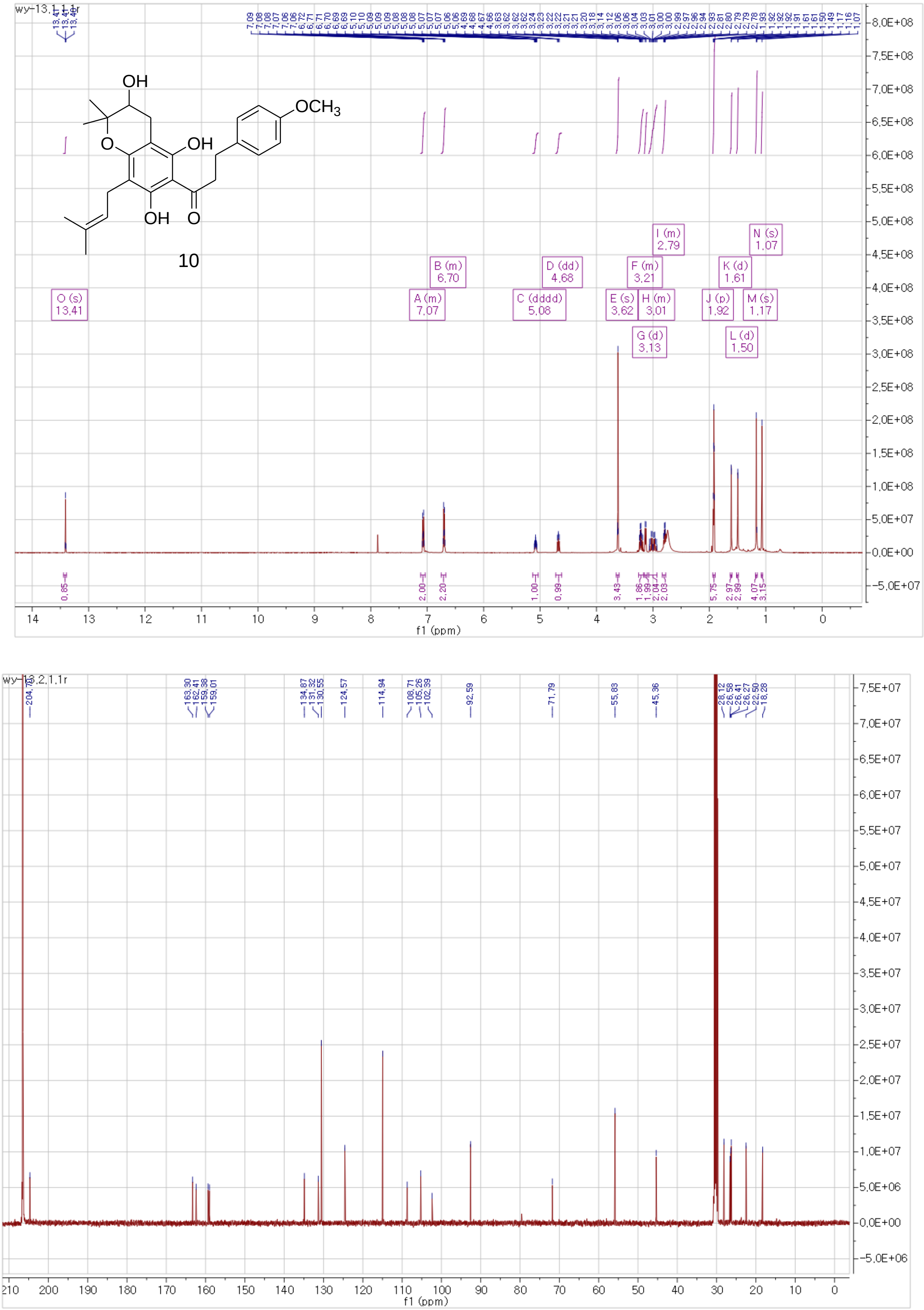

Figure 19. ^{1}H-NMR (500 MHz) and ^{13}C-NMR (125 MHz) spectrums of compound **10** (Acetone-d_6)

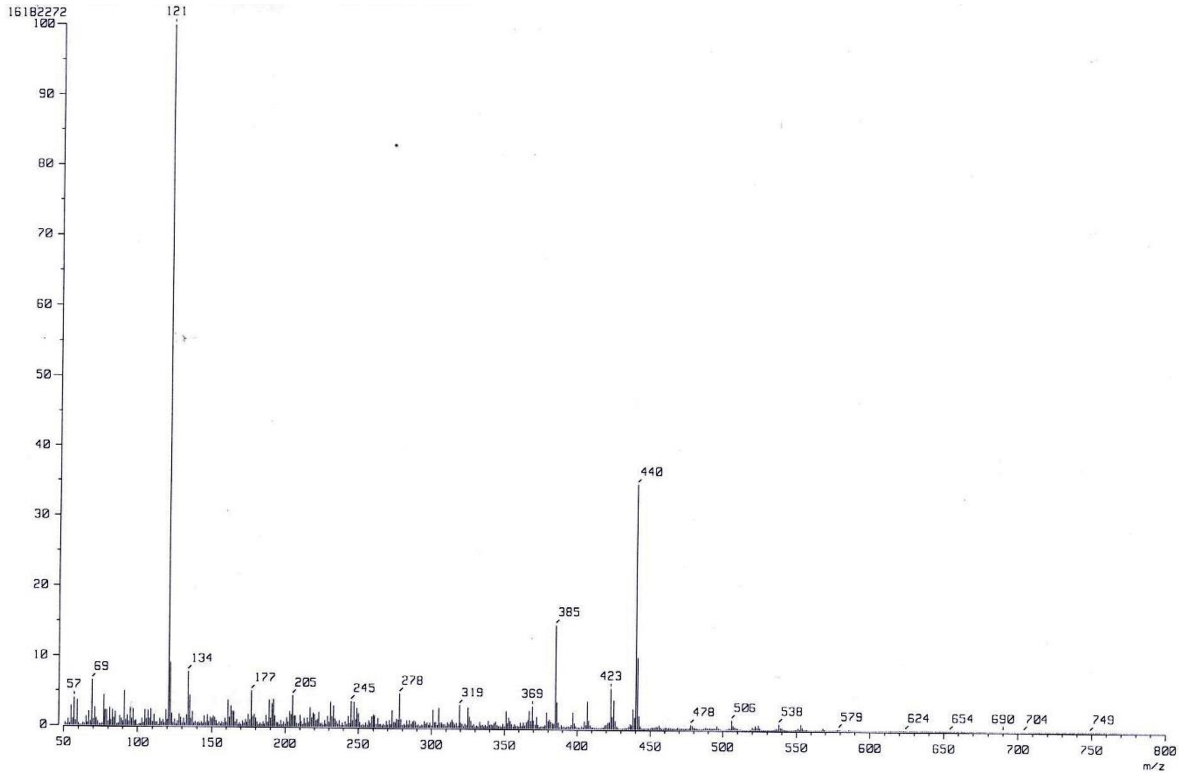

Instrument : MStation
Sample : –
Note : –
Inlet : Direct Ion Mode : EI+
RT : 1.47 min Scan# : 45
Elements : C 100/1, H 100/1, O 10/1
Mass Tolerance : 1000ppm, 3mmu if m/z > 3
Unsaturation (U.S.) : –0.5 – 20.0

	Observed m/z	Int%	Err[ppm / mmu]	U.S.	Composition
1	440.2199	53.13	+0.0 / +0.0	11.0	C26 H32 O6

Figure 20. EIMS and HREIMS data of compound **10**

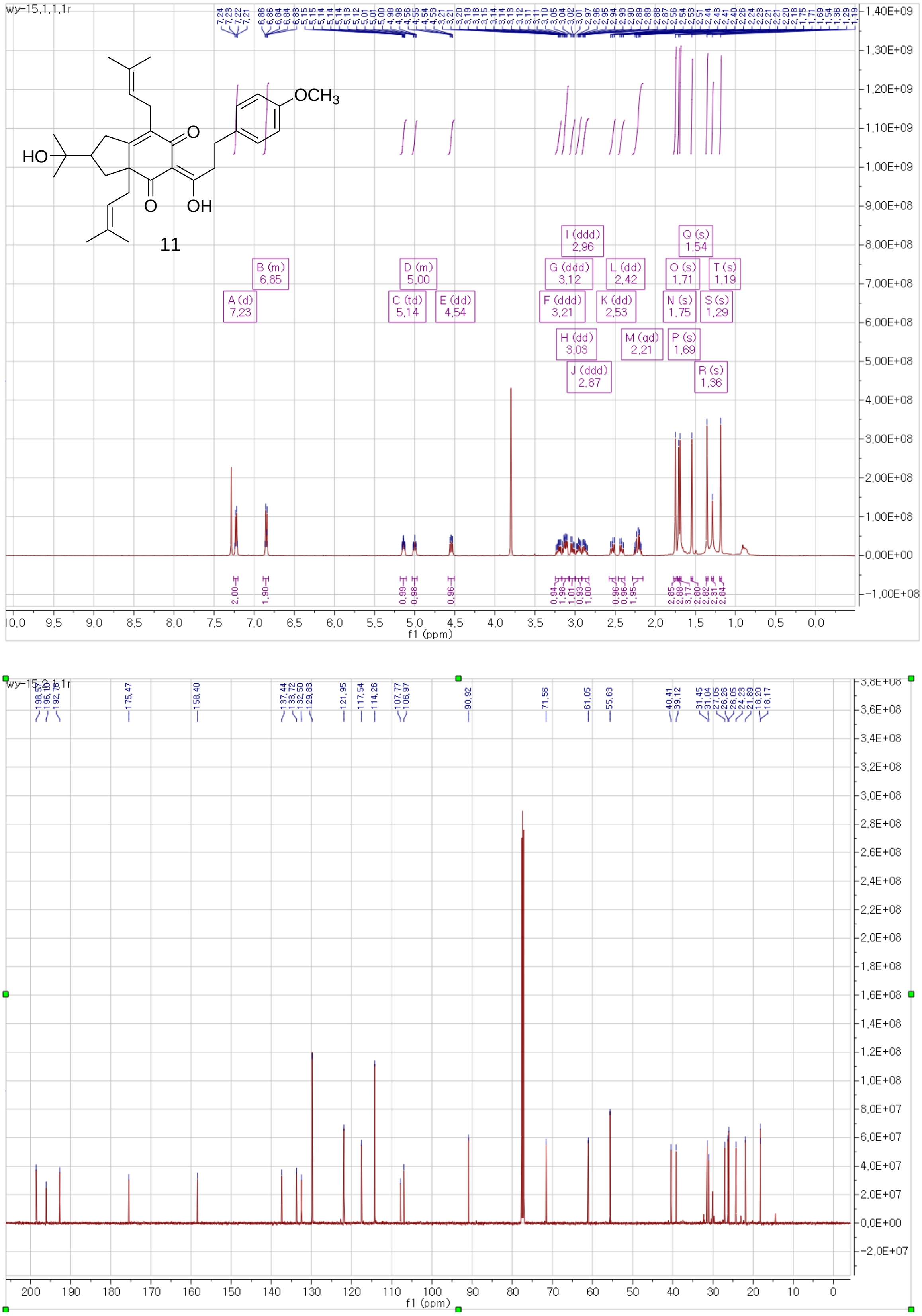

Figure 21. ^{1}H-NMR (500 MHz) and ^{13}C-NMR (125 MHz) spectrums

of compound **11** ($CDCl_3$)

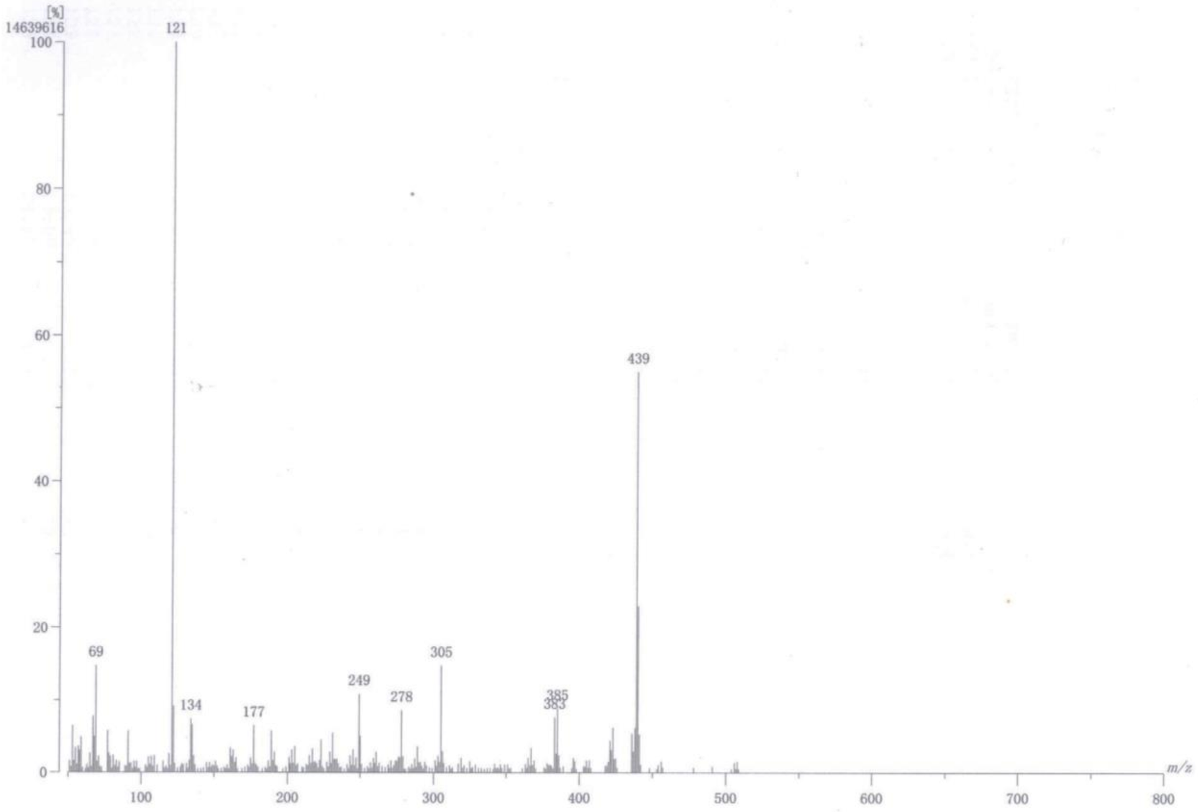

Instrument : MStation
Sample : –
Note : –
Inlet : Direct Ion Mode : EI+
RT : 0.80 min Scan# : 25
Elements : C 100/1, H 100/1, O 10/1
Mass Tolerance : 1000ppm, 3mmu if m/z > 3
Unsaturation (U.S.) : –0.5 – 20.0

	Observed m/z	Int%	Err[ppm / mmu]	U.S.	Composition
1	508.2820	100.00	-1.0 / -0.5	12.0	C31 H40 O6

Figure 22. ^{1}H-NMR (500 MHz) and ^{13}C-NMR (125 MHz) spectrums of compound **11** ($CDCl_3$)

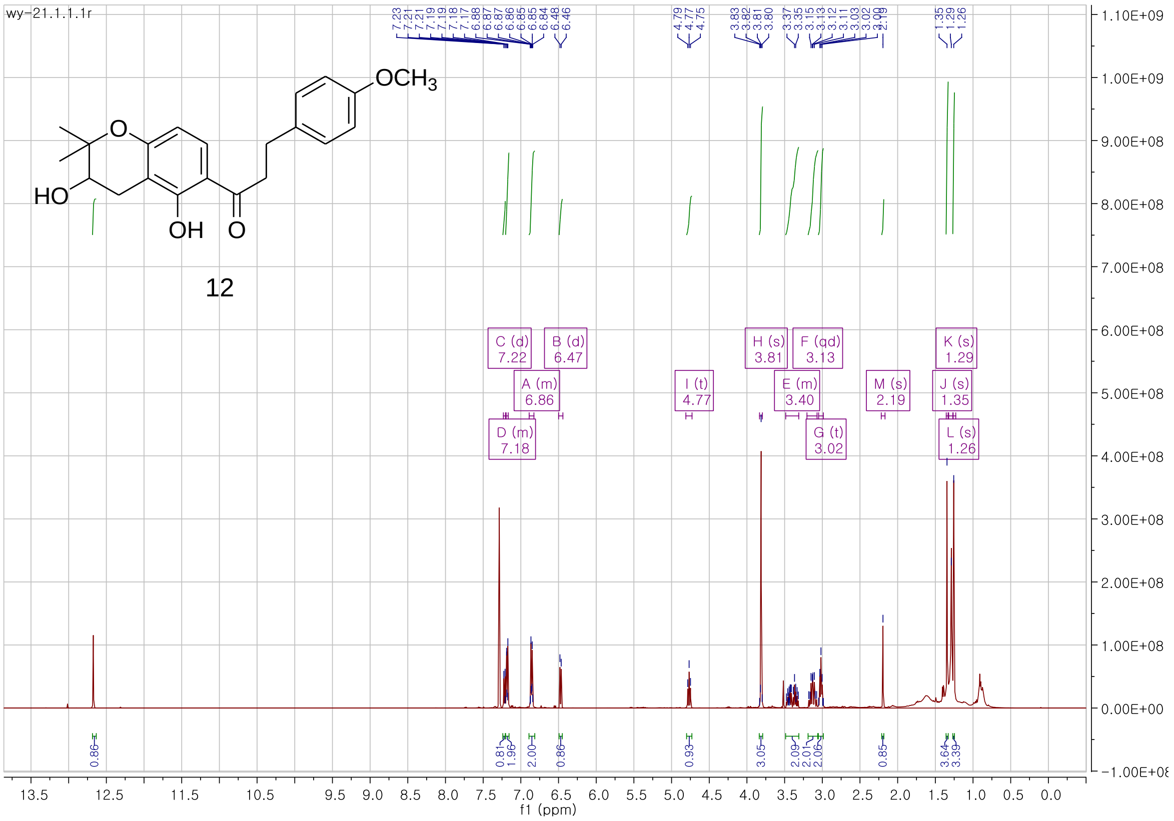

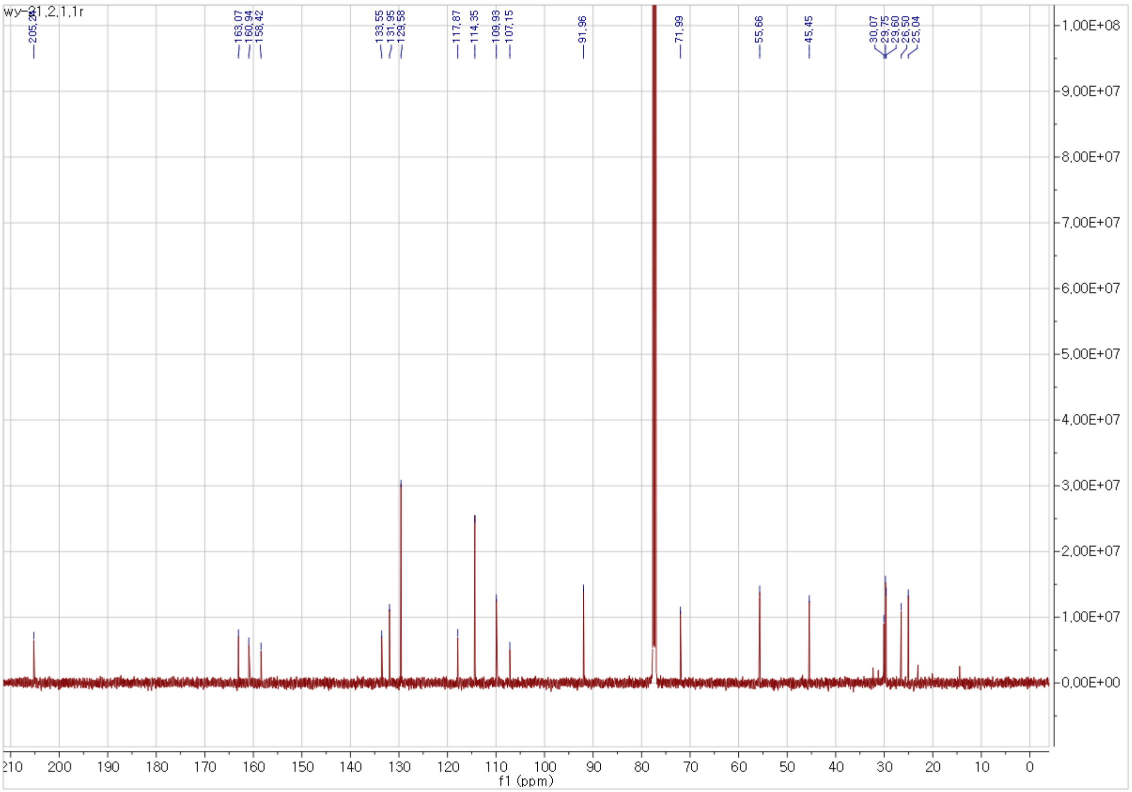

Figure 23. ^{1}H-NMR (500 MHz) and ^{13}C-NMR (125 MHz) spectrums of compound **12** ($CDCl_3$)

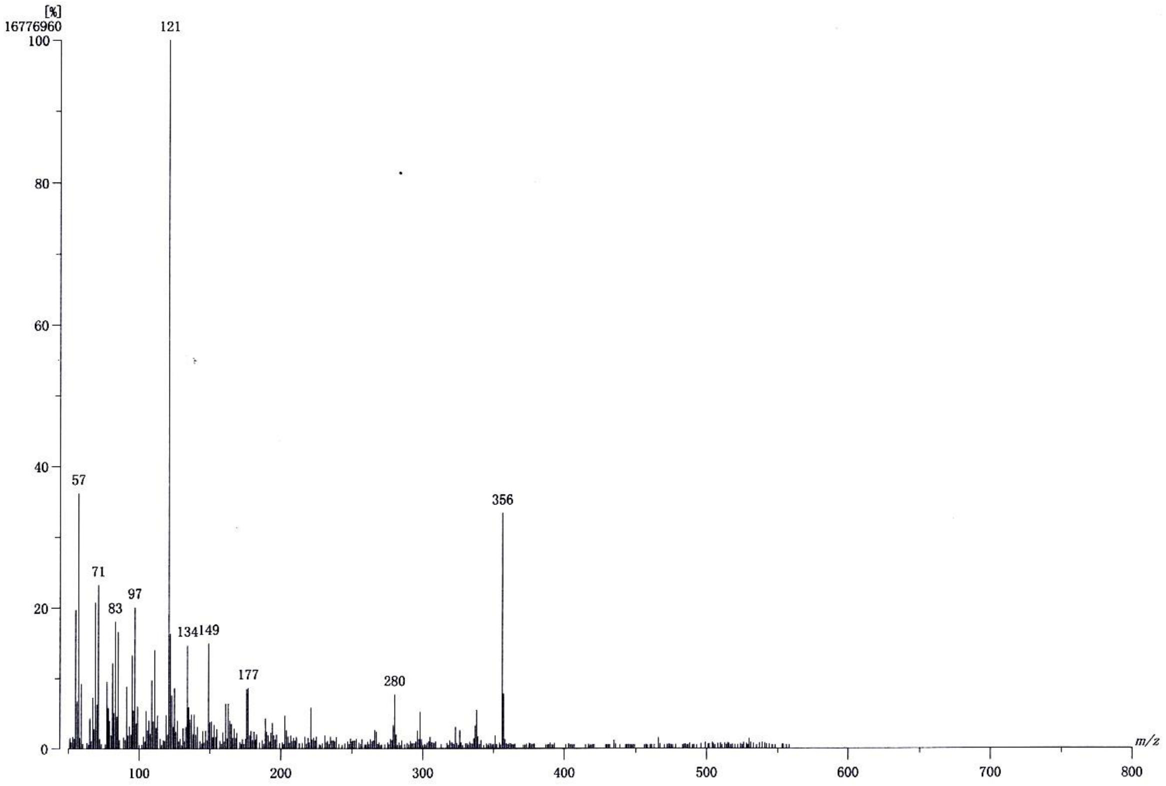

Instrument : MStation
Sample : -
Note : -
Inlet : Direct Ion Mode : EI+
RT : 1.00 min Scan# : 31
Elements : C 100/1, H 100/1, O 10/1
Mass Tolerance : 1000ppm, 3mmu if m/z > 3
Unsaturation (U.S.) : -0.5 - 20.0

	Observed m/z	Int%	Err[ppm / mmu]	U.S.	Composition
1	356.1625	61.52	+0.4 / +0.1	10.0	C21 H24 O5

Figure 24. EIMS and HREIMS data of compound **12**

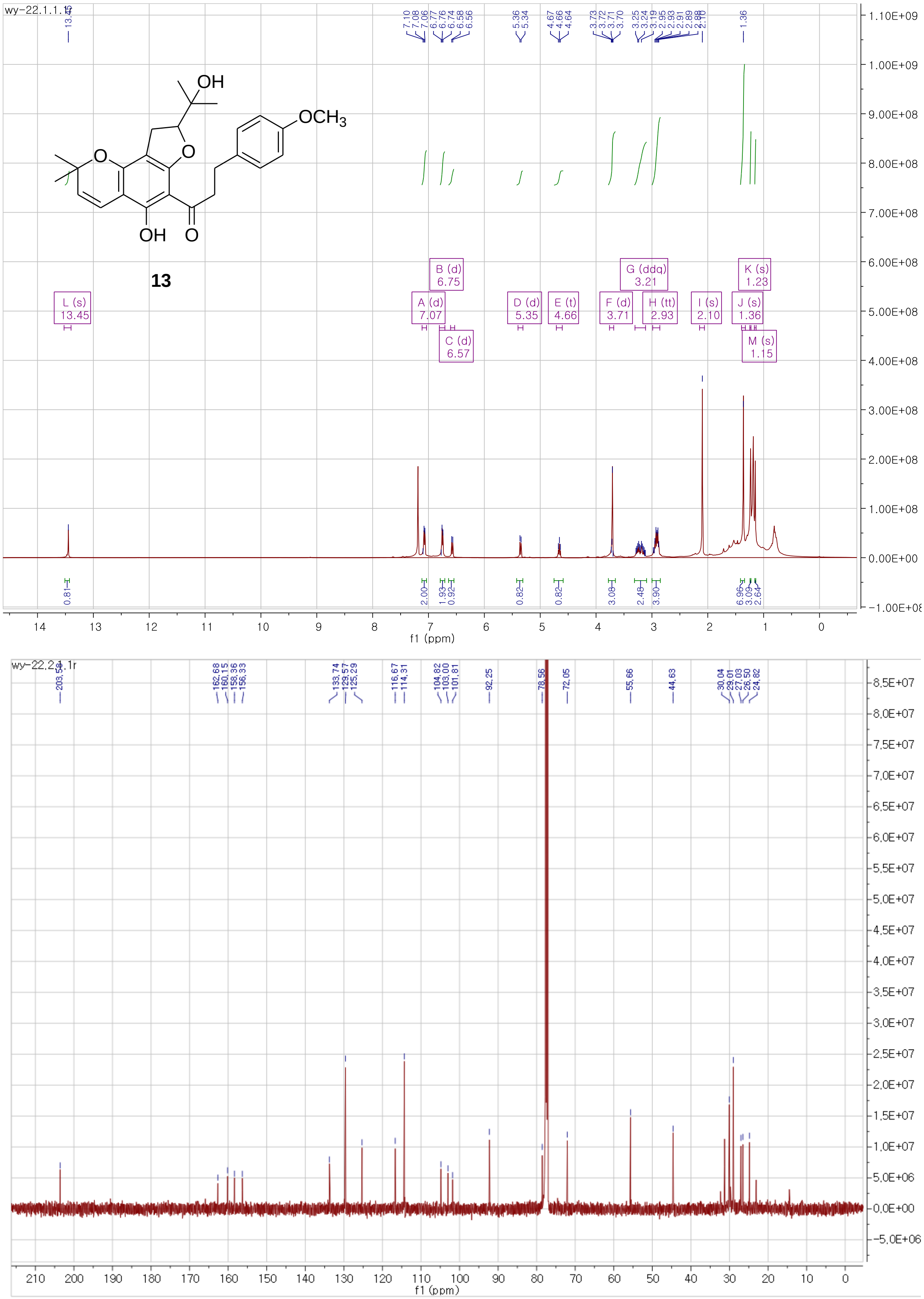

Figure 25. ^{1}H-NMR (500 MHz) and ^{13}C-NMR (125 MHz) spectrums of compound **13** ($CDCl_3$)

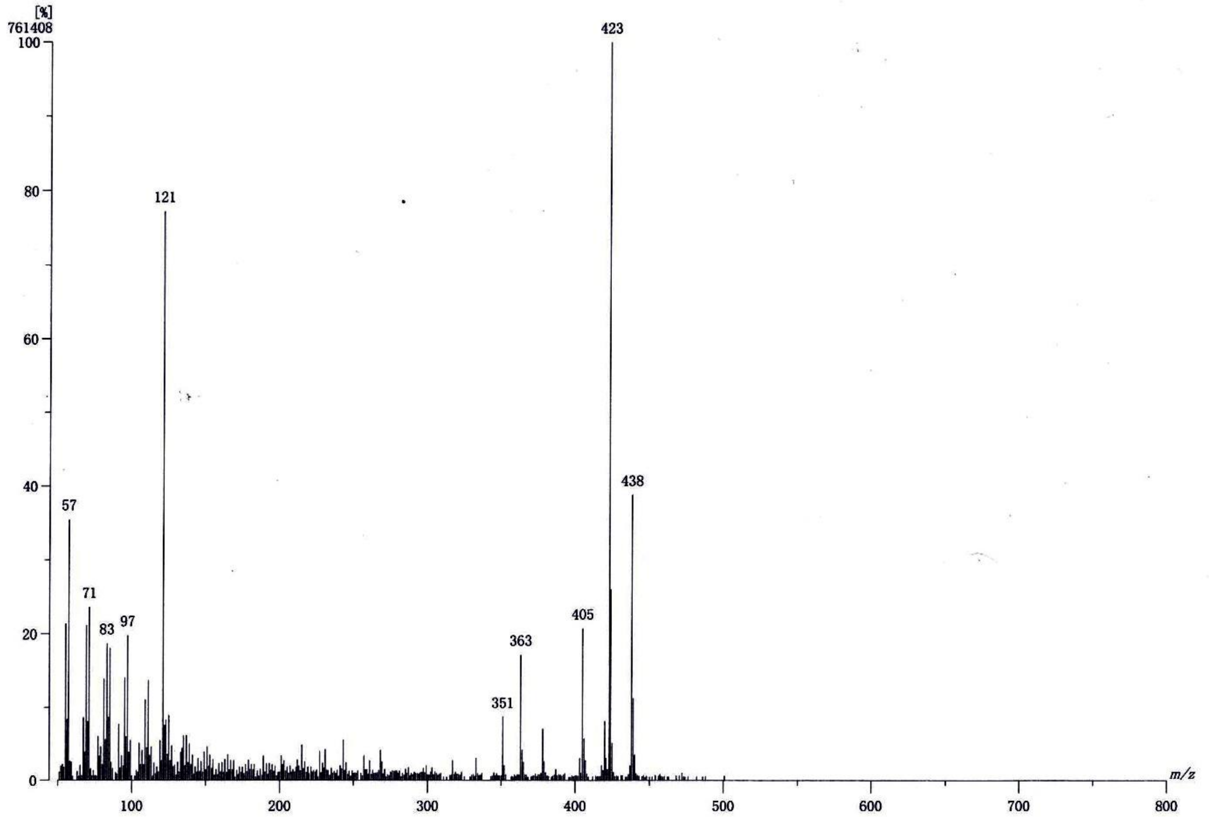

Instrument : MStation
Sample : -
Note : -
Inlet : Direct   Ion Mode : EI+
RT : 2.64 min   Scan# : 80
Elements : C 100/1, H 100/1, O 10/1
Mass Tolerance   : 1000ppm, 3mmu if m/z > 3
Unsaturation (U.S.) : -0.5 - 20.0

	Observed m/z	Int%	Err[ppm / mmu]	U.S.	Composition
1	438.2039	40.22	-0.8 / -0.3	12.0	C26 H30 O6

Figure 26. EIMS and HREIMS data of compound **13**

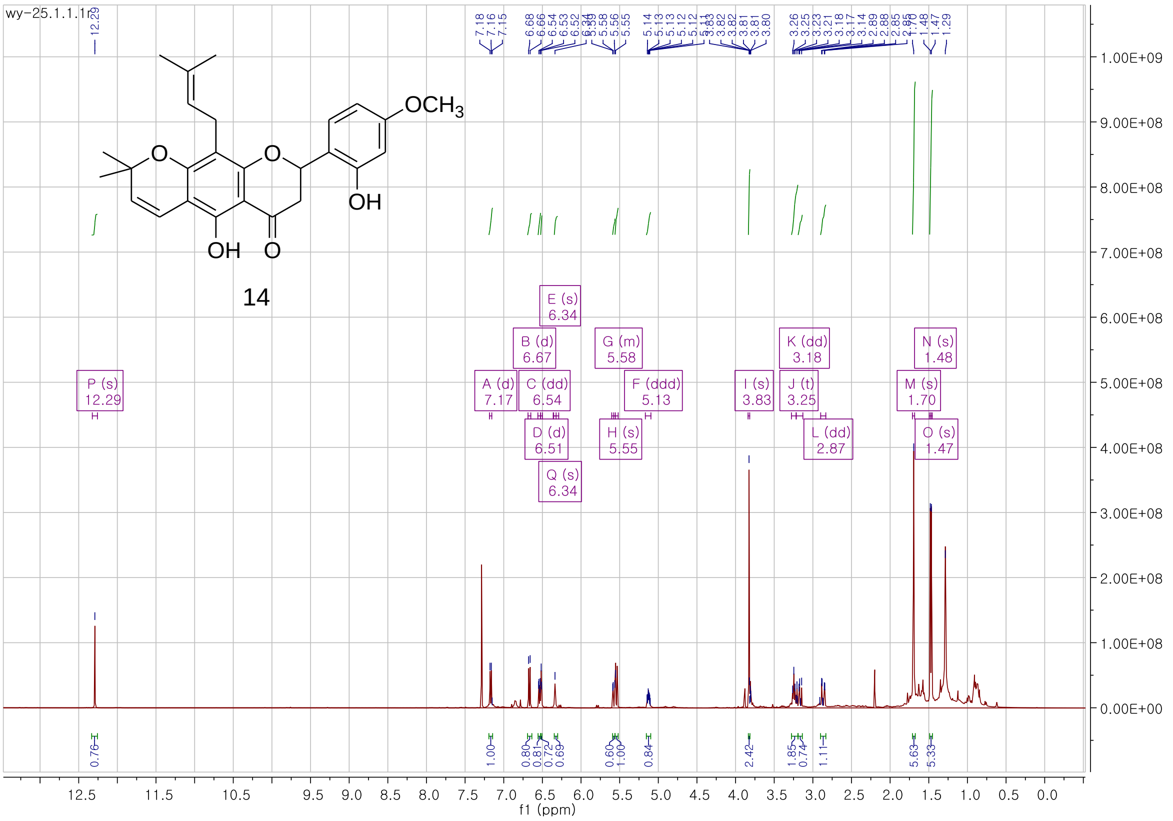

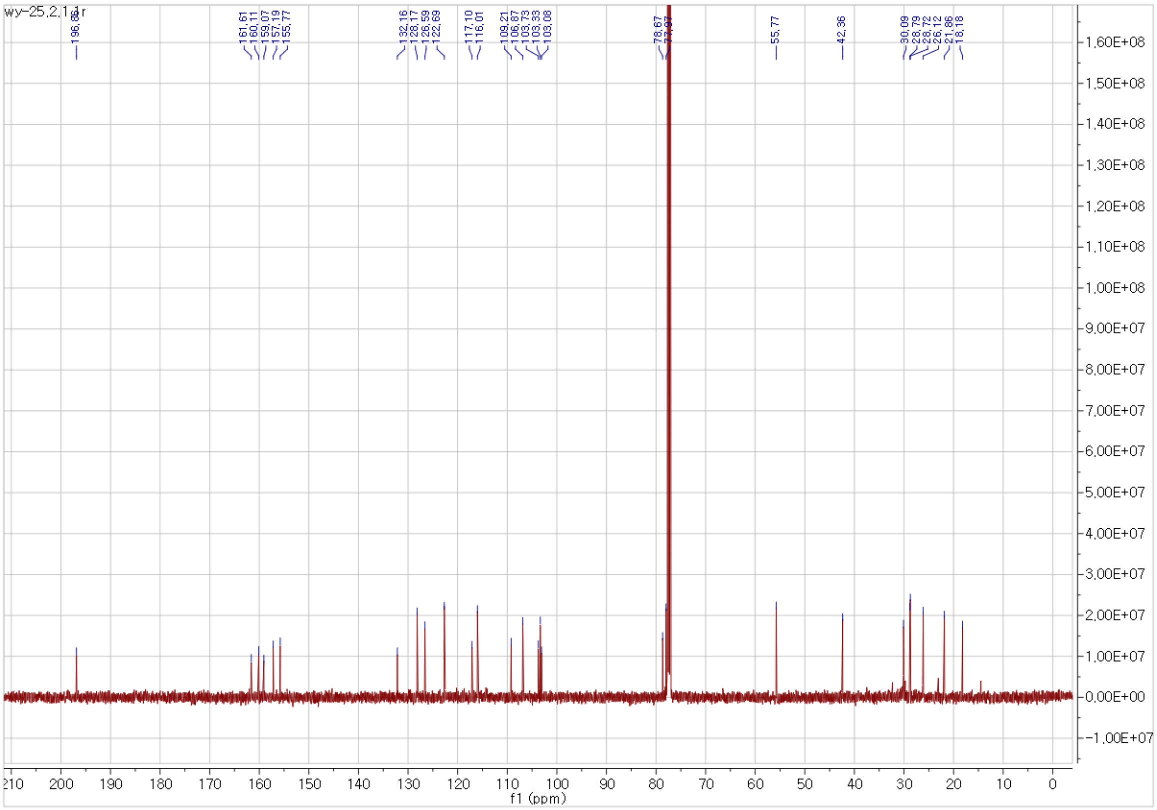

Figure 27. ^{1}H-NMR (500 MHz) and ^{13}C-NMR (125 MHz) spectrums

of compound **14** ($CDCl_3$)

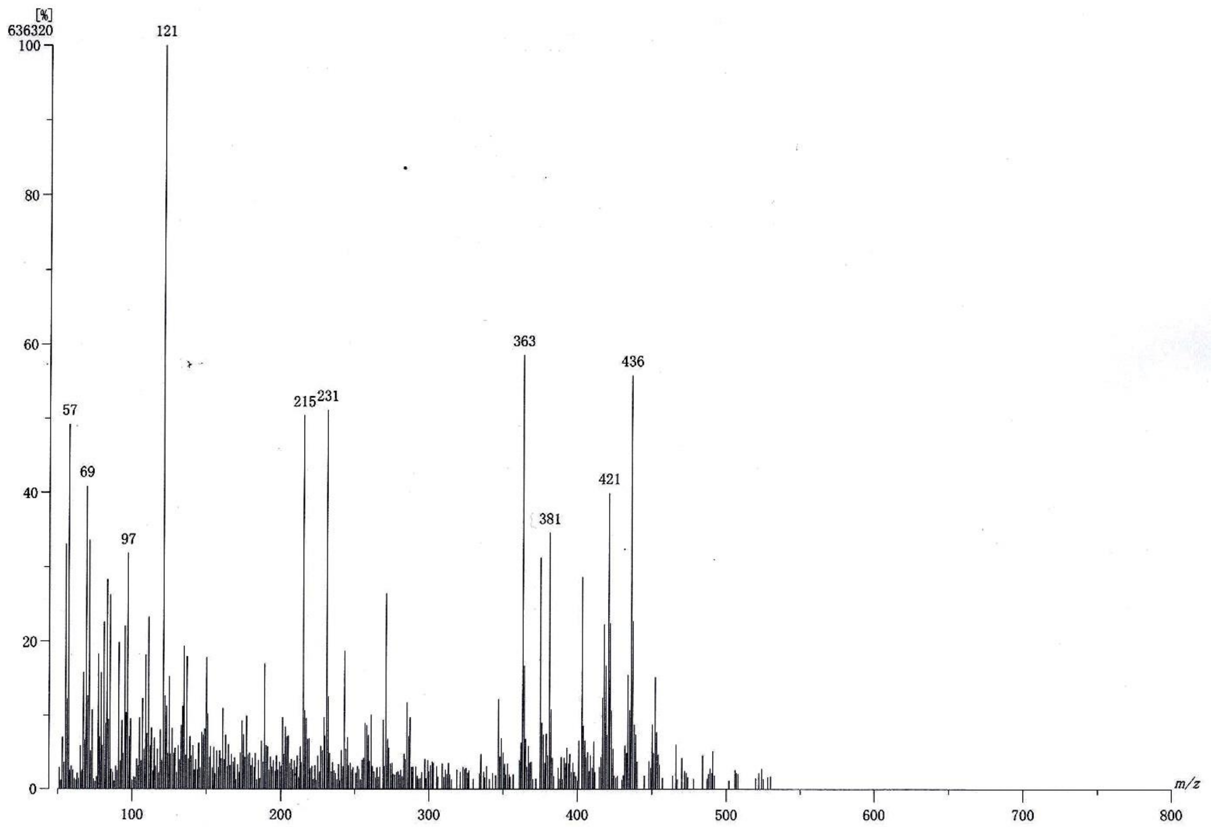

Instrument : MStation
Sample : −
Note : −
Inlet : Direct Ion Mode : EI+
RT : 1.14 min Scan# : 35
Elements : C 100/1, H 100/1, O 10/1
Mass Tolerance : 1000ppm, 3mmu if m/z > 3
Unsaturation (U.S.) : −0.5 − 20.0

	Observed m/z	Int%	Err[ppm / mmu]	U.S.	Composition
1	436.1882	77.13	−0.9 / −0.4	13.0	C26 H28 O6

Figure 28. EIMS and HREIMS data of compound **14**

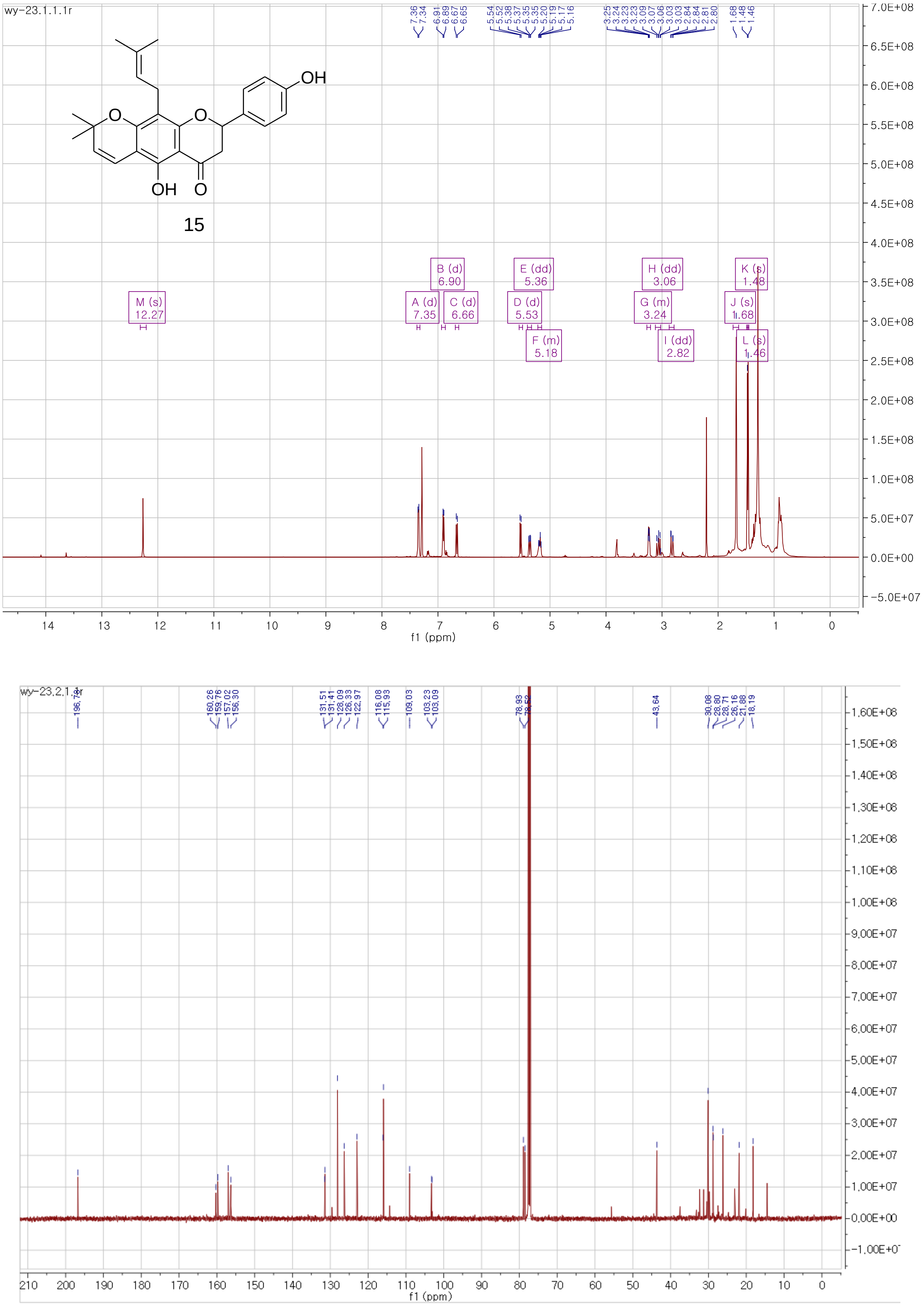

Figure 29. ^{1}H-NMR (500 MHz) and ^{13}C-NMR (125 MHz) spectrums of compound **15** ($CDCl_3$)

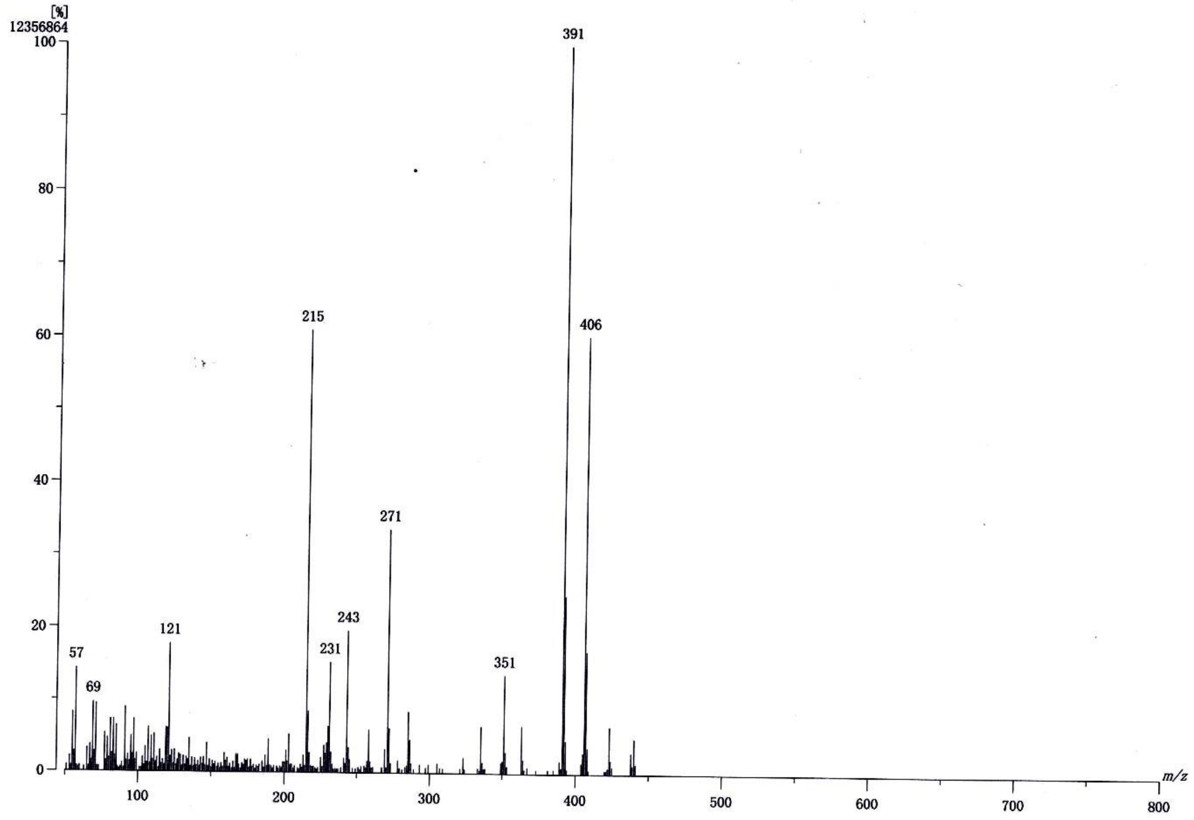

Instrument : MStation
Sample : –
Note : –
Inlet : Direct Ion Mode : EI+
RT : 1.30 min Scan# : 40
Elements : C 100/1, H 100/1, O 10/1
Mass Tolerance : 1000ppm, 3mmu if m/z > 3
Unsaturation (U.S.) : –0.5 – 20.0

	Observed m/z	Int%	Err[ppm / mmu]	U.S.	Composition
1	406.1778	41.79	–0.6 / –0.2	13.0	C25 H26 O5

Figure 30. EIMS and HREIMS data of compound **15**

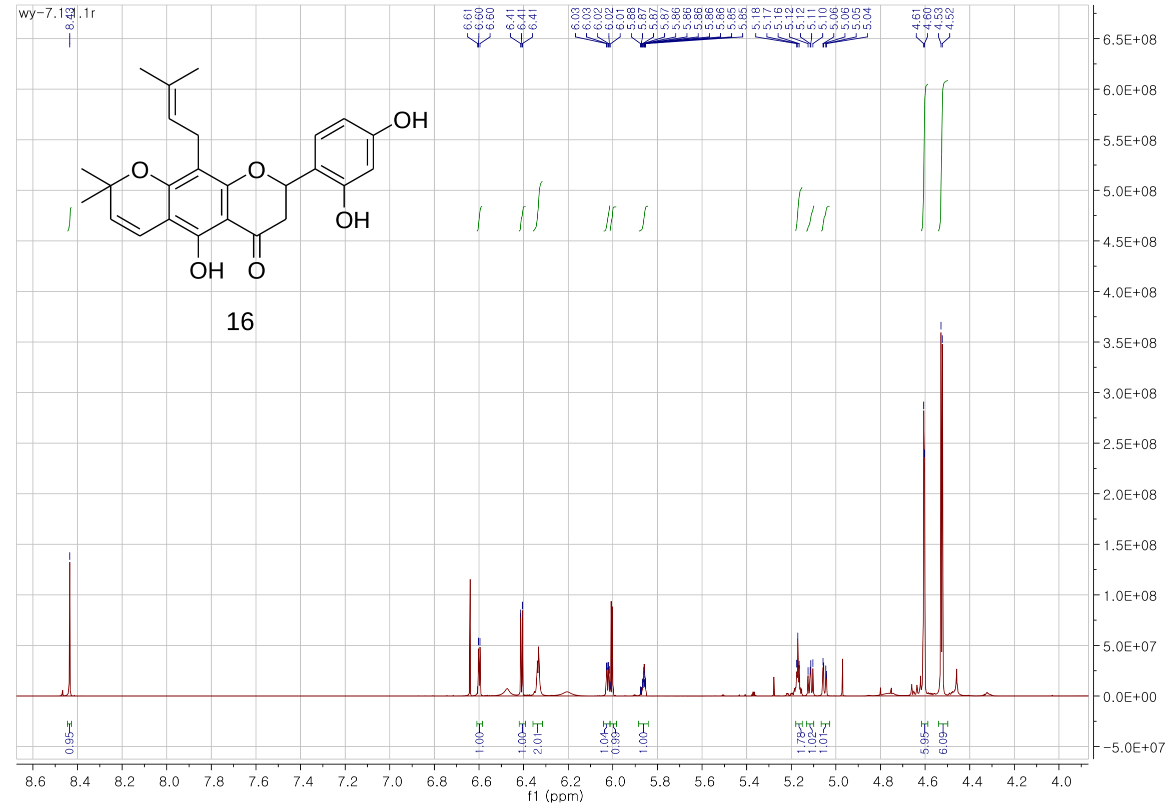

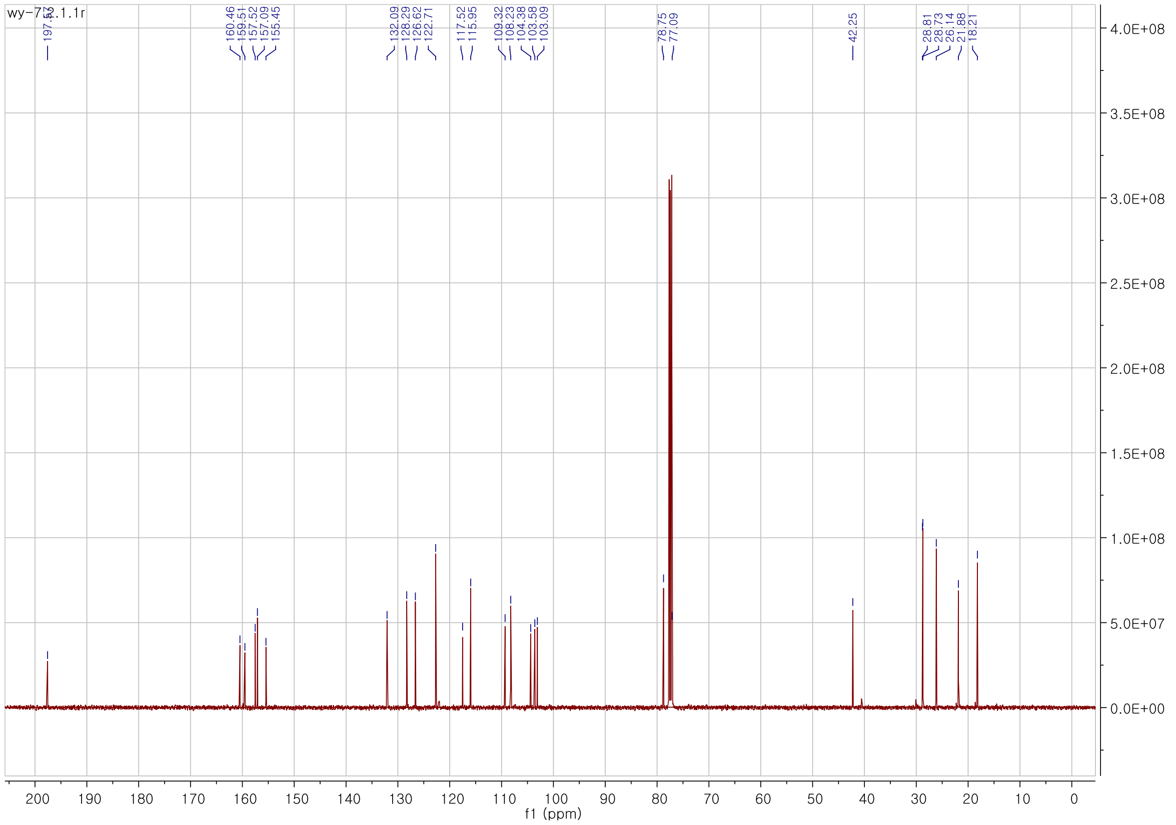

Figure 31. ¹H-NMR (500 MHz) and ¹³C-NMR (125 MHz) spectrums

of compound **16** ($CDCl_3$)

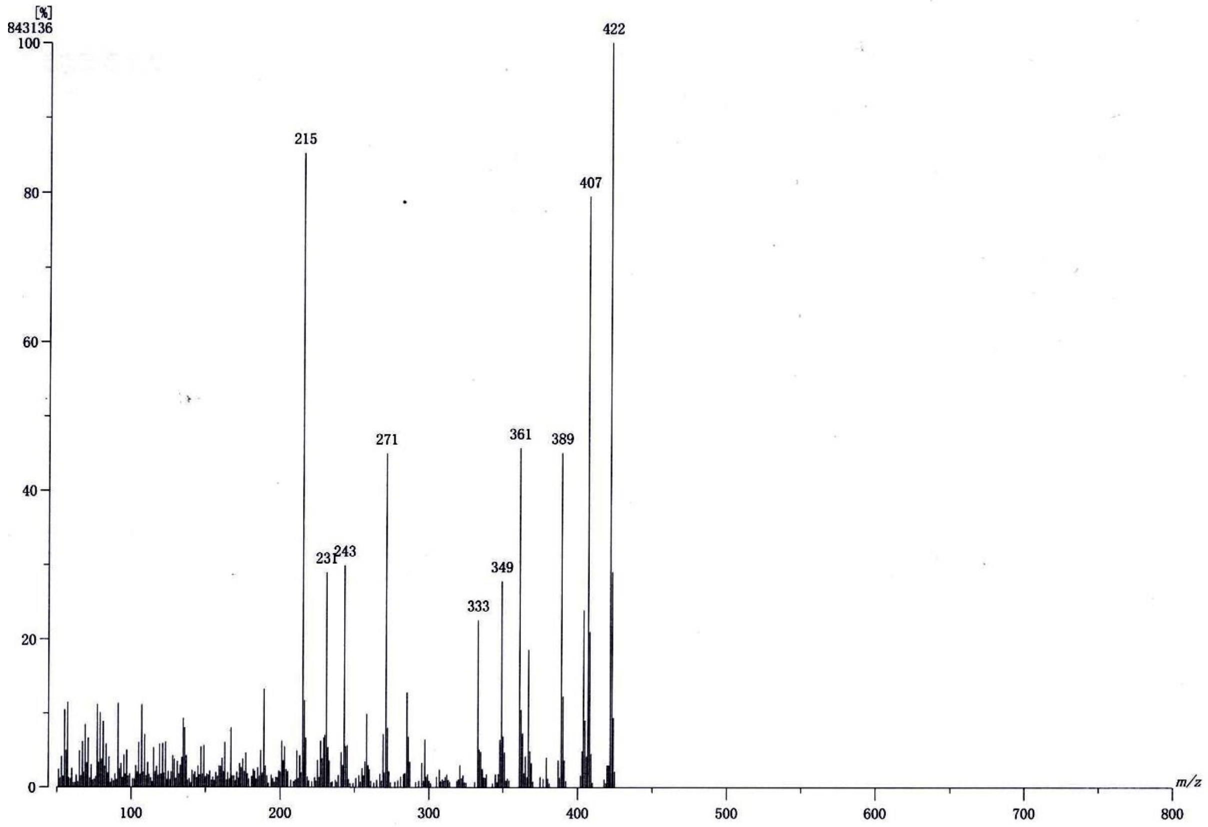

Instrument : MStation
Sample : -
Note : -
Inlet : Direct Ion Mode : EI+
RT : 1.67 min Scan# : 51
Elements : C 100/1, H 100/1, O 10/1
Mass Tolerance : 1000ppm, 3mmu if m/z > 3
Unsaturation (U.S.) : -0.5 - 20.0

	Observed m/z	Int%	Err[ppm / mmu]	U.S.	Composition
1	422.1727	100.00	-0.6 / -0.2	13.0	C25 H26 O6

Figure 32. EIMS and HREIMS data of compound **16**

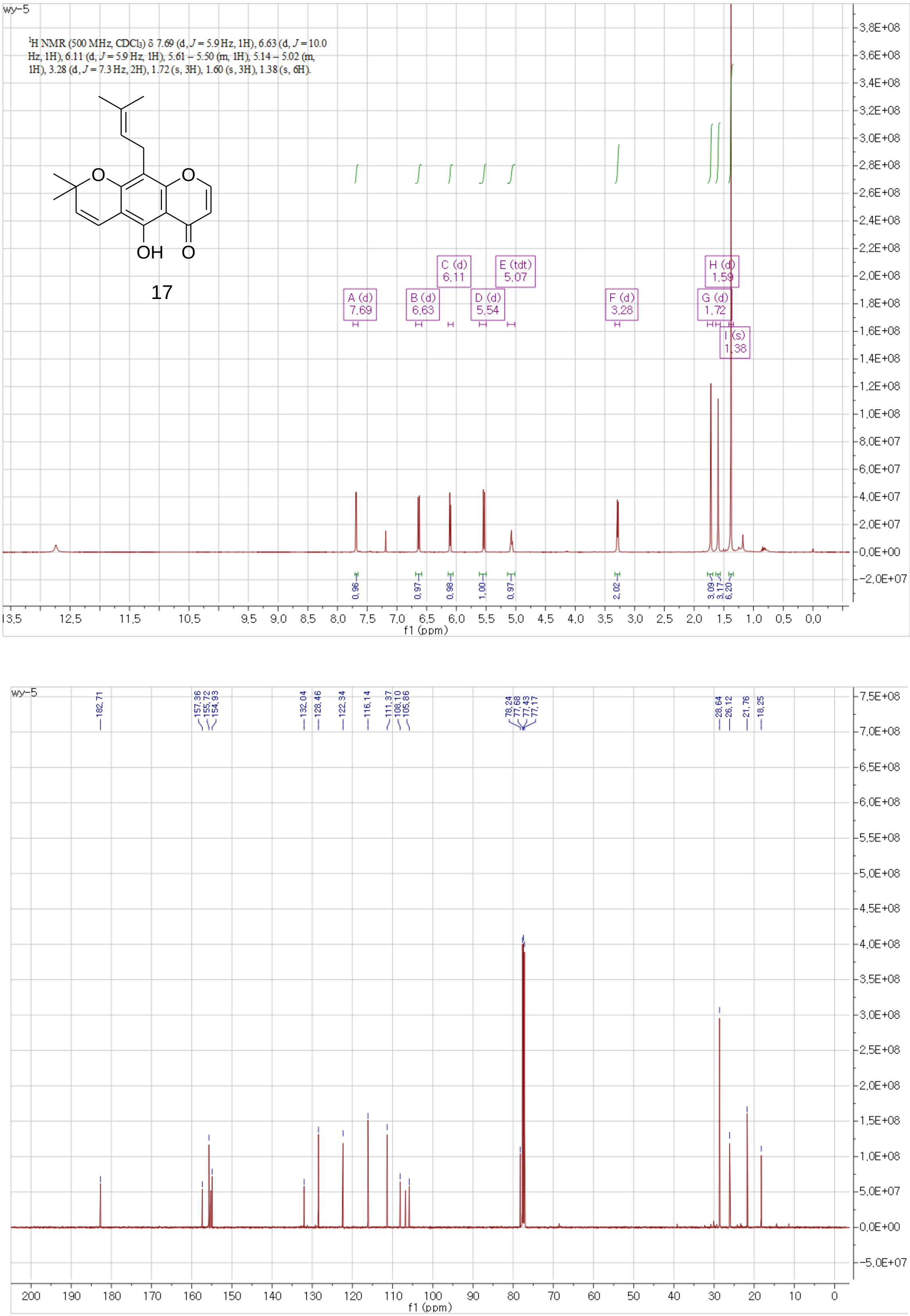

Figure 33. ^{1}H-NMR (500 MHz) and ^{13}C-NMR (125 MHz) spectrums of compound **17** ($CDCl_3$)

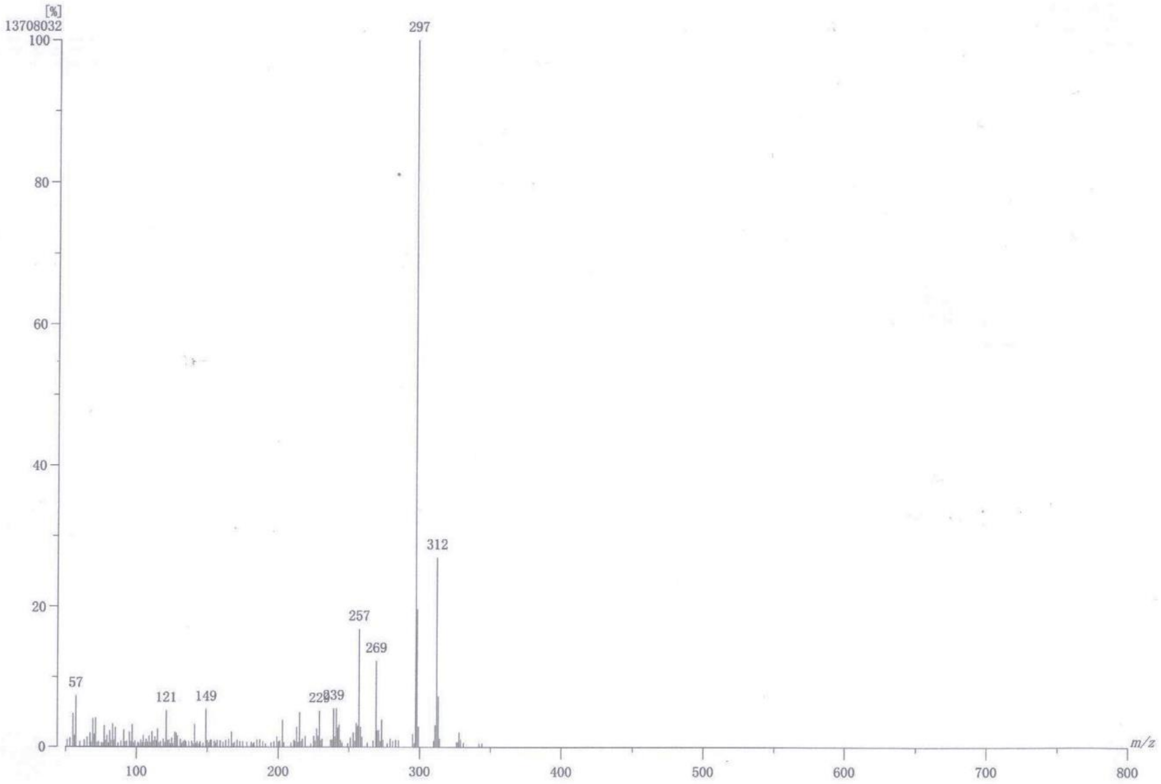

Instrument : MStation
Sample : –
Note : –
Inlet : Direct   Ion Mode : EI+
RT : 0.27 min   Scan# : 9
Elements : C 100/1, H 100/1, O 10/1
Mass Tolerance   : 1000ppm, 3mmu if m/z > 3
Unsaturation (U.S.) : –0.5 – 20.0

	Observed m/z	Int%	Err[ppm / mmu]	U.S.	Composition
1	312.1361	100.00	–0.2 / –0.1	10.0	C19 H20 O4

Figure 34. EIMS and HREIMS data of compound **17**

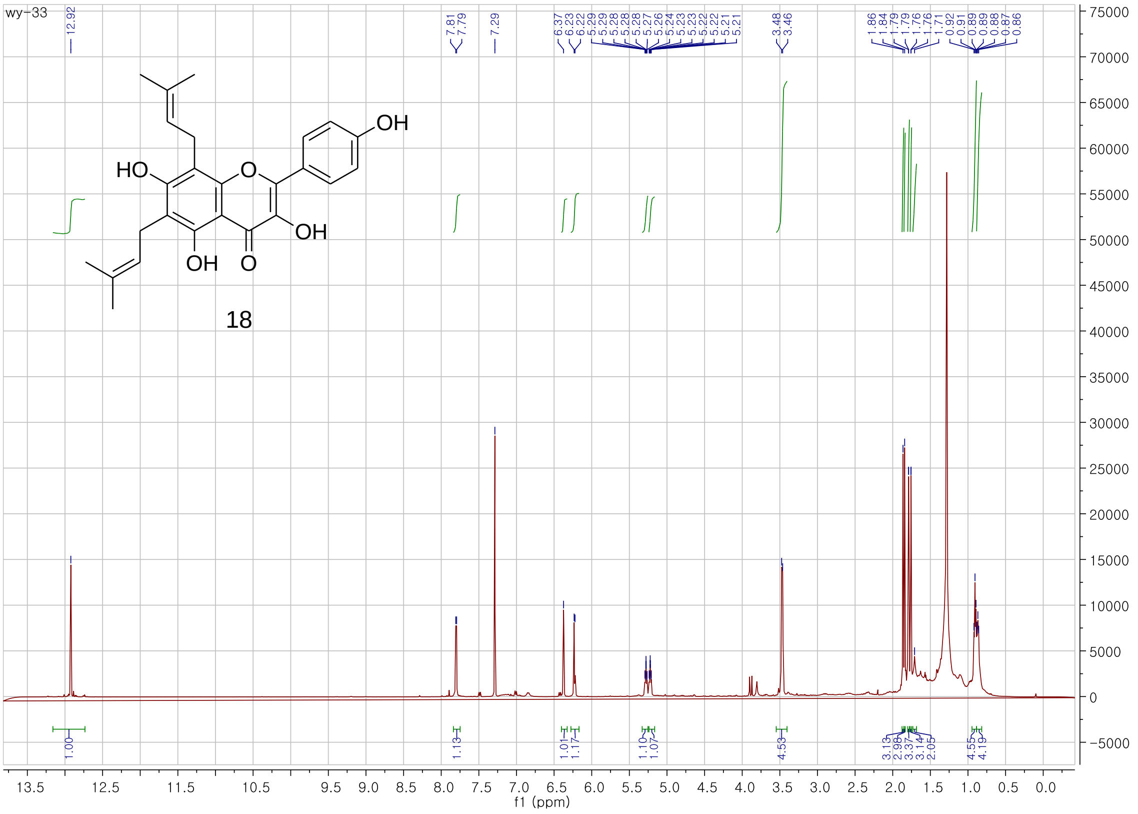

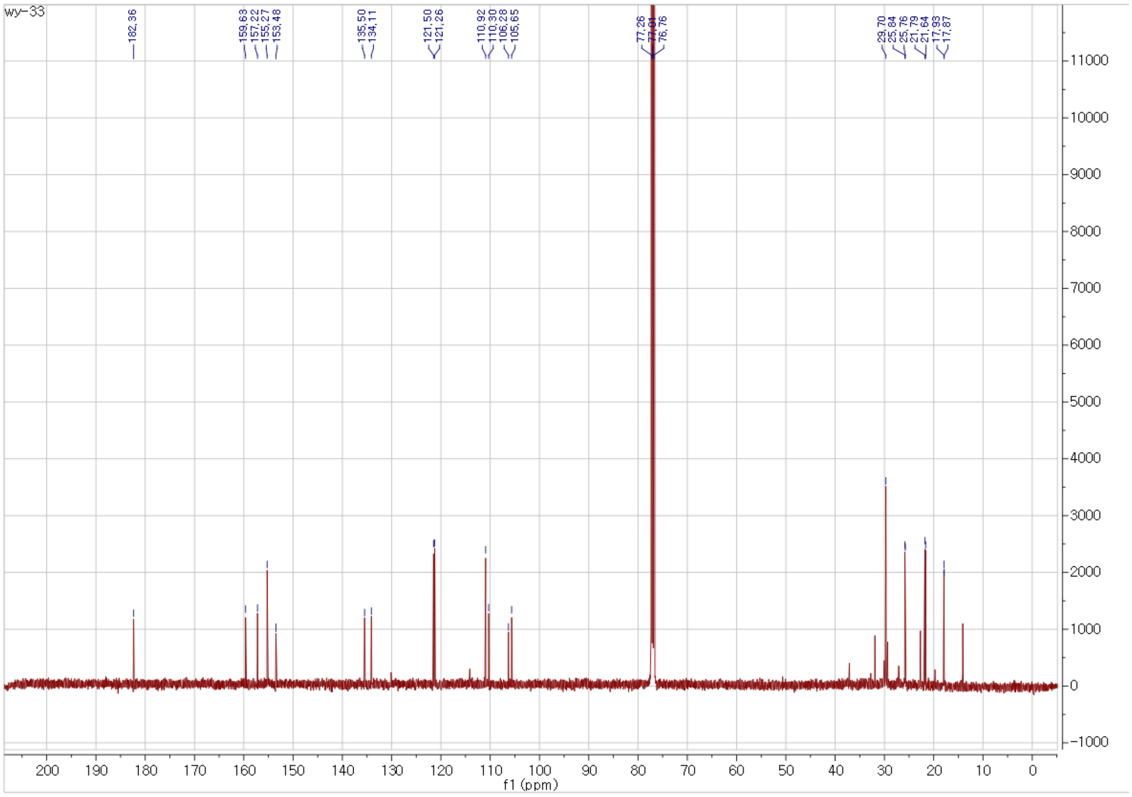

Figure 35. ^{1}H-NMR (500 MHz) and ^{13}C-NMR (125 MHz) spectrums

of compound **18** ($CDCl_3$)

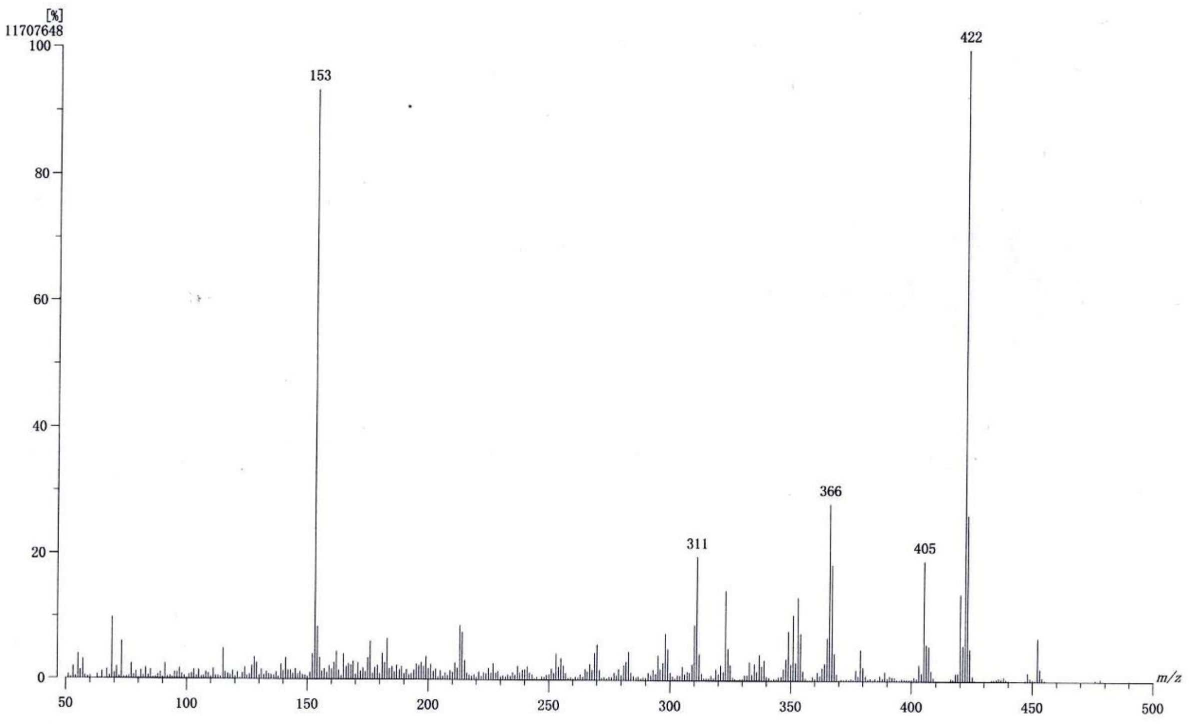

Sample: -
Note : -
Inlet : Direct
Ion Mode : EI+
RT : 2.65 min
Scan#: 54
Elements : C 100/1, H 100/1, O 10/1
Mass Tolerance : 3mmu
Unsaturation (U.S.) : 0.0 - 20.0

Observed m/z	Int%	Err[ppm / mmu]	U.S.	Composition
422.1727	100.0	-0.6 / -0.3	13.0	C 25 H 26 O 6

Figure 36. EIMS and HREIMS data of compound **18**

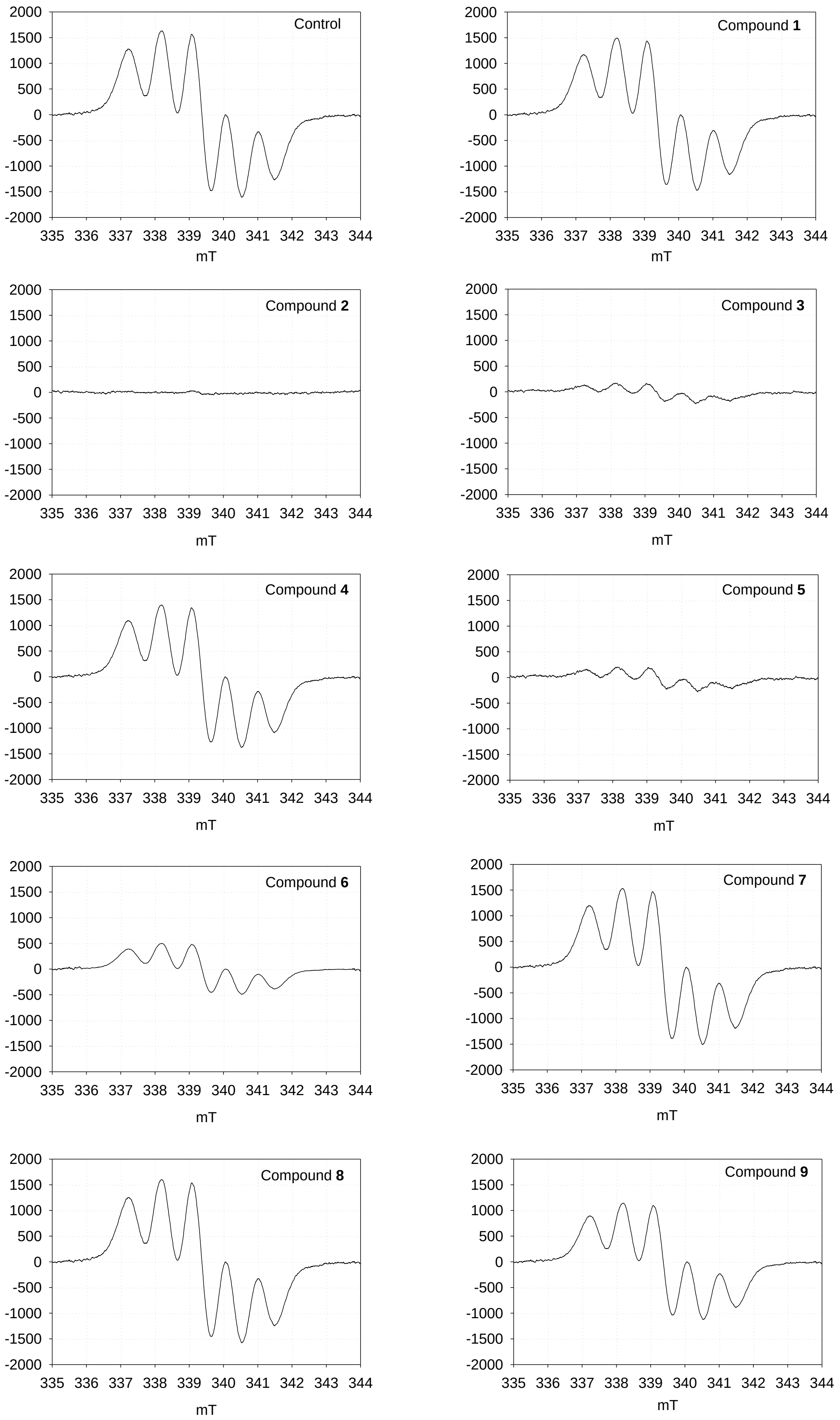

Figure 37. ESR spectra of DPPH radical scavenging effect of compounds**1-9** (7.0 mg/ml)

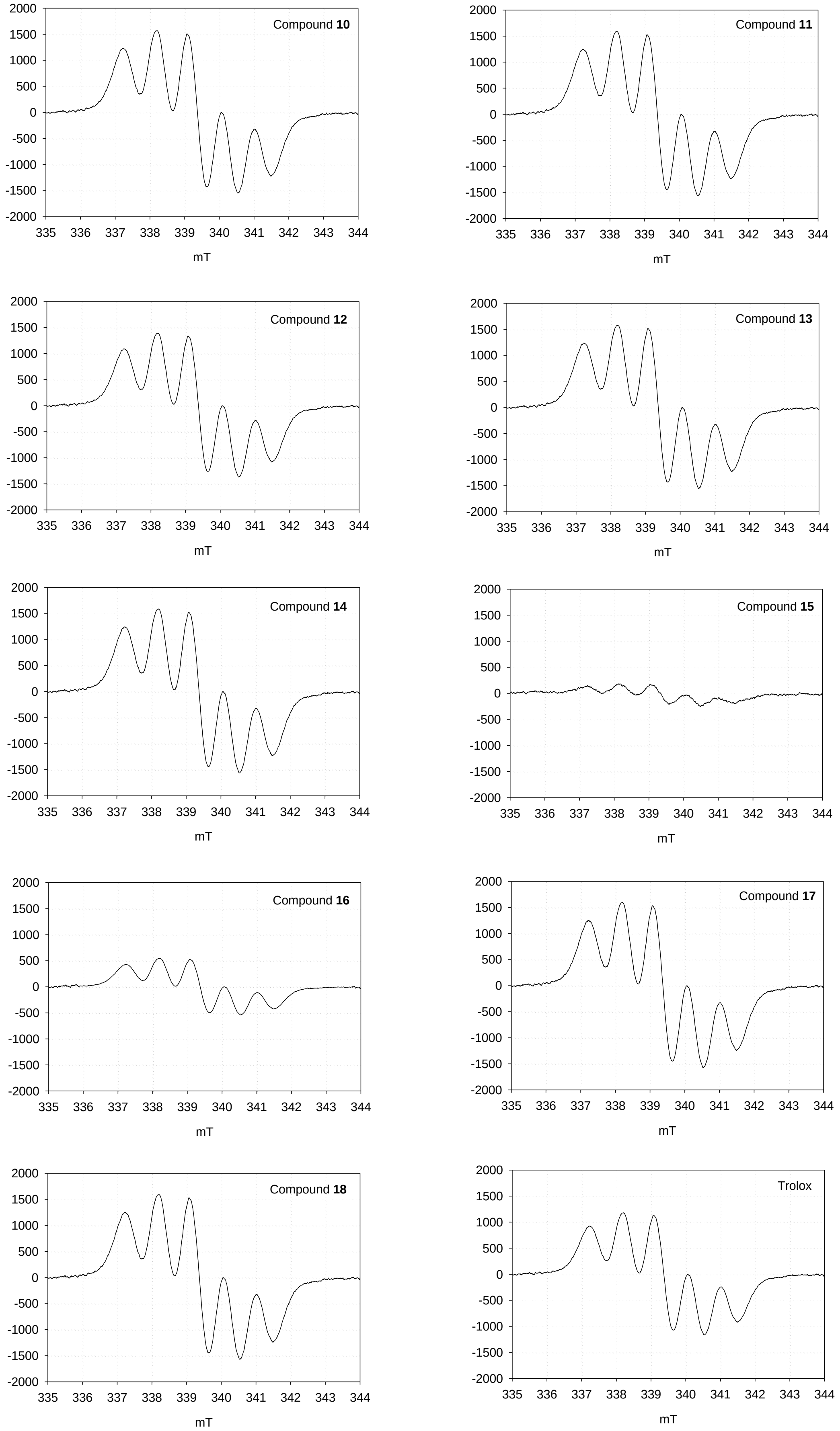

Figure 38. ESR spectra of DPPH radical scavenging effect of compounds**10-18** (7.0 mg/ml)

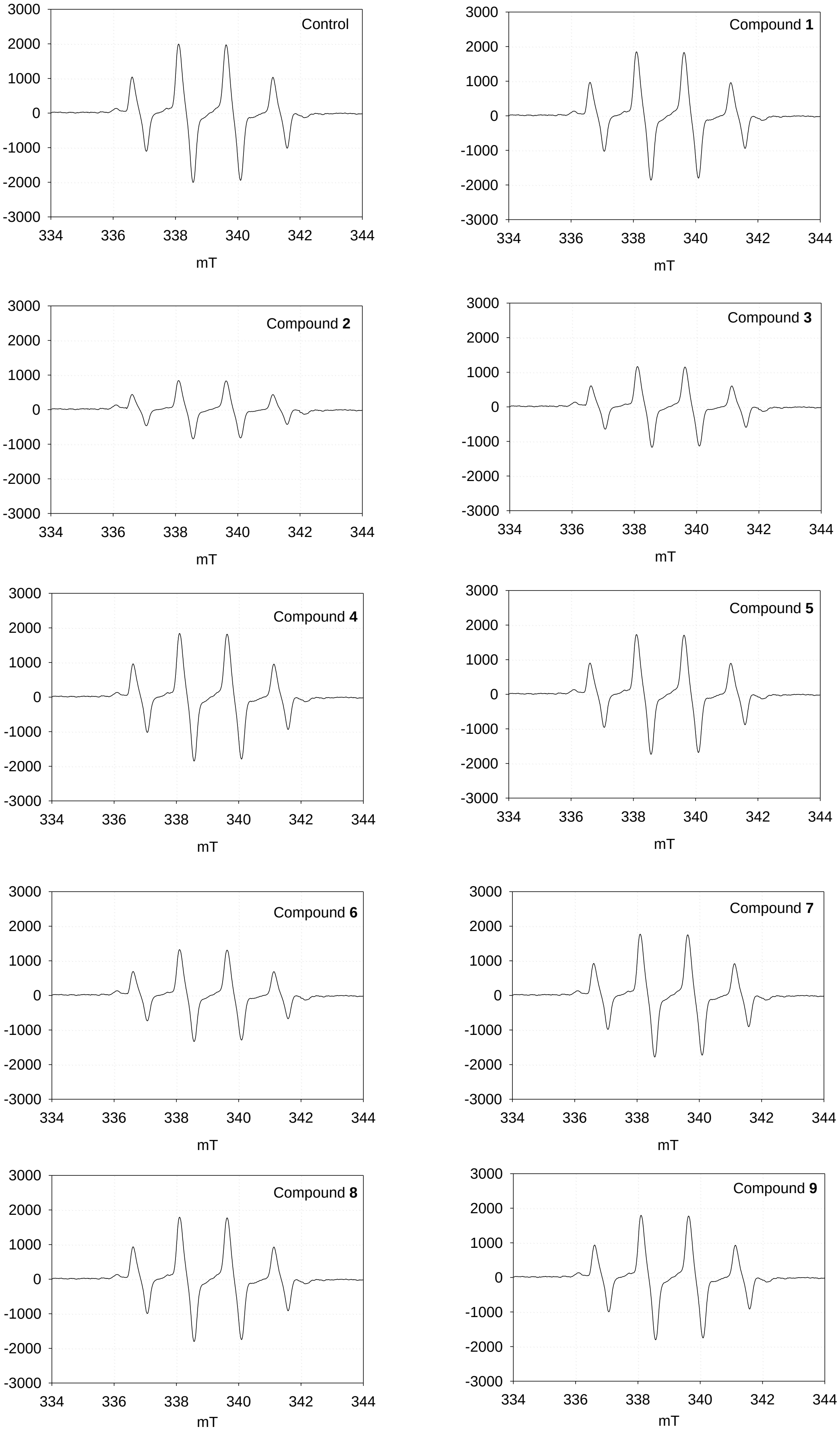

Figure 39. ESR spectra of hydroxyl radical scavenging effect of compounds**1-9** (7.5 mg/ml)

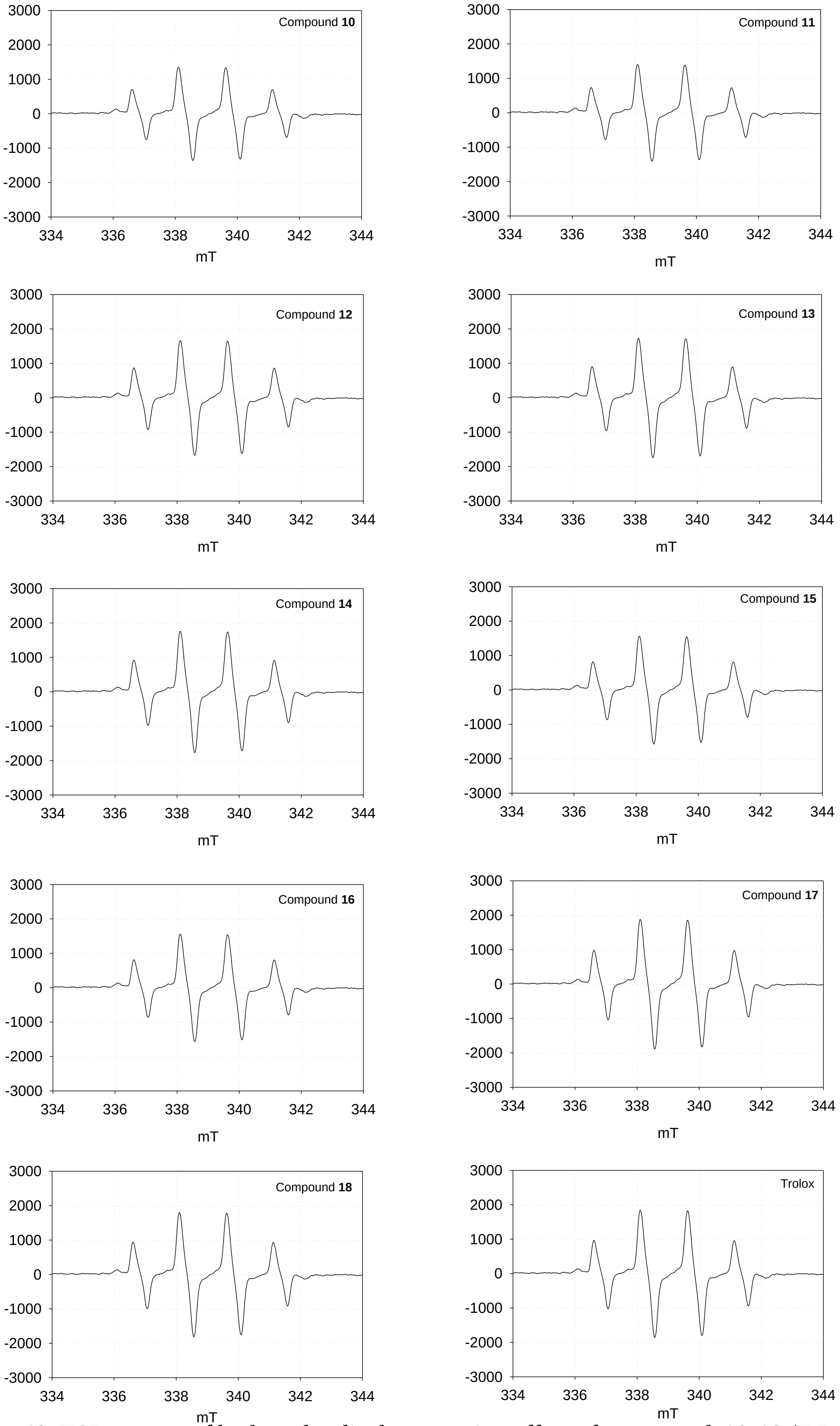

Figure 40. ESR spectra of hydroxyl radical scavenging effect of compounds**10-18** (7.5 mg/ml)

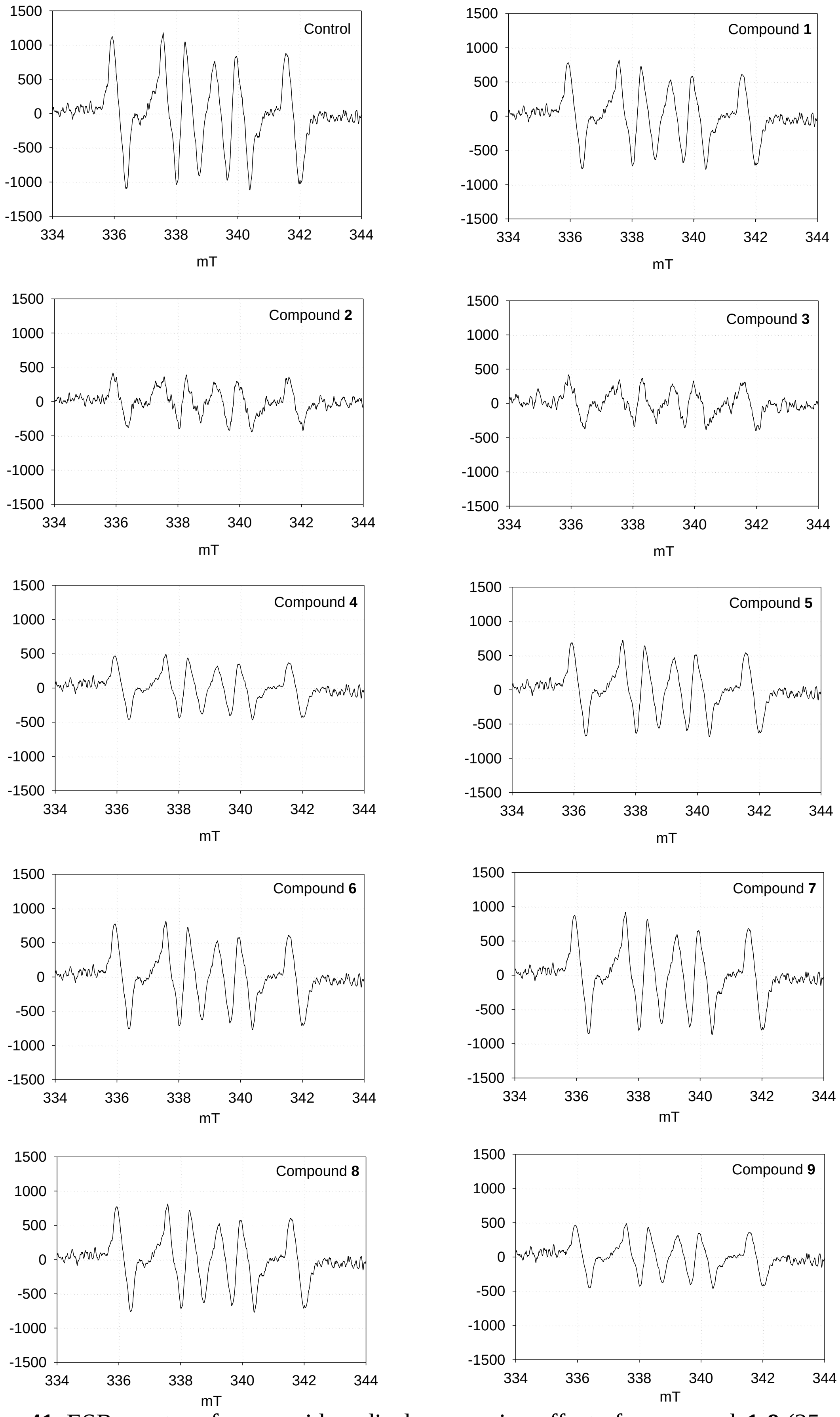

Figure 41. ESR spectra of superoxide radical scavenging effect of compounds**1-9** (25 mg/ml)

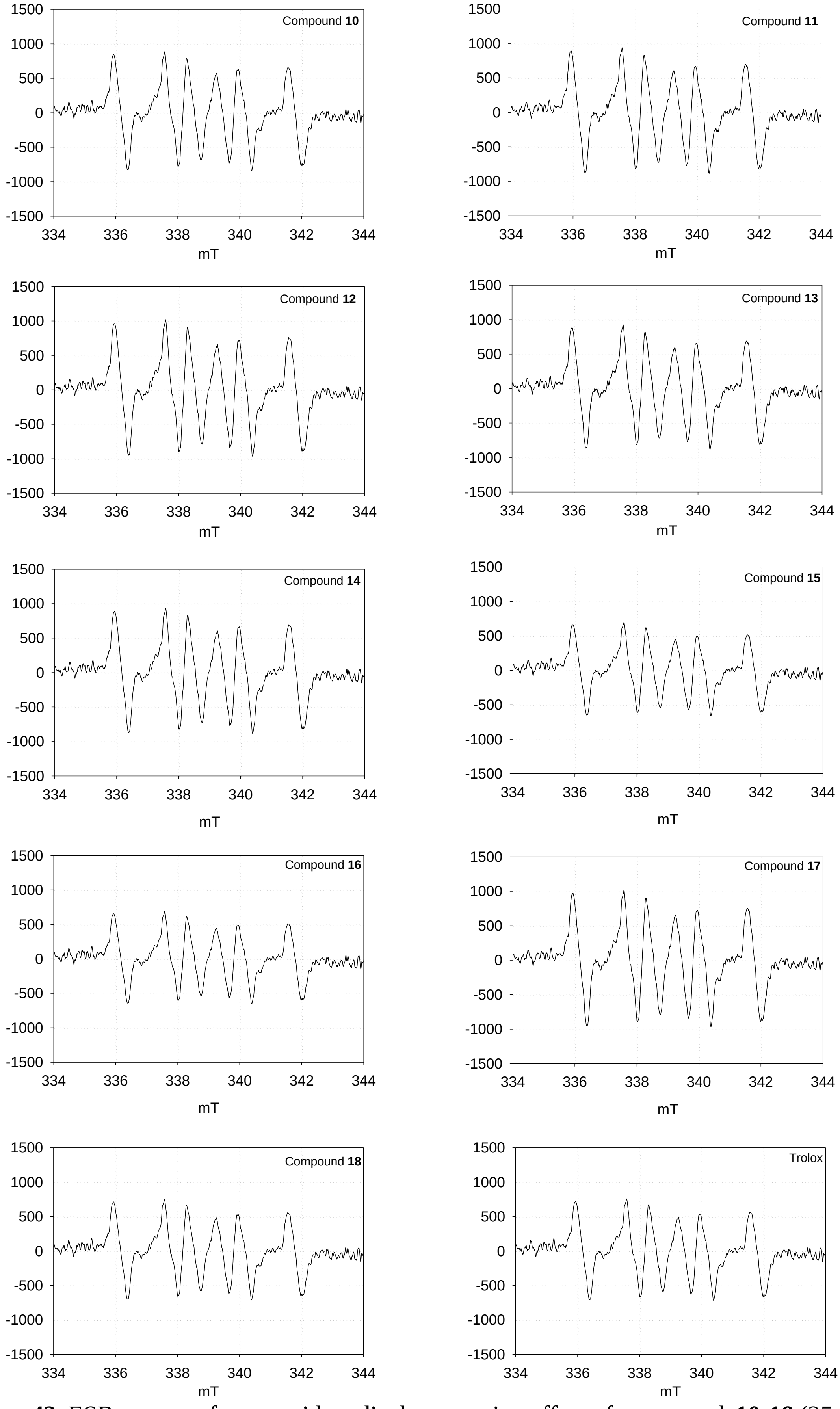

Figure 42. ESR spectra of superoxide radical scavenging effect of compounds**10-18** (25 mg/ml)

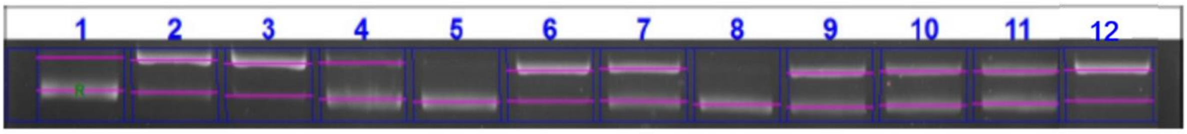

Lane And Band Analysis

Lane 1

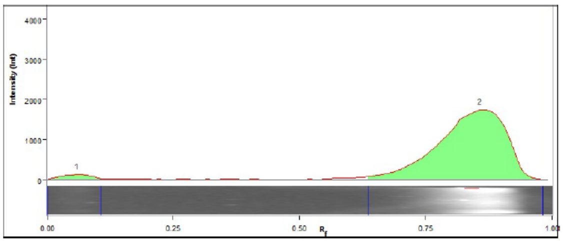

Band No.	Band Label	Mol. Wt. (KDa)	Relative Front	Volume (Int)	Abs. Quant.	Rel. Quant.	Band %	Lane %
1		N/A	0.067	77,343	N/A	0.03	3.0	3.0
2		N/A	0.865	2,472,192	N/A	1.00	97.0	94.6

Lane 2

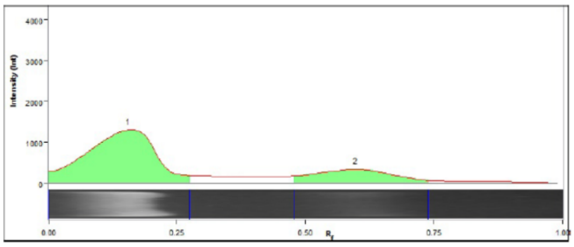

Band No.	Band Label	Mol. Wt. (KDa)	Relative Front	Volume (Int)	Abs. Quant.	Rel. Quant.	Band %	Lane %
1		N/A	0.167	1,767,252	N/A	1.00	77.1	67.8
2		N/A	0.607	399,000	N/A	0.30	22.9	20.1

Lane 3

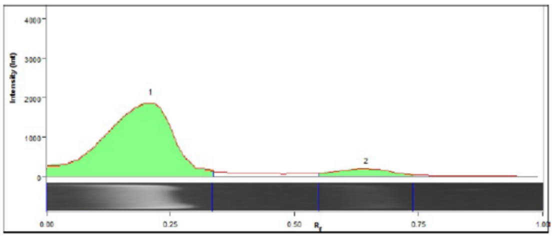

Band No.	Band Label	Mol. Wt. (KDa)	Relative Front	Volume (Int)	Abs. Quant.	Rel. Quant.	Band %	Lane %
1		N/A	0.214	2,155,794	N/A	1.02	89.3	82.4
2		N/A	0.655	215,812	N/A	0.12	10.7	9.9

Lane 4

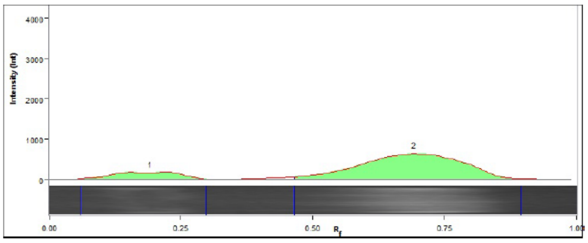

Band No.	Band Label	Mol. Wt. (KDa)	Relative Front	Volume (Int)	Abs. Quant.	Rel. Quant.	Band %	Lane %
1		N/A	0.202	226,134	N/A	0.13	15.3	15.0
2		N/A	0.702	1,251,642	N/A	0.71	84.7	83.1

Lane 5

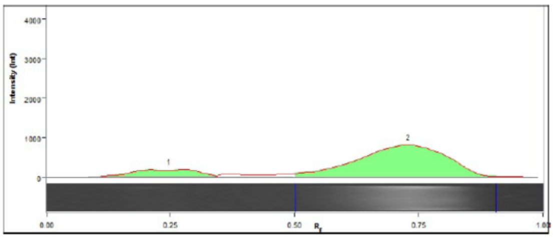

Band No.	Band Label	Mol. Wt. (KDa)	Relative Front	Volume (Int)	Abs. Quant.	Rel. Quant.	Band %	Lane %
1		N/A	0.202	284,152	N/A	0.13	10.4	7.2
2		N/A	0.738	1,471,044	N/A	0.83	87.5	88.2

Lane 6

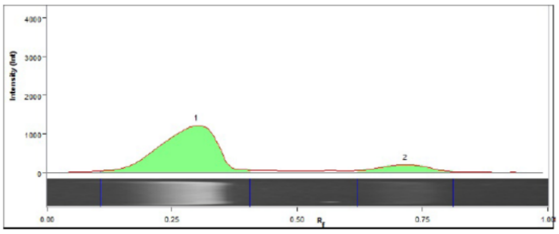

Band No.	Band Label	Mol. Wt. (KDa)	Relative Front	Volume (Int)	Abs. Quant.	Rel. Quant.	Band %	Lane %
1		N/A	0.310	1,998,383	N/A	0.79	87.5	82.5
2		N/A	0.726	198,798	N/A	0.11	12.5	11.8

Lane 7

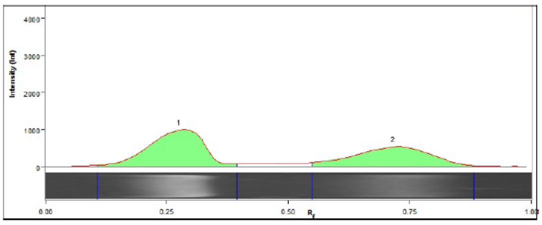

Band No.	Band Label	Mol. Wt. (KDa)	Relative Front	Volume (Int)	Abs. Quant.	Rel. Quant.	Band %	Lane %
1		N/A	0.286	1,133,424	N/A	0.64	56.9	53.9
2		N/A	0.726	858,738	N/A	0.49	43.1	40.8

Lane 8

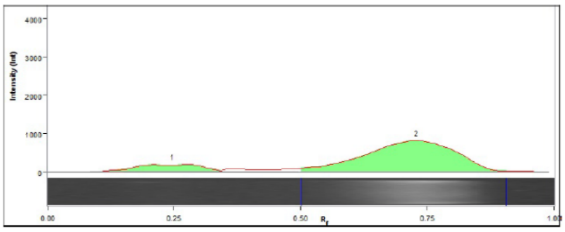

Band No.	Band Label	Mol. Wt. (KDa)	Relative Front	Volume (Int)	Abs. Quant.	Rel. Quant.	Band %	Lane %
1		N/A	0.202	383,045	N/A	0.13	8.8	11.5
2		N/A	0.738	1,251,980	N/A	0.83	92.3	88.2

Lane 9

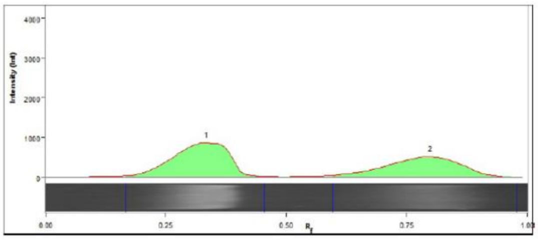

Band No.	Band Label	Mol. Wt. (KDa)	Relative Front	Volume (Int)	Abs. Quant.	Rel. Quant.	Band %	Lane %
1		N/A	0.345	1,504,484	N/A	0.58	56.3	55.3
2		N/A	0.810	793,560	N/A	0.45	43.7	43.0

Lane 10

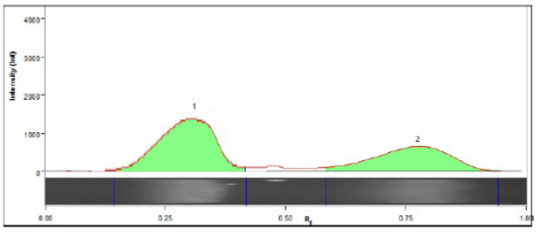

Band No.	Band Label	Mol. Wt. (KDa)	Relative Front	Volume (Int)	Abs. Quant.	Rel. Quant.	Band %	Lane %
1		N/A	0.321	1,789,887	N/A	0.60	53.8	50.2
2		N/A	0.786	1,067,184	N/A	0.52	46.2	43.2

Lane 11

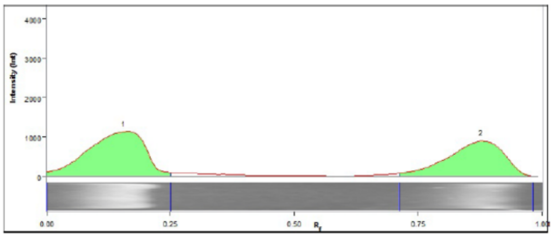

Band No.	Band Label	Mol. Wt. (KDa)	Relative Front	Volume (Int)	Abs. Quant.	Rel. Quant.	Band %	Lane %
1		N/A	0.163	1,800,574	N/A	0.57	56.8	54.0
2		N/A	0.885	1,115,133	N/A	0.43	43.2	41.1

Figure 43. Results of electrophoresis by pBR322 plasmid DNA band intensity; Lane 1, pBR322 plasmid DNA; Lane 2, oxidative DNA; Lane 3, compound **1**; Lane 4, compound **2**; Lane 5, compound **3**; Lane 6, compound **4**; Lane 7, compound **5**; Lane 8, compound **6**; Lane 9, compound **7**; Lane 10, compound **8**; Lane 11, compound **9**.

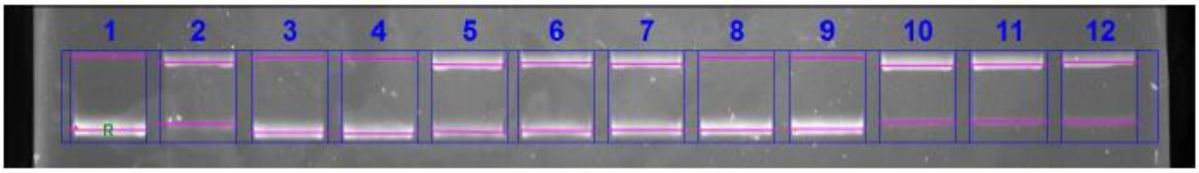

Lane 3

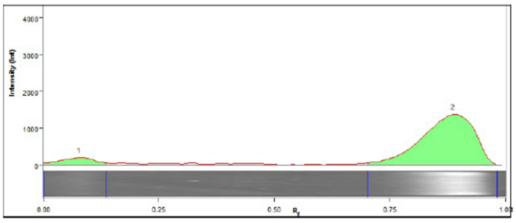

Band No.	Band Label	Mol. Wt. (KDa)	Relative Front	Volume (Int)	Abs. Quant.	Rel. Quant.	Band %	Lane %
1		N/A	0.087	384,875	N/A	0.06	8.6	8.0
2		N/A	0.894	1,648,911	N/A	0.67	91.4	85.1

Lane 4

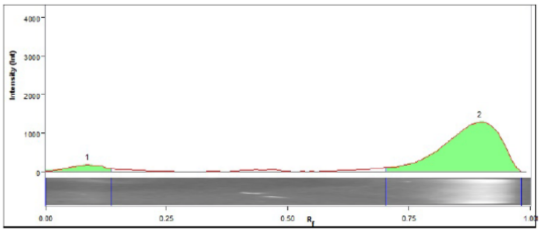

Band No.	Band Label	Mol. Wt. (KDa)	Relative Front	Volume (Int)	Abs. Quant.	Rel. Quant.	Band %	Lane %
1		N/A	0.096	430,499	N/A	0.05	7.6	7.1
2		N/A	0.904	1,619,940	N/A	0.66	92.4	85.9

Lane 5

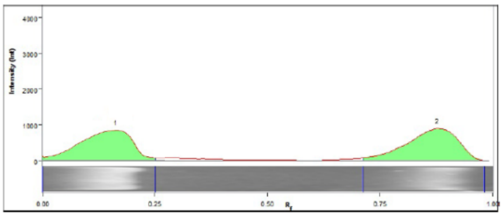

Band No.	Band Label	Mol. Wt. (KDa)	Relative Front	Volume (Int)	Abs. Quant.	Rel. Quant.	Band %	Lane %
1		N/A	0.163	914,188	N/A	0.43	43.2	41.1
2		N/A	0.885	1,074,885	N/A	0.57	56.8	54.0

Lane 6

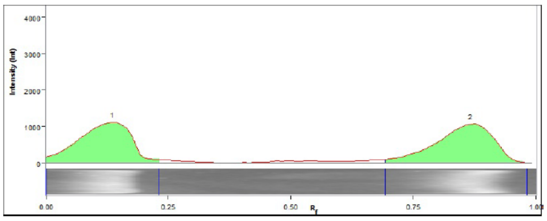

Band No.	Band Label	Mol. Wt. (KDa)	Relative Front	Volume (Int)	Abs. Quant.	Rel. Quant.	Band %	Lane %
1		N/A	0.144	1,273,941	N/A	0.52	49.2	46.6
2		N/A	0.875	1,315,875	N/A	0.53	50.8	48.1

Lane 7

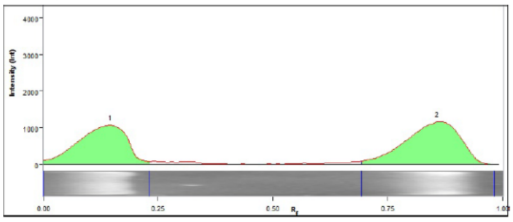

Band No.	Band Label	Mol. Wt. (KDa)	Relative Front	Volume (Int)	Abs. Quant.	Rel. Quant.	Band %	Lane %
1		N/A	0.154	1,218,609	N/A	0.49	45.5	43.4
2		N/A	0.865	1,462,557	N/A	0.59	54.5	52.0

Lane 8

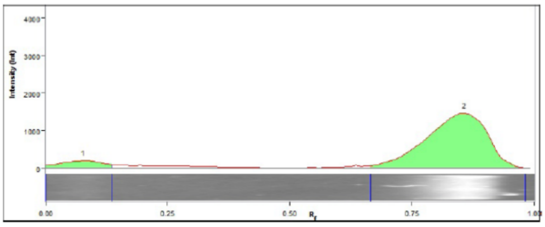

Band No.	Band Label	Mol. Wt. (KDa)	Relative Front	Volume (Int)	Abs. Quant.	Rel. Quant.	Band %	Lane %
1		N/A	0.087	532,171	N/A	0.07	8.5	8.0
2		N/A	0.865	1,919,829	N/A	0.78	91.5	85.7

Lane 9

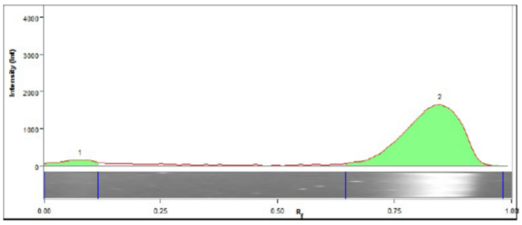

Band No.	Band Label	Mol. Wt. (KDa)	Relative Front	Volume (Int)	Abs. Quant.	Rel. Quant.	Band %	Lane %
1		N/A	0.087	505,487	N/A	0.05	5.6	5.3
2		N/A	0.856	2,153,511	N/A	0.87	94.4	89.0

Lane 10

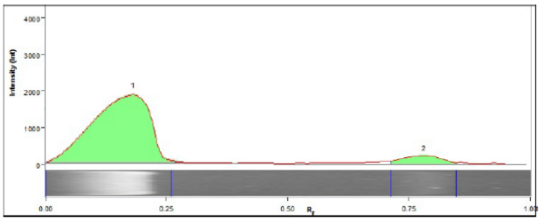

Band No.	Band Label	Mol. Wt. (KDa)	Relative Front	Volume (Int)	Abs. Quant.	Rel. Quant.	Band %	Lane %
1		N/A	0.173	2,300,434	N/A	0.61	88.1	82.1
2		N/A	0.788	204,798	N/A	0.08	11.9	11.1

Lane 11

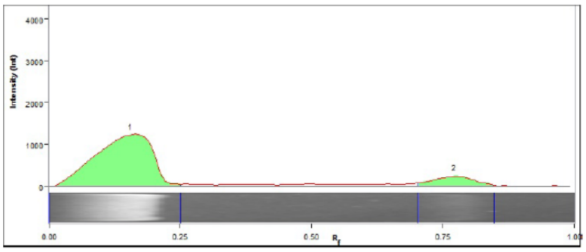

Band No.	Band Label	Mol. Wt. (KDa)	Relative Front	Volume (Int)	Abs. Quant.	Rel. Quant.	Band %	Lane %
1		N/A	0.163	1,847,533	N/A	0.57	88.1	80.5
2		N/A	0.779	190,791	N/A	0.08	11.9	10.8

Lane 12

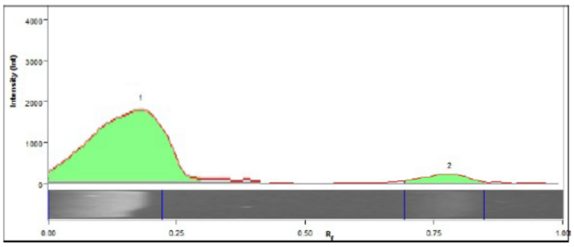

Band No.	Band Label	Mol. Wt. (KDa)	Relative Front	Volume (Int)	Abs. Quant.	Rel. Quant.	Band %	Lane %
1		N/A	0.135	2,000,494	N/A	0.43	83.4	78.0
2		N/A	0.788	213,150	N/A	0.09	16.6	15.6

Figure 44. Results of electrophoresis by pBR322 plasmid DNA band intensity; Lane 3, compound **10**; Lane 4, compound **11**; Lane 5, compound **12**; Lane 6, compound **13**; Lane 7, compound **14**; Lane 8, compound **15**; Lane 9, compound **16**; Lane 10, compound **17**; Lane 11, compound **18**; Lane 12, trolox.

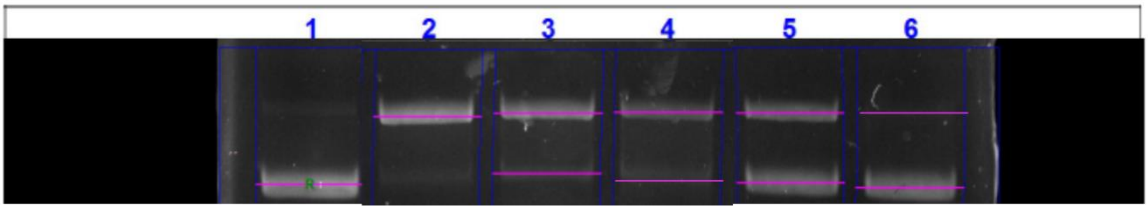

Lane 1

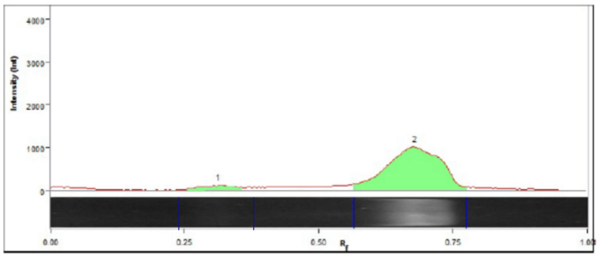

Band No.	Band Label	Mol. Wt. (KDa)	Relative Front	Volume (Int)	Abs. Quant.	Rel. Quant.	Band %	Lane %
1		N/A	0.329	21,489	N/A	0.06	2.64	1.2
2		N/A	0.675	2,854,032	N/A	0.87	89.9	92.2

Lane 2

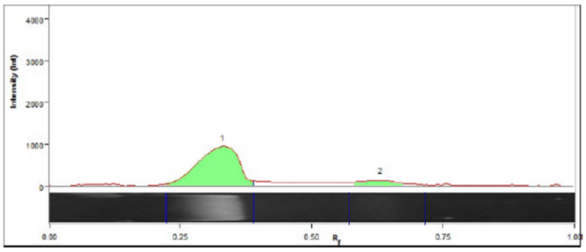

Band No.	Band Label	Mol. Wt. (KDa)	Relative Front	Volume (Int)	Abs. Quant.	Rel. Quant.	Band %	Lane %
1		N/A	0.317	2,488,197	N/A	0.49	83.3	70.0
2		N/A	0.613	104,681	N/A	0.08	14.1	11.8

Lane 3

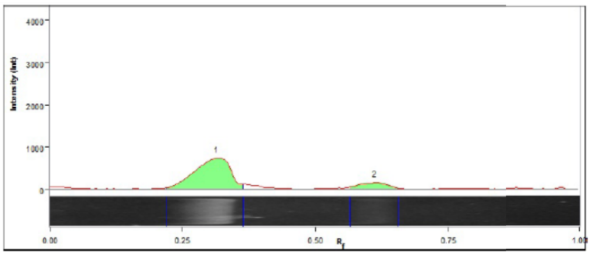

Band No.	Band Label	Mol. Wt. (KDa)	Relative Front	Volume (Int)	Abs. Quant.	Rel. Quant.	Band %	Lane %
1		N/A	0.317	2,124,835	N/A	0.49	83.3	70.0
2		N/A	0.613	501,365	N/A	0.08	14.1	11.8

Lane 4

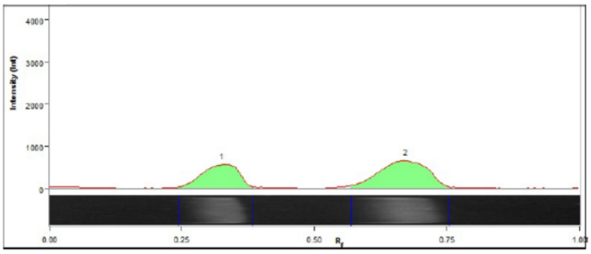

Band No.	Band Label	Mol. Wt. (KDa)	Relative Front	Volume (Int)	Abs. Quant.	Rel. Quant.	Band %	Lane %
1		N/A	0.329	1,474,330	N/A	0.39	39.4	36.4
2		N/A	0.675	1,033,238	N/A	0.60	60.6	56.1

Lane 5

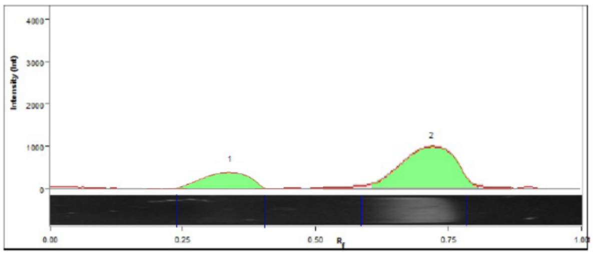

Band No.	Band Label	Mol. Wt. (KDa)	Relative Front	Volume (Int)	Abs. Quant.	Rel. Quant.	Band %	Lane %
1		N/A	0.087	780,512	N/A	0.23	28.8	20.5
2		N/A	0.894	1,844,338	N/A	0.67	66.2	71.7

Lane 6

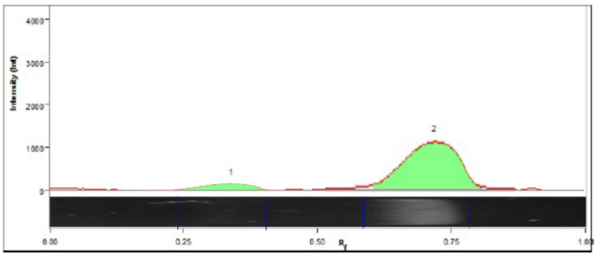

Band No.	Band Label	Mol. Wt. (KDa)	Relative Front	Volume (Int)	Abs. Quant.	Rel. Quant.	Band %	Lane %
1		N/A	0.087	312,394	N/A	0.06	8.6	8.0
2		N/A	0.894	2,740,371	N/A	0.67	91.4	85.1

Figure 45. Results of electrophoresis by pBR322 plasmid DNA band intensity against dose-dependent with compound **2**; Lane 1, pBR322 plasmid DNA (control); Lane 2, oxidative DNA; Lane 3, 7.5 μM; Lane 4, 15.0 μM; Lane 5, 30.0 μM; Lane 6, 60.0 μM.

Table 1. pBR322 plasmid DNA damage protective effect of **1-18**.

	scDNA band intensity	ocDNA band intensity	DNA damage (%)	DNA protective effect (%)
pBR322 DNA	2472.2	77.3	-	-
oxidative DNA	399.2	1767.2	-	-
**1**	215.8	2155.8	87.2	12.8
**2**	1251.6	226.1	9.1	90.9
**3**	1471.0	284.15	11.5	88.5
**4**	198.8	1998.4	80.8	19.2
**5**	858.7	1133.4	45.8	54.2
**6**	1250.2	383.1	15.5	84.5
**7**	793.5	1504.5	60.9	39.1
**8**	1067.2	1789.9	72.4	27.6
**9**	1115.1	1800.6	72.8	27.2
**10**	1648.9	384.9	15.6	84.4
**11**	1619.9	430.5	17.4	82.6
**12**	1074.9	914.2	37.0	63.0
**13**	1315.9	1273.9	51.5	48.5
**14**	1462.6	1218.6	49.3	50.7
**15**	1919.8	532.2	21.5	78.5
**16**	2153.5	505.5	20.4	79.6
**17**	204.8	2300.5	93.1	6.9
**18**	190.8	1847.5	74.7	25.3
Trolox	213.2	2000.5	80.9	19.1

Table 2. Protective effect of pBR322 plasmid DNA by dose dependent of compound **2**

	scDNA band intensity	ocDNA band intensity	DNA damage (%)	DNA protective effect (%)
pBR322 DNA	2854.0	21.5	-	-
oxidative DNA	104.7	2488.2	-	-
7.5 μM	501.4	2125.4	74.5	25.5
15.0 μM	1033.2	1494.3	52.4	47.6
30.0 μM	1844.3	780.5	27.3	72.7
60.0 μM	2740.4	312.4	10.9	89.1
